# Differential Effects of Varying Concentrations of Phosphorus, Iron, and Nitrogen in N_2_-Fixing Cyanobacteria

**DOI:** 10.3389/fmicb.2020.541558

**Published:** 2020-09-25

**Authors:** Víctor Fernández-Juárez, Antoni Bennasar-Figueras, Antoni Sureda-Gomila, Guillem Ramis-Munar, Nona S. R. Agawin

**Affiliations:** ^1^Marine Ecology and Systematics (MarES), Department of Biology, University of the Balearic Islands, Palma, Spain; ^2^Grup de Microbiologia, Department of Biology, University of the Balearic Islands, Palma, Spain; ^3^Research Group on Community Nutrition and Oxidative Stress, University of the Balearic Islands and CIBEROBN (Fisiopatología de la Obesidad y la Nutrición), Palma, Spain; ^4^Cellomic Unit of University Institute of Research in Health Sciences of the Balearic Islands, Palma, Spain

**Keywords:** *Halothece* sp. PCC 7418, *Fischerella muscicola* PCC 73103, phosphorus, iron, nitrogen, N_2_-fixation, reactive oxygen species production, Pho–Fur–NtcA regulon

## Abstract

Diazotrophs or N_2_-fixers are one of the most ecologically significant groups in marine ecosystems (pelagic and benthic). Inorganic phosphorus (PO_4_^3–^) and iron (Fe) can limit the growth and N_2_-fixing capacities of cyanobacteria. However, studies investigating co-limitation of these factors are lacking. Here, we added different concentrations of PO_4_^3–^ and Fe in two cyanobacterial species whose relatives can be found in seagrass habitats: the unicellular *Halothece* sp. (PCC 7418) and the filamentous *Fischerella muscicola* (PCC 73103), grown under different nitrate (NO_3_^–^) concentrations and under N_2_ as sole N source, respectively. Their growth, pigment content, N_2_-fixation rates, oxidative stress responses, and morphological and cellular changes were investigated. Our results show a serial limitation of NO_3_^–^ and PO_4_^3–^ (with NO_3_^–^ as the primary limiting nutrient) for *Halothece* sp. Simultaneous co-limitation of PO_4_^3–^ and Fe was found for both species tested, and high levels of Fe (especially when added with high PO_4_^3–^ levels) inhibited the growth of *Halothece* sp. Nutrient limitation (PO_4_^3–^, Fe, and/or NO_3_^–^) enhanced oxidative stress responses, morphological changes, and apoptosis. Furthermore, an extensive bio-informatic analysis describing the predicted Pho, Fur, and NtcA regulons (involved in the survival of cells to P, Fe, and N limitation) was made using the complete genome of *Halothece* sp. as a model, showing the potential of this strain to adapt to different nutrient regimes (P, Fe, or N).

## Introduction

Diazotrophs or N_2_-fixers, one of the most important ecological groups, are microorganisms capable of converting atmospheric dinitrogen gas (N_2_) into a readily usable form of dissolved inorganic nitrogen, i.e., ammonia (NH_3_). This process is carried out through the nitrogenase protein complex (encoded by *nifH*, *nifD*, and *nifK*) by nitrogenase-containing autotrophic and heterotrophic bacteria (Hoffman et al., 2014). Biological N_2_-fixation is an essential process in the marine N-cycle, providing new N to the oceans and compensating ocean denitrification (Sohm et al., 2011). In coastal benthic communities (e.g., Mediterranean seagrass *Posidonia oceanica* communities), N_2_-fixers are also important sources of new N, potentially supplying the entire demand of N of the associated plants (Agawin et al., 2016, 2017). These benthic communities can harbor a high diversity of N_2_-fixing cyanobacteria among which are different groups of cyanobacteria that can be classified as unicellular, filamentous heterocyst forming, and filamentous non-heterocyst forming (Zehr, 2011).

Factors affecting the growth and abundance of cyanobacteria include both abiotic [e.g., macronutrients (e.g., phosphorus, P, and nitrogen, N), micronutrients (e.g., iron, Fe), light, and temperature] and biotic factors (e.g., viral cell lysis or grazing) (Sohm et al., 2011; Zehr, 2011). The nutrients P, N, and Fe are suggested to be the most important liming nutrients for cyanobacterial growth (Mills et al., 2004; Browning et al., 2017). Phosphorus (P) has key structural (i.e., for nucleic acids and for the cell membranes of most bacteria) and functional roles in various metabolic processes and limiting concentrations of P affect photosynthesis, respiration, and activities of ATP-dependent enzymes (Tripathi et al., 2013; Santos-Beneit, 2015). Nitrogen is essential for protein synthesis, and limiting concentrations of N can reduce cellular growth rates and contribute to the phenomenon called chlorosis, which causes degradation of phycobiliproteins and can lead to posterior downregulation of photosynthesis (Richaud et al., 2001; Klotz et al., 2015). Iron (Fe) is also important for photosynthesis and growth (Sunda and Huntsman, 2015); however, cells must maintain a state of homeostasis since elevated Fe-intracellular concentrations can enhance oxidative stress via Fenton and Haber–Weiss reactions (Kranzler et al., 2013). Under Fe limitation, cyanobacteria can import Fe into the cells through various high-affinity Fe transporters (Kranzler et al., 2014) or through the liberation of low-molecular-weight Fe chelators (e.g., siderophores) (Kranzler et al., 2013). In order to survive and adapt in limiting nutrient conditions, cyanobacteria must trigger molecular mechanisms by transcription factors (TFs). The most well-studied TFs in marine bacteria are PhoB, ferric uptake (Fur), and global nitrogen (NtcA) regulators involved in the acquisition of P, Fe, and N, respectively (Baichoo and Helmann, 2002; Yuan et al., 2006; Llacer et al., 2010). The PhoB, together with PhoR, an inner-membrane histidine kinase, forms a two-component regulatory system (Santos-Beneit, 2015). These TFs are activated or repressed to adapt to a changing environment (e.g., changes in nutrient concentrations) and recognize consensus sequences called transcriptional binding sites (TBS), namely, the PHO box, Fur, and NtcA boxes (Baichoo and Helmann, 2002; Yuan et al., 2006; Llacer et al., 2010), which control the expression of specific genes. The genes controlled by these TFs (PhoB, Fur, or NtcA) are part of what is known as the Pho, Fur, and NtcA regulons, and their prediction can help us understand how cells may adapt and survive under limiting concentrations of P, Fe, and N, respectively.

The demand for P, Fe, and N for N_2_-fixing cyanobacteria may be different from that for non-N_2_-fixing bacteria. Generally, cyanobacteria have elevated ratio of nitrogen (N):phosphorus (P) (a molar ratio above 25 compared with the general Redfield ratio of 16 in marine phytoplankton) (Redfield, 1934; Geider and La Roche, 2002; Quigg et al., 2011). For cyanobacteria, Fe requirements are 10-fold higher than in non-photosynthetic bacteria (Kranzler et al., 2013). For cyanobacterial N_2_-fixers, Fe is even more important, as Fe is an important structural component of the nitrogenase complex (i.e., *nifH*) (Hoffman et al., 2014). N_2_-fixation also requires a large amount of photosynthetically derived energy, which in turn is P-dependent (Sañudo-Wilhelmy et al., 2001). While non-N_2_-fixing cyanobacteria are usually limited by N, N_2_-fixing cyanobacteria can use ubiquitous atmospheric N_2_, conferring them a competitive advantage over non-N_2_-fixing cyanobacteria in N-limited waters (Sohm et al., 2011). Usually, under limiting concentrations of inorganic N (e.g., NH_3_ and NO_3_^–^), N_2_-fixation is stimulated, while high concentrations of inorganic N inhibit N_2_-fixation (Manhart and Wong, 1980; Nelson et al., 1982; Knapp, 2012). In a previous study, the P-requirements of *Halothece* sp. PCC 7418 increased in N-limited conditions (i.e., when N_2_-fixation was stimulated), consequently activating their P-acquisition mechanisms [i.e., alkaline phosphatase activity (APA) and P-uptake], which were in turn controlled by Fe availability (Fernández-Juárez et al., 2019). How the concentrations of P and Fe (and their interacting effects) affect the different groups of N_2_-fixing cyanobacteria grown in differing N conditions remains to be investigated.

Here, we investigated the response to different concentrations of PO_4_^3–^ and Fe, in a multi-factorial design, of two species of N_2_-fixing cyanobacteria: the unicellular *Halothece* sp. PCC 7418 and the filamentous heterocyst-forming *Fischerella muscicola* PCC 73103, grown under different nitrate (NO_3_^–^) concentrations and under N_2_ as sole N source, respectively. Relatives of these two cyanobacteria have been previously found in association with the seagrass *P. oceanica* (Supplementary Material, Supplementary Figure S1, Agawin et al., 2017). The responses were measured in terms of physiological and molecular parameters (growth, pigment content, N_2_-fixation rates, oxidative stress, morphology, and apoptosis). To understand how *Halothece* sp. (PCC 7418) adapts in nutrient limiting environments, an extensive *in silico* analysis was done, taking as a model the complete genome of *Halothece* sp. to predict the Pho, Fur, and NtcA boxes, and the Pho, Fur, and NtcA regulons, respectively.

## Materials and Methods

### Strains and Growth Conditions

N_2_-fixing bacterial strains (*Halothece* sp. PCC 7418 and *F. muscicola* sp. PCC 73103) were obtained from Pasteur Culture Collection of Cyanobacteria (PCC). Experiments were conducted in 250-ml acid-clean quartz Erlenmeyer flasks filled with 150 ml of their corresponding culture media. We used ASN-III + Turks Island salts 4× medium and BG11_0_ culture medium for *Halothece* sp. and *F. muscicola*, respectively (Stanier et al., 1979). These media were supplemented with 0.1–0.3% (p/v) glucose, and cells were cultivated under aerobic condition in 12:12-h light–dark cycle at 25°C and under continuous low-intensity fluorescent light (≅30 μE m^–2^ s^–1^) in a rotatory shaker (120 rpm). The batch cultures were maintained for over 10–14 days for each experiment, and the initial inoculum of cells was added at the exponential phase [O.D._750 nm/975 nm_ ≅ 0.2 (*Halothece* sp.)/1.0 (*F. muscicola*)] from their original culture medium. All samples were manipulated in a class-100 clean hood to avoid Fe contamination. The subcultures for seeding were centrifuged and washed with their corresponding medium without PO_4_^3–^, Fe, and NO_3_^–^.

For *Halothece* sp., three conditions were established for PO_4_^3–^ concentrations: [Low PO_4_^3–^] (0.1 μM), [Medium PO_4_^3–^] (1 μM), and [High PO_4_^3–^] (45 μM). Furthermore, three conditions for Fe were also established: [Low Fe] (2 nM), [Medium Fe] (20 nM), and [High Fe] (7.5 μM). These PO_4_^3–^ and Fe concentrations were combined in nine experimental conditions ([Low PO_4_^3–^–Low Fe], [Low PO_4_^3–^–Medium Fe], [Low PO_4_^3–^–High Fe], [Medium PO_4_^3–^–Low Fe], [Medium PO_4_^3–^–Medium Fe], [Medium PO_4_^3–^–High Fe], [High PO_4_^3–^–Low Fe], [High PO_4_^3–^–Medium Fe], and [High PO_4_^3–^–High Fe]) (Supplementary Table S1). The solutions of PO_4_^3–^, Fe, and NO_3_^–^ were prepared from dipotassium phosphate (K_2_HPO_4_), ferric citrate (C_6_H_5_FeO_7_), and sodium nitrate (NaNO_3_), respectively. As reference for the nutrient concentrations for [Low PO_4_^3–^] and [Low Fe], we followed the surface water concentrations of the Mediterranean Sea (Statham and Hart, 2005; Powley et al., 2017), while for [High PO_4_^3–^] and [High Fe], we followed the optimal concentrations of ASN-III + Turks Island salts 4× medium. We performed two sets of experiments following Fernández-Juárez et al. (2019): under 4.4 mM of NO_3_^–^ (optimal concentration) and 0.15 mM of NO_3_^–^ (referred to from now on as [Low NO_3_^–^] and represents the minimum amount of NO_3_^–^ in which *Halothece* sp. grew with maximal N_2_-fixation rates). The cells were incubated for 10 days in these experiments (Supplementary Table S1). After these experiments, we selected the experimental conditions – [Low PO_4_^3–^–Low Fe], [Low PO_4_^3–^–High Fe], [High PO_4_^3–^–Low Fe], and [High PO_4_^3–^–High Fe] – in which *Halothece* sp. cells were grown under extremely limiting NO_3_^–^ concentrations (6.66 nM), following NO_3_^–^ concentrations in the Mediterranean Sea as reference (Powley et al., 2017; Supplementary Table S1). The cultures were maintained over 12 days. To check the reversibility of the phenotypic characteristics of the cells, we re-inoculated with PO_4_^3–^, Fe, and/or NO_3_^–^ in optimal concentrations (PO_4_^3–^: 45 μM, Fe: 7.5 μM, and NO_3_^–^: 4.4 mM) at day 12. Inorganic phosphorus (PO_4_^3–^) was added to [Low PO_4_^3–^–Low Fe], and NO_3_^–^ was added to [High PO_4_^3–^–High Fe] in extremely limiting NO_3_^–^ treatments; and PO_4_^3–^ and Fe were added to [Low PO_4_^3–^–Low Fe] in optimal NO_3_^–^ treatments. These cultures were maintained for over 4 days more (Supplementary Table S1).

For *F. muscicola*, PO_4_^3–^ and Fe were added in nine combination treatments as previously described for *Halothece* sp. but using 180 μM for [High PO_4_^3–^] and 30 μM for [High Fe]. These optimal concentrations for PO_4_^3–^ and Fe are recommended for BG11_0_ medium (Stanier et al., 1979). These PO_4_^3–^ and Fe combinations were performed without any combined N source, and cultures were maintained over 14 days.

All cultures with their respective treatment conditions were performed in duplicate, and growth, pigment content, N_2_-fixation rates, reactive oxygen species (ROS) production and morphological and/or cellular response (e.g., apoptosis) were measured. For *Halothece* sp., subsamples of the culture (1.5 ml) were taken from the culture flasks at the final stage of the experiment and were counted through flow cytometric analysis to normalize the results per cell and for the phenotype recovery experiments. For *F. muscicola*, results were normalized per total chlorophyll (Chl).

### Growth Measurement

The growth of *Halothece* sp. and *F. muscicola* was measured by optical density (O.D.) using a quartz cuvette and read in Cary Eclipse spectrometer. Growth measurements were measured in *Halothece* sp. every 2 days at 750 nm. In *F. muscicola*, we measured O.D. at 975 nm (maximum O.D. of the cells), and this was measured every 2–4 days. Growth rate (μ, day^–1^) and generation time (*T*_g_, days) were calculated following the formula described in Widdel (2010) and Ng et al. (2015):

(1) $$\mu =\frac{Ln\left(O.D.f\right)-Ln\left(O.D.i\right)}{T\,f-T\,i}$$

(2) $$T<cps:sub<g</cps:sub<=\frac{L\,n\,\left(2\right)}{\mu }$$

where *O.D._f_* is the O.D. at the final time (*Tf*) and *O.D._i_* is the O.D. at the initial time (*T*_i_) of the exponential phase of the growth curve.

To evaluate the type of co-limitation of the growth response of N and P (simultaneous co-limitation, independent co-limitation, or serial limitation), we followed the log ratio effect-size criteria based on the mean treatment and control response in Harpole et al. (2011) and Hedges et al. (1999). We calculated the following log growth response ratios as follows:

(3) $$\mathrm{Nitrogen}\,\left(N\right)\,\mathrm{response}:\ln\,\left(N_{1}\,P_{0}/N_{0}\,P_{0}\right)$$

(4) $$\mathrm{Phosphorus}\,\left(P\right)\,\mathrm{response}:\ln\,\left(N_{0}\,P_{1}/N_{0}\,P_{0}\right)$$

(5) $$N+P\,\mathrm{response}:\ln\,\left(N_{1}\,P_{1}/N_{0}\,P_{0}\right)$$

For *Halothece* sp., N_0_P_0_ represents the growth rate under extremely limiting NO_3_^–^ conditions at [Low PO_4_^3–^], N_1_P_0_ is the growth rate under [Low NO_3_^–^] or optimal NO_3_^–^ at [Low PO_4_^3–^] conditions, N_0_P_1_ is the growth rate under extremely limiting NO_3_^–^ conditions at [High PO_4_^3–^], and N_1_P_1_ is the growth rate under combinations of under [Low NO_3_^–^] or optimal NO_3_^–^ at [High PO_4_^3–^] conditions. Responses = 0 are responses identical to control values (no response), >0 are positive responses, and <0 are negative responses. The critical threshold value, representing the minimum significant treatment responses at *p* = 0.05 relative to N_0_P_0_, was calculated to graphically determine the nature of co-limitation. To choose this critical threshold value, we used the T-score criteria, in which those values that surpass the critical T-score were considered statistically significant (Supplementary Material, Harpole et al., 2011). We also evaluated the type of co-limitation growth response of PO_4_^3–^ and Fe under NO_3_^–^ optimal and [Low NO_3_^–^] conditions, substituting Fe for N in the eqs. 3–5 above, with Fe_0_P_0_ representing the growth rate under [Low Fe] at [Low PO_4_^3–^] conditions. For *F. muscicola*, we could only evaluate the nature of P and Fe co-limitation because all the treatments were incubated with no added combined N source.

Monod kinetic models were performed with data of the initial concentrations of PO_4_^3–^, Fe, and NO_3_^–^ (Monod, 1949). The Monod growth curves for *Halothece* sp. were plotted against different concentrations of NO_3_^–^ and PO_4_^3–^ incubated at different PO_4_^3–^ ([Low PO_4_^3–^], [Medium PO_4_^3–^], and [High PO_4_^3–^]) and Fe ([Low–Medium Fe] and [High Fe]) concentrations, respectively. The Monod growth curves for *F. muscicola* incubated at different Fe concentrations ([Low–Medium Fe] and [High Fe]) were also plotted against different concentrations of PO_4_^3–^. Monod kinetic parameters were calculated from plotted hyperbolic functions of the Monod equation:

(6) μ⁢=⁢μ⁢m′⁢a⁢x⁢(Q-Q⁢m⁢i⁢n)Q

where *Q* is the concentration of NO_3_^–^ or PO_4_^3–^, *Q*_min_ is the minimal concentration of NO_3_^–^ or PO_4_^3–^ for growth, and μ′*_max_* is the maximum specific growth rate. Half-velocity constant (*K*_μ_) was calculated as the concentration at half of the μ′*_max_* (Monod, 1949).

### Abundance Measurement of *Halothece* sp. Using Flow Cytometry

*Halothece* sp. cells at the end of the different experiments were fixed with glutaraldehyde 25% (v/v) in H_2_O (Sigma-Aldrich) [final concentration 0.05% (v/v)] and were counted in a Becton Dickinson FACSVerse cytometer (Becton and Dickinson, Franklin Lakes, NJ, United States). Fluorescent beads, BD FACSuite^TM^ CS&T research beads (Becton and Dickinson and Company BD Biosciences, San Jose, CA, United States), were used as internal standard to calibrate the instrument. To count *Halothece* sp. cells, we selected fluorescein isothiocyanate (FITC) (488-nm excitation, 530/30-nm emission) and phycoerythrin (PE) (488-nm excitation, 576/26-nm emission) combination fluorescence signals, which show clearly the population of the cells, recording for each sample a total of 10 × 10^3^ cells.

### Pigment Measurement in *Fischerella muscicola*

Chlorophyll (Chla, Chlb, and total Chl) and phycobiliprotein [R-PE and R-phycocyanin (R-PC)] content for *F. muscicola* were measured and calculated following Porra et al. (1989) and Sobiechowska-Sasim et al. (2014). Briefly, for chlorophyll measurements, 5-ml samples of the cultures were filtered through MFV5-025 glass microfiber filters (MFV5-025, FilterLab), grounded, and extracted with 4 ml of acetone 80% for 2 h. After extraction, cells and filter debris were discarded by centrifugation, and the supernatant was read at 647 and 664 nm in quartz cuvette in a Cary Eclipse spectrometer. For phycobiliproteins, 5-ml samples of the culture were filtered as described above, grounded, and extracted in a buffer medium composed of 0.25 M of Trizma base, 10 mM of disodium EDTA, and 2 mg ml^–1^ of lysozyme. After extraction, cells and filter debris were centrifuged, and supernatants were read at 564, 618, and 750 nm in quartz cuvette in a Cary Eclipse spectrometer.

### N_2_-Fixation Rate Measurement by Acetylene Reduction Assay

N_2_-fixation rates of *Halothece* sp. (pmol N_2_-fixed cell^–1^ h^–1^) and *F. muscicola* (nmol N_2_-fixed nmol total Chl^–1^ h^–1^) were measured through acetylene reduction assay (ARA) at the end of the experiments, following the method of Agawin et al. (2014). *Halothece* sp. N_2_-fixing activities were measured under known N_2_-fixing conditions (i.e., anaerobic condition and during the dark photoperiod and in [Low NO_3_^–^]) (Reddy et al., 1993). For *F. muscicola*, measurements of N_2_-fixation rates were performed under aerobic conditions during both light and dark photoperiods. Samples of known volume of the cultures (10 ml) were filtered through 0.45-μm GF/F filters (MFV5-025, FilterLab), these filters were incubated with acetylene at 20% (v/v) final concentration injected in each vial using gas-tight Hamilton syringes, and ethylene production was measured (after 3 h of incubation) by gas chromatographic (GC) methods. Ethylene and acetylene were determined using a GC (model HP-5890, Agilent Technologies) equipped with a flame ionization detector (FID). The column was a Varian wide-bore column (ref. CP7584) packed with CP-PoraPLOT U (27.5-m length, 0.53-mm inside diameter, 0.70-mm outside diameter, 20-μm film thickness). Helium was used as carrier gas at a flow rate of 30 ml min^–1^. Hydrogen and airflow rates were set at 30 and 365 ml min^–1^, respectively. The split flow was used so that the carrier gas flow through the column was 4 ml min^–1^ at a pressure of 5 psi. Oven, injection, and detector temperatures were set at 52, 120, and 170°C, respectively. Before passing the samples in the GC, standard ethylene and acetylene were ran, and their retention times noted. Ethylene produced was calculated using the equations in Stal (1988). The acetylene reduction rates were converted to N_2_-fixation rates (nmol ml^–1^ h^–1^) using a factor of 4:1 (Jensen and Cox, 1983).

### Measurement of Reactive Oxygen Species Production

The ROS production was measured using the molecular probe 2′,7′-dichlorofluorescein diacetate (DCFH-DA; Sigma) in culture media (ASN-III + Turks Island salts 4× or BG11_0_), which was added to a 96-well microplate (Thermo Scientific) containing cyanobacterial samples (final concentration of probe at 15 μg ml^–1^). Within the cells, H_2_O_2_ production oxidizes DCFH-DA to 2′,7′-dichlorofluorescein (DCF) whose green fluorescence can be measured at 25°C in a FLx800 Microplate Fluorescence Reader (BioTek Instruments, Inc.) for 1 h with an excitation of 480 nm and emission of 530 nm. The measurements (the slope of the linear regression obtained) were expressed as arbitrary units (AU) per cell^–1^. DCFH-DA was added in ASN-III + Turks Island salts 4× or BG11_0_ without cells as blanks under the same conditions as above.

### Microscopy and Morphological Studies

Autofluorescence, morphological changes, and DCF oxidized by ROS were observed with a confocal microscope (Leica TCS SPE, Leica Microsystems). Samples were placed onto a clean glass microscope slide, and 40× and 100× objective lens were used to visualize the cells. Images were processed using the software Leica application suite (Leica Microsystems). Autofluorescence was detected with an excitation of 532 nm and an emission of 555- to 619-nm wavelengths. For visualization of DCF oxidized by ROS, we used excitation of 488 nm and emission of 493- to 562-nm wavelengths. Cell apoptosis for *F. muscicola* was observed with Alexa Fluor 488 annexin V/Dead Cell Apoptosis Kit (Thermo Fisher), in which apoptotic cells were detected when the exposed phosphatidylserine of these cells conjugates with annexin. Annexin was detected and visualized at excitation of 530–570 nm and emission of 488-nm wavelengths.

### Bio-Informatic *in silico* Analysis

The TBSs (PHO box, Fur, and NtcA boxes) for SphR (i.e., PhoB), ferric uptake regulator (Fur), and global nitrogen regulator (NtcA) already described in cyanobacteria were explored and downloaded from RegPrecise (May, 2020), a web resource for collection, visualization, and analysis of transcriptional regulons reconstructed by comparative genomics (Novichkov et al., 2013). From this database, multifasta files were generated for each TBS, including several genera of cyanobacteria (*Cyanothece*, *Gloeobacter*, *Microcystis*, *Nostoc*, *Prochlorococcus*, *Synechococcus*, *Synechocystis*, *Thermosynechococcus*, and *Trichodesmium*). We obtained three separated multifasta files (one for each TBS type) that contained 77 sequences for PHO box, 167 sequences for Fur boxes, and 228 sequences for NtcA boxes (Supplementary Material). For the prediction of the TBS (PHO box, Fur, and NtcA boxes) for *Halothece* sp., we used a user-friendly web interface for prediction of prokaryote promoter elements and regulons, PePPER, using the DNA motif build and search platform^1^ (de Jong et al., 2012). Position frequency matrices (PFMs; using MEME and subsequently transposed to a MOODS compatible PWM format) were made from each multifasta file (Supplementary Material). The PFMs generated were searched in the whole genome of *Halothece* sp. PCC 7418 (GenkBank: NC_019779.1), obtaining 804 hits for SphR (PhoB), 822 hits for Fur, and 238 hits for NtcA. The corresponding hits were manually explored with UGENE (Golosova et al., 2014). We selected the potential TBS on the basis of their relative positions to the genes (a maximum of −800 bp upstream or +40 bp downstream). DNA sequence logos were generated with WebLogo (Crooks et al., 2004) to graphically indicate the relative frequency of each amino at each position, i.e., predicting the consensus sequences for Pho, Fur, and NtcA boxes. Finally, the genes detected as associated to PHO box, Fur, and NtcA boxes were annotated and submitted to EggNOG v5.0 for further identification of orthology relationships and for functional analysis to describe the Pho, Fur, and NtcA regulons, respectively (Huerta-Cepas et al., 2018).

### Statistical Analysis

Non-parametric test, Kruskal–Wallis analysis followed by an unpaired two-samples Wilcoxon test was used to study the effect of the nutrient treatment conditions to growth, pigment concentrations, N_2_-fixation rates, and ROS production. Spearman’s correlation was used to determine the relationships between growth and N_2_-fixation rates. The statistical analyses were performed using the SPSS program version 21 (IBM Corp year 2012).

## Results

### Effect of Varying Nutrient Concentrations (P, Fe, and/or N) on *Halothece* sp. and *Fischerella muscicola*

#### Growth of *Halothece* sp. Under Different Concentrations of NO_3_^–^

The decreasing concentrations of inorganic nitrogen (i.e., NO_3_^–^) negatively affected the growth of *Halothece* sp. PCC 7418 (Kruskal–Wallis, *H* = 24.55, 2 d.f., *p* = 0.045, Table 1). Average growth rates (μ, of all the combinations of different PO_4_^3–^ and Fe concentrations) under optimal, low, and extremely limiting NO_3_^–^ conditions were 0.33 ± 0.017 day^–1^ (*T*_g_, 2.18 days), 0.24 ± 0.023 day^–1^ (*T*_g_, 3.37 days), and 0.046 ± 0.0075 day^–1^ (*T*_g_, 16.52 days), respectively. Under extremely limiting NO_3_^–^ conditions, the growth of the unicellular cyanobacteria dramatically decreased with rates eight times lower than in optimal NO_3_^–^ conditions, and cells were not able to overcome the latent growth phase in the cultures (Table 1).

**TABLE 1:** Growth rate (μ) and generation time (*T*_g_) for *Halothece* sp. (at different NO_3_^–^ concentrations) and *Fischerella muscicola* at different combinations of PO_4_^3–^ and Fe concentrations.

**Treatments/** **parameters**	***Halothece* sp.**						***Fischerella muscicola***	
	
	**Optimal NO_3_^–^ (4.4 mM)**		**Low NO_3_^–^ (0.15 mM)**		**Under extremely limiting** **NO_3_^–^ (6.66 nM)**		**N_2_ as only source of N**	
	
	**μ ± SR (d^–1^)**	***T*_g_ ± SR (d)**	**μ ± SR (d^–1^)**	***T*_g_ ± SR (d)**	**μ ± SR (d^–1^)**	***T*_g_ ± SR (d)**	**μ ± SR (d^–1^)**	***T*_g_ ± SR (d)**
[Low PO_4_^3–^- Low Fe]	0.31 ± 0.06	2.33 ± 0.01	0.19 ± 0.01	3.74 ± 0.26	0.05 ± 0.00	12.91 ± 0.77	0.30 ± 0.03	2.32 ± 0.22
[Low PO_4_^3–^- Medium Fe]	0.31 ± 0.06	2.36 ± 0.02	0.13 ± 0.02	5.29 ± 0.65	*	*	0.26 ± 0.06	2.81 ± 0.62
[Low PO_4_^3–^- High Fe]	0.35 ± 0.08	2.08 ± 0.01	0.13 ± 0.01	5.56 ± 0.65	0.03 ± 0.00	20.87 ± 0.00	0.43 ± 0.26	2.53 ± 0.88
[Medium PO_4_^3–^- Low Fe]	0.33 ± 0.09	2.24 ± 0.03	0.25 ± 0.03	2.82 ± 0.30	*	*	0.42 ± 0.12	1.79 ± 0.52
[Medium PO_4_^3–^- Medium Fe]	0.27 ± 0.01	2.60 ± 0.05	0.29 ± 0.05	2.49 ± 0.41	*	*	0.22 ± 0.04	3.28 ± 0.58
[Medium PO_4_^3–^- High Fe]	0.42 ± 0.00	1.63 ± 0.03	0.20 ± 0.03	3.47 ± 0.53	*	*	0.25 ± 0.10	3.25 ± 1.29
[High PO_4_^3–^- Low Fe]	0.28 ± 0.05	2.56 ± 0.02	0.40 ± 0.02	1.75 ± 0.10	0.07 ± 0.02	10.84 ± 2.84	0.45 ± 0.23	2.06 ± 1.05
[High PO_4_^3–^- Medium Fe]	0.35 ± 0.04	2.04 ± 0.01	0.39 ± 0.01	1.77 ± 0.04	*	*	0.50 ± 0.09	1.44 ± 0.25
[High PO_4_^3–^- High Fe]	0.34 ± 0.03	2.04 ± 0.03	0.20 ± 0.03	3.54 ± 0.54	0.03 ± 0.01	21.36 ± 3.49	0.74 ± 0.00	0.94 ± 0.00

Values are the mean with their spanning range (SR) between the duplicate measurements. Asterisks (*) are conditions in which μ and *T*_g_ were not measured.

#### Growth of *Halothece* sp. Under Different PO_4_^3–^ and Fe Concentrations

**FIGURE 1:**
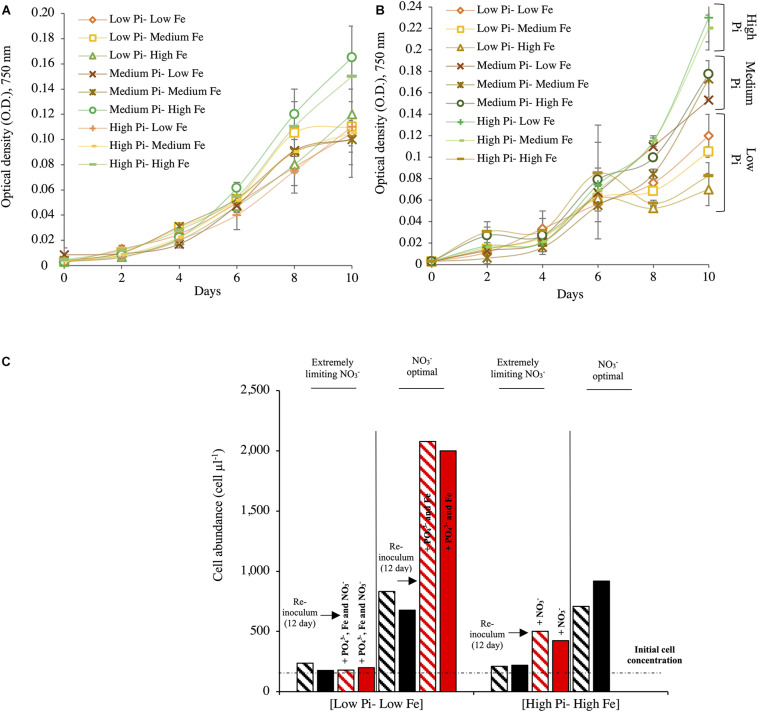
Growth of *Halothece* sp. at different combinations of nutrient concentrations (PO_4_^3–^, Fe, and NO_3_^–^). **(A)** Growth for *Halothece* sp. under NO_3_^–^ optimal conditions. **(B)** Growth for *Halothece* sp. under [Low NO_3_^–^] conditions. **(C)** Cell concentration after 12 days of incubation and recovery of *Halothece* sp. under different NO_3_^–^ concentrations with the re-inoculum (black arrow) of PO_4_^3–^, Fe, and/or NO_3_^–^ and incubated 4 days more (red bars). The initial cell concentration is referenced with the black dashed line. PO_4_^3–^ is represented as Pi. In panels **(A,B)**, values are the mean with their spanning range (SR) between the duplicate measurements. In panel **(C)**, data presented are actual values of the duplicates (striped and solid bars).

No significant differences in growth of *Halothece* sp. were observed among different combination conditions of PO_4_^3–^ and Fe under optimal NO_3_^–^ (Kruskal–Wallis, *H* = 7.21, 8 d.f., *p* = 0.51, Table 1; and *H* = 9.02, 8 d.f., *p* = 0.34, Figure 1A). On the contrary, under [Low NO_3_^–^] conditions, significant differences were found (Kruskal–Wallis, *H* = 15.83, 8 d.f., *p* = 0.045, Figure 1B). Under [Low NO_3_^–^], at high concentrations of PO_4_^3–^, μ was generally higher than at medium and low concentrations (Table 1 and Figure 1B) except when it was combined with high Fe levels (Table 1 and Figure 1B), showing that high Fe levels had a negative impact on their growth at [High PO_4_^3–^] (Figure 1B).

#### Recovery of *Halothece* sp. After Nutrient Re-inoculation

The recovery of *Halothece* sp. cells from low PO_4_^3–^ and low Fe (and/or under extremely limiting NO_3_^–^) to high PO_4_^3–^ and high Fe concentrations with re-inoculation of the nutrients (PO_4_^3–^, Fe, and/or NO_3_^–^) in the medium was dependent on the initial conditions of PO_4_^3–^, Fe, and NO_3_^–^ concentrations in the culture medium (Figure 1C). In the [Low PO_4_^3–^–Low Fe] condition under extremely limiting NO_3_^–^ conditions, the cells exhibited total chlorosis, and the addition of PO_4_^3–^, Fe, and NO_3_^–^ (at final concentrations of 45 μM, 7.5 μM, and 4.4 mM, respectively) was not sufficient to restore their original phenotype. However, in [High PO_4_^3–^–High Fe] treatments under extremely limiting NO_3_^–^ conditions, re-inoculation of NO_3_^–^ exhibited slight recovery (recovering their greenish color; data not shown). In addition, in [Low PO_4_^3–^–Low Fe] treatment under optimal NO_3_^–^, which was re-inoculated with PO_4_^3–^ and Fe, the cells responded with higher growth and recovered their greenish color (Figure 1C).

#### Growth of *Fischerella muscicola* Under Different PO_4_^3–^ and Fe Concentrations

**FIGURE 2:**
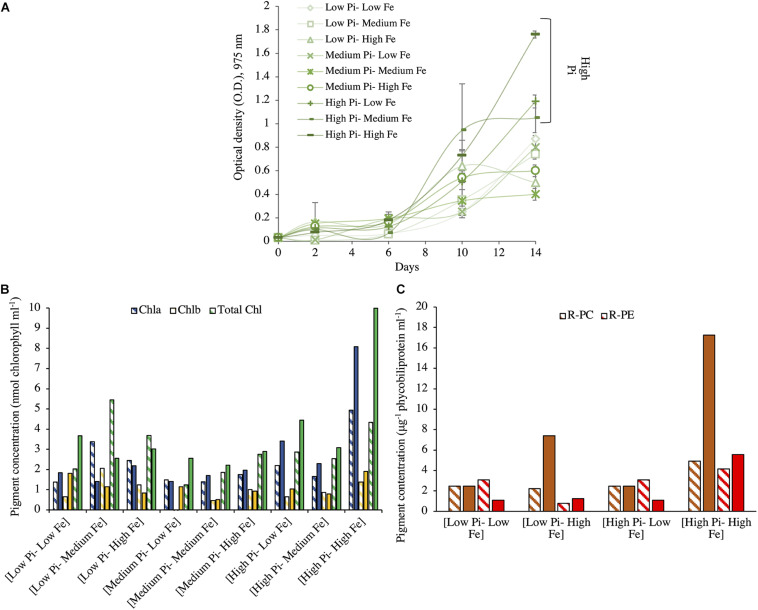
Growth and pigment content for *Fischerella muscicola* at different concentrations of PO_4_^3–^ and Fe after 14 days of incubation. **(A)** Growth for *F. muscicola* at different combinations of PO_4_^3–^ and Fe where values are the mean with their spanning range (SR) between the duplicate measurements. **(B)** Chlorophyll (Chla, Chlb, and total Chl) and **(C)** phycobiliproteins (R-PC and R-PE) concentrations at different combinations of PO_4_^3–^ and Fe concentrations. PO_4_^3–^ is represented as Pi. In panels **(B,C)**, data presented are actual values of the duplicates (striped and solid bars).

Unlike what was observed for *Halothece* sp., decreasing concentration of NO_3_^–^ did not affect the μ of *F. muscicola* PCC 73103 (data not shown). With N_2_ as sole N source, *F. muscicola* reached the maximum μ of 0.74 day^–1^ (*T*_g_, 0.94 days) at the highest concentrations of PO_4_^3–^ and Fe, and it was higher than the rest of the treatments (Kruskal–Wallis, *H* = 16.03, 8 d.f., *p* = 0.042, Figure 2A). The cells under [High PO_4_^3–^–High Fe] conditions also showed the highest average content of Chla, Chlb, and total chlorophyll (total Chl) (Figure 2B) and phycobiliprotein (R-PC and R-PE) content (Figure 2C).

#### Nature of Limitation of PO_4_^3–^, Fe, and/or NO_3_^–^ in *Halothece* sp. and/or *Fischerella muscicola*

**FIGURE 3:**
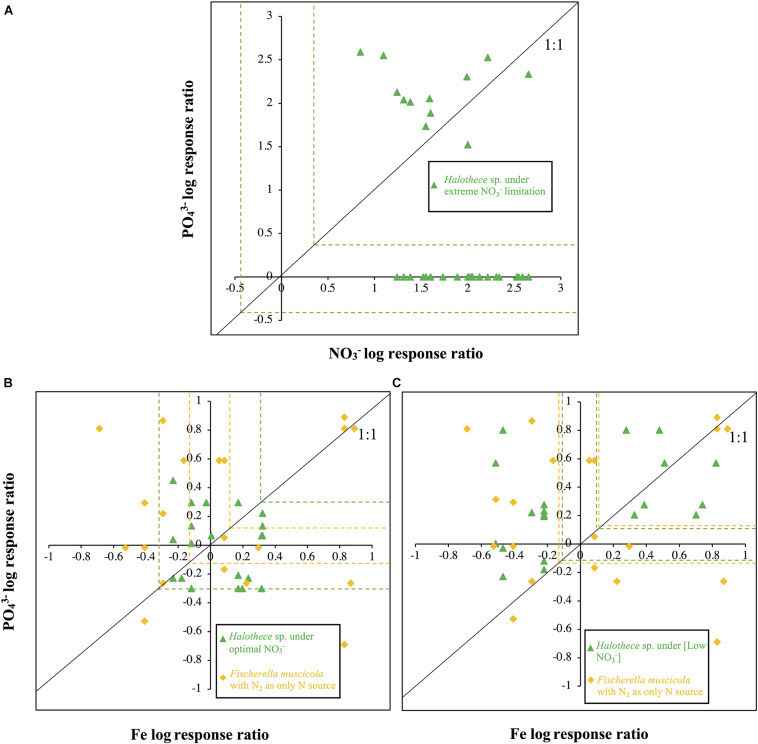
Log nutrient responses for *Halothece* sp. and *Fischerella muscicola*. **(A)** PO_4_^3–^ and NO_3_^–^ log response ratios for *Halothece* sp. **(B)** and **(C)** PO_4_^3–^ and Fe log response ratios for *Halothece* sp. **(B)** under optimal NO_3_^–^ and **(C)** under [Low NO_3_^–^] conditions and *F. muscicola* with N_2_ as sole N source. Each data point represents each replicate in which *X*-axis is the NO_3_^–^ or Fe log response and *Y*-axis is the PO_4_^3–^ log response. Dashed green (for *Halothece* sp.) and yellow lines (for *F. muscicola*) represent the critical threshold values [data points outside the critical values are significantly different (at *p* = 0.05) from N_0_P_0_ or Fe_0_P_0_]. PO_4_^3–^ is represented as Pi.

The evaluation of the limitation of NO_3_^–^ and PO_4_^3–^ in *Halothece* sp. under extremely limiting NO_3_^–^ conditions concentrations revealed that cells were serially limited, i.e., first with NO_3_^–^ and then with PO_4_^3–^ (N response > 0, P response = 0 and N + P response > 0) (Figure 3A). PO_4_^3–^ did not have any effect on the growth of cells when they were extremely N limited. Evaluating the effects of the addition of PO_4_^3–^ and Fe with increased N supply, we showed that under optimal NO_3_^–^, *Halothece* sp. did not respond significantly to any addition of PO_4_^3–^ and Fe (*p* < 0.05, Figure 3B). Under sufficient N supply (i.e., at [Low NO_3_^–^] when maximum rates of N_2_-fixation were measured), PO_4_^3–^ was the main limiting nutrient and Fe had a negative effect on the growth of *Halothece* sp. at high Fe levels (Fe response < 0, P response > 0, and P + Fe response > 0) (Figure 3C). However, we also found a simultaneous co-limitation of PO_4_^3–^ and Fe (Fe response = 0, P response = 0, and P + Fe response > 0) when both elements were added at medium PO_4_^3–^ and medium–high Fe concentrations for *Halothece* sp. (Figure 3C). For *F. muscicola*, a simultaneous co-limitation of PO_4_^3–^ and Fe was also observed (Fe response = 0, P response = 0, and P + Fe response > 0) when both elements were added at high PO_4_^3–^ and Fe concentrations (Figure 3C).

#### Monod Growth Kinetics of *Halothece* sp. and *Fischerella muscicola*

**FIGURE 4:**
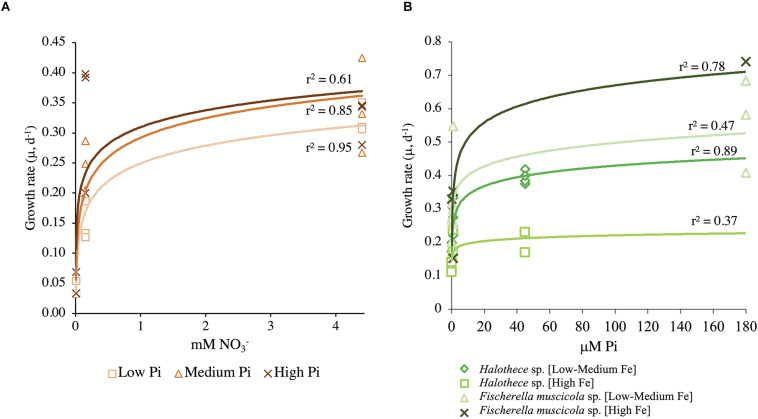
Monod curves **(A)** at different PO_4_^3–^ concentrations for *Halothece* sp. and *Fischerella muscicola* in function of NO_3_^–^ initial levels and **(B)** at different Fe concentrations for *Halothece* sp. (under [Low NO_3_^–^]) and *F. muscicola* in function of PO_4_^3–^ initial levels. PO_4_^3–^ is represented as Pi.

The N-dependent Monod growth kinetics of *Halothece* sp. incubated at different PO_4_^3–^ concentrations showed P dependence (Figure 4A). *Halothece* sp. had higher maximum growth at high and medium PO_4_^3–^ levels (μ′*_max_* = 0.50 day^–1^) compared with low PO_4_^3–^ levels (μ′*_max_* = 0.44 day^–1^). However, the half-velocity constant at [High PO_4_^3–^] (*K*_μ_ = 0.217 mM, with a *Q*_min_ = 1.55 mM) was the lowest compared with that at [Medium PO_4_^3–^] (*K*_μ_ = 0.45 mM, with a *Q*_min_ = 1.19 mM) and [Low PO_4_^3–^] (*K*_μ_ = 0.53 mM, with a *Q*_min_ = 1.22 mM) (Figure 4A). The P-dependent growth kinetics of *Halothece* sp. incubated at different Fe concentrations showed μ′*_max_* (0.25 day^–1^) and *Q*_min_ (5.37 μM) at high Fe levels that were lower and higher, respectively, than at low to medium Fe levels (μ′*_max_* = 0.43 day^–1^, *Q*_min_ = 3.13 μM); but *K*_μ_ was lower at [High Fe] (*K*_μ_ = 0.01 μM) than in [Low–Medium Fe] (*K*_μ_ = 0.35 μM) (Figure 4B). On the other hand, *F. muscicola* generally had higher values of the P-dependent kinetic parameters than *Halothece* sp. when incubated at different Fe concentrations (Figure 4B). *F. muscicola* had the higher maximum growth (μ′*_max_* = 0.83 day^–1^), half-velocity constant (*K*_μ_ = 1.85 μM), and higher minimal cell quota (*Q*_min_ = 12.58 μM) at high Fe treatments as compared with cells at low to medium levels of Fe (μ′*_max_* = 0.62 day^–1^, *K*_μ_ = 0.03 μM, *Q*_min_ = 12.33 μM) (Figure 4B).

### Effect of Varying Concentrations of P and Fe on N_2_-Fixation Rates of *Halothece* sp. and *Fischerella muscicola*

**FIGURE 5:**
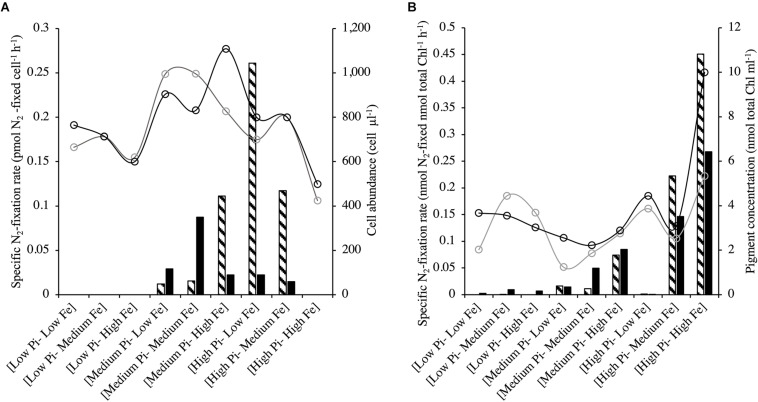
N_2_-fixation rates for **(A)** *Halothece* sp. and **(B)** *Fischerella muscicola* at different combinations of PO_4_^3–^ and Fe concentrations. *Halothece* sp. were grown under [Low NO_3_^–^] to promote N_2_-fixation. Bar graphics indicate N_2_-fixation, while empty circles indicate cell abundance (cell μl^–1^) for *Halothece* sp. and chlorophyll concentrations (nmol total Chl ml^–1^) for *F. muscicola*. Data presented are actual values of the duplicates (striped and solid bars or light and heavy lined circles). PO_4_^3–^ is represented as Pi.

*Halothece* sp.-specific N_2_-fixation rates under [Low NO_3_^–^] at different treatment combinations of PO_4_^3–^ and Fe were significantly linearly correlated with cell concentrations (Spearman’s correlation, *r* = 0.7, *p* < 0.05, *n* = 18). N_2_-fixation rates at [Low PO_4_^3–^] conditions were undetectable and generally increased at medium PO_4_^3–^ and at increasing Fe concentrations (Figure 5A). However, at the highest PO_4_^3–^ and Fe concentrations, N_2_-fixation was again undetectable (Figure 5A). For *F. muscicola*, where specific N_2_-fixation rates were measured during the light and dark photoperiods, there were no significant differences among the treatments during the light and dark phase (Kruskal–Wallis, *H* = 15.45, 8 d.f., *p* = 0.051; data not shown). Higher N_2_-fixation rates under high PO_4_^3–^ levels were detected, especially when combined with high Fe concentrations (Figure 5B).

### Effect of Varying Concentrations of P and Fe on the Production of Reactive Oxygen Species of *Halothece* sp. and *Fischerella muscicola*

**FIGURE 6:**
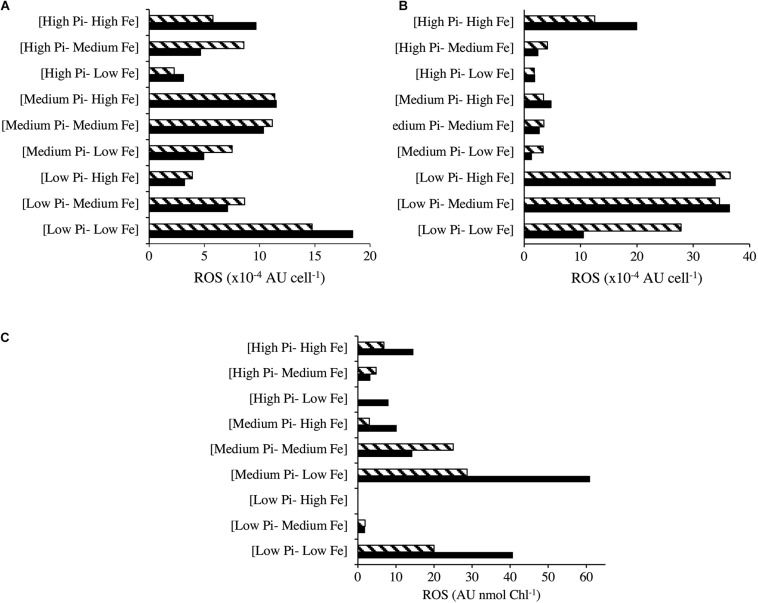
Radical oxygen species (ROS) production at different combinations of PO_4_^3–^ and Fe concentrations for *Halothece* sp. under **(A)** NO_3_^–^ optimal and **(B)** [Low NO_3_^–^] conditions and **(C)** for *F. muscicola*. PO_4_^3–^ is represented as Pi. Data presented are actual values of the duplicates (striped and solid bars).

**FIGURE 7:**
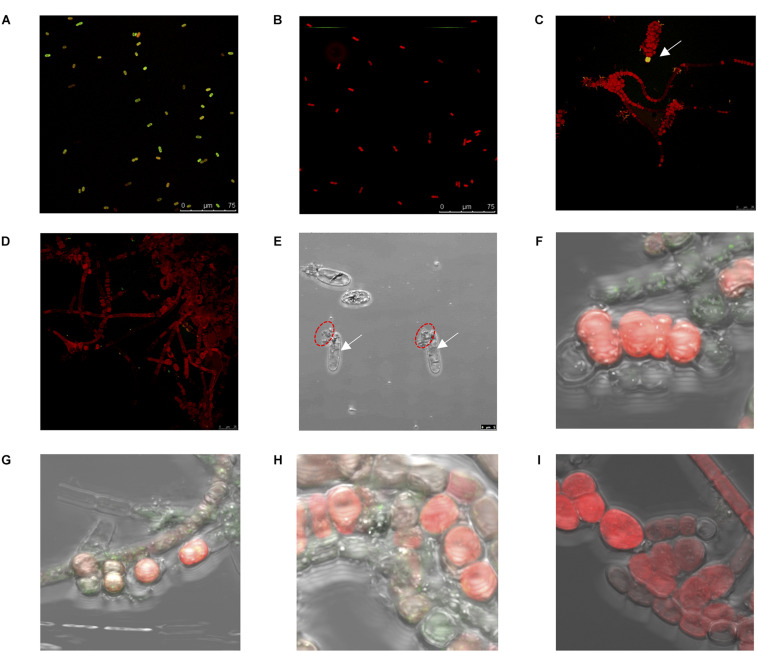
Biochemical [e.g., reactive oxygen species (ROS) production], morphological, and physiological changes for *Halothece* sp. and *Fischerella muscicola*. **(A–D)** Confocal images of ROS production (green-yellow color) for *Halothece* sp. at panel **(A)** [Low PO_4_^3–^–Low Fe] and panel **(B)** [High PO_4_^3–^–High Fe] conditions and for *F. muscicola* at panel **(C)** [Low PO_4_^3–^–Low Fe] (in which the white arrow indicates the ROS production) and panel **(D)** [High PO_4_^3–^–High Fe] conditions. Images were taken at 400×. Panel **(E)** [Low PO_4_^3–^–Low Fe] condition under extremely limiting NO_3_^–^ conditions under the bright field (arrows indicate vacuole formation, and red dashed circles indicate cell breaks). **(F–I)** Apoptosis assay results for *F. muscicola* under confocal microscopy. Panel **(F)** [Low PO_4_^3–^–Low Fe], panel **(G)** [Low PO_4_^3–^–High Fe], panel **(H)** [High PO_4_^3–^–Low Fe], and panel **(I)** [High PO_4_^3–^–High Fe]. In panels **(F–I)**, the green color resulted from the binding of annexin with the phosphatidylserines that were exposed in apoptotic cells, and images **(E–I)** were taken at 1,000× with zoom. PO_4_^3–^ is represented as Pi.

Reactive oxygen species production for *Halothece* sp. was measured under optimal NO_3_^–^ and [Low NO_3_^–^] conditions (Figures 6A,B). Under optimal NO_3_^–^, there were significant differences among the treatments (Kruskal–Wallis, *H* = 15.67, 8 d.f., *p* = 0.047, Figure 6A). ROS production was higher at [Low PO_4_^3–^–Low Fe] conditions compared with increasing concentrations of PO_4_^3–^ at the same [Low Fe] conditions (Figure 6A). At [Low PO_4_^3–^] conditions, increasing Fe concentration reduced the ROS production (Figure 6A). On the other hand, cells under PO_4_^3–^ and Fe limitation ([Low PO_4_^3–^–Low Fe]) and optimal NO_3_^–^ showed higher ROS production, as observed with the greener color (oxidized DCF) of cells, compared with the red color of cells that were not producing ROS in [High PO_4_^3–^–High Fe] (Figures 7A,B). On the other hand, under [Low NO_3_^–^] conditions, we did not detect significant differences in ROS production (Kruskal–Wallis, *H* = 13.80, 8 d.f., *p* = 0.087, Figure 6B). Nonetheless, we observed ROS production under [Low NO_3_^–^] conditions to be higher at [Low PO_4_^3–^–Medium/High Fe] than the rest of the treatments except at [High PO_4_^3–^–High Fe] and [Low PO_4_^3–^–Low Fe] (Figure 6B). At [Medium Fe] conditions, increasing PO_4_^3–^ concentrations reduced ROS production (Figure 6B).

The ROS production of *F. muscicola* did not vary among the different treatment combinations of PO_4_^3–^ and Fe (Kruskal–Wallis, *H* = 12.30, 8 d.f., *p* = 0.14, Figure 6C). Nonetheless, there was a tendency of higher ROS production at [Low PO_4_^3–^–Low Fe] compared with [High PO_4_^3–^–High Fe] conditions, consistent with the confocal images showing oxidative stress, observing more green-yellowish color cells (indicative of ROS production) in [Low PO_4_^3–^–Low Fe] treatments (Figure 7C) than in [High PO_4_^3–^–High Fe] conditions (Figure 7D).

### Effect of Varying Concentrations of P and Fe on the Morphology and Physiology of *Halothece* sp. and *Fischerella muscicola*

*Halothece* sp. cells under optimal nutrient conditions (PO_4_^3–^, Fe, and/or NO_3_^–^) exhibited sizes between 4 and 7 μm. Limitation of PO_4_^3–^ and Fe under NO_3_^–^ optimal and [Low NO_3_^–^] conditions did not cause changes in sizes. However, extremely limiting NO_3_^–^ conditions caused an increase in size of the cells (up to two-fold with respect to the normal size), reaching an average size of 12.7 ± 0.74 μm. Under extremely limiting NO_3_^–^ conditions together with PO_4_^3–^ and Fe limitation, cells experience vacuole production together with modification of shape and breakage of the cells (Figure 7E). In addition, we also observed that under these same conditions, cells experience total chlorosis (dramatic loss of phycobiliproteins), and cells were barely visible under confocal microscopy compared with cells under optimal nutrient conditions.

Programmed cell death for *F. muscicola* was studied through apoptosis assay to detect mortality in four treatments: [Low PO_4_^3–^–Low Fe] (Figure 7F), [Low PO_4_^3–^–High Fe] (Figure 7G), [High PO_4_^3–^–Low Fe] (Figure 7H), and [High PO_4_^3–^–High Fe] (Figure 7I). Results showed that PO_4_^3–^ and Fe limitation caused increased mortality. At [High PO_4_^3–^–High Fe], there was no signal of annexin V in conjugation with phosphatidylserine detected, indicating the good status of the cells in this treatment.

### Prediction of Genes Involved in P, Fe, and N Adaptation and Survival in *Halothece* sp.

**FIGURE 8:**
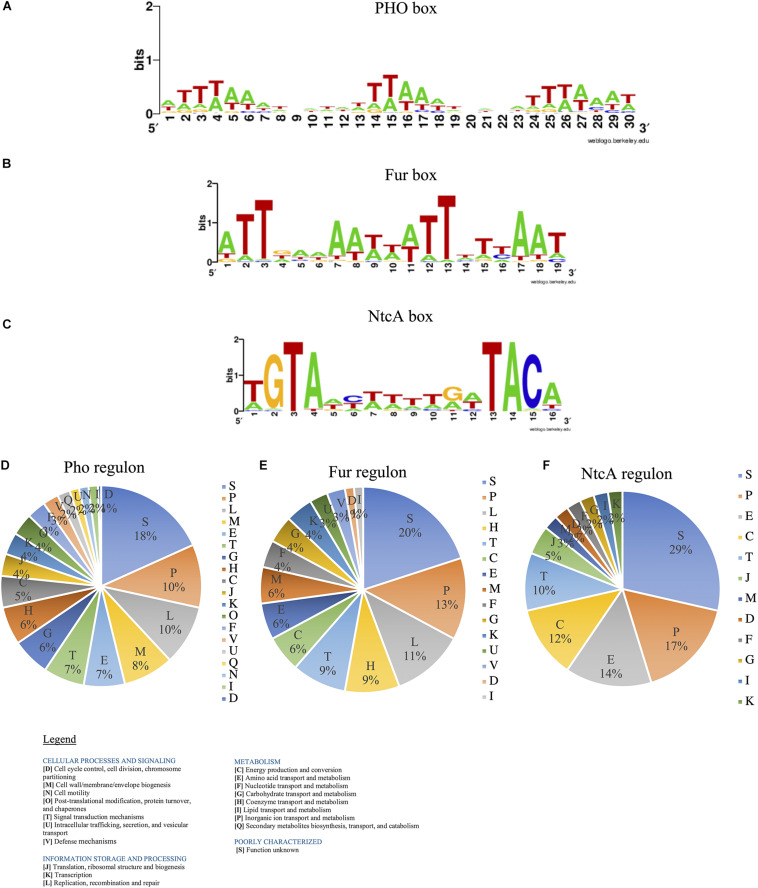
Transcriptional binding sites (TBS) for PhoB, Fur, and NtcA regulators predicted for *Halothece* sp. **(A)** PHO box, **(B)** Fur box, and **(C)** NtcA box in *Halothece* sp. The Markov models were built with WebLogo. **(D–F)** Single-letter functional codes (COGs) for **(D)** Pho regulon, **(E)** Fur regulon, and **(F)** NtcA regulon in *Halothece* sp. PO_4_^3–^ is represented as Pi.

We predicted the consensus sequences for TBSs for SphR-PhoB (PHO box), Fur regulator (Fur Box), and NtcA protein (NtcA box) for *Halothece* sp. For PHO box, we established that consensus sequence was formed by three tandem repeats of 8 bp ([ATTTAAAT]_3_) separated by 3 bp (Figure 8A). Fur Box was formed by 19 bp inverted repeats (ATTGAAAATTATTTT[T/C]AAT) (Figure 8B). Finally, NtcA box was constituted by TGTAN_8_TACA in which GTA at positions 2–4 and TAC at positions 13–15 were well conserved (Figure 8C). From the prediction of these boxes, we described what potential genes are implicated in Pho, Fur, and NtcA regulons (Supplementary Tables S2–S4).

Pho regulon (Figure 8D) was mainly constituted by genes involved in inorganic ion transport and metabolism (P) (10%), replication, recombination and repair (L) (10%), and cell wall/membrane/envelope biogenesis (M) (8%), detecting 218 genes (Supplementary Table S2). Among them, we detected genes that are part of the “classical” Pho regulon (e.g., some of them found in the same operon): PstS family phosphate ABC transporter substrate-binding protein-phosphate (*pstS*), ABC transporter permease subunit PstC-phosphate (*pstC*), ABC transporter permease PstA-phosphate (*pstA*), ABC transporter ATP-binding protein PstB (*pstB*), PhoQ sensor (*phoQ*), or alkaline phosphatase (*phoA*). In addition, we detected other genes under the control of PhoB such as *nblA* (involved in chlorosis processes); *gvpA*, *gvpN*, *gvpL*, and *gvpF* (involved in gas vesicles); *cas* genes (involved in CRISPR-Cas systems); and a whole group of genes that codify for transferases, endonucleases, ABC transporters, or transposases (Supplementary Table S2).

Fur regulon (Figure 8E) mostly was distributed in P (13%), L (11%), coenzyme transport and metabolism (H) (9%), and signal transduction mechanisms (T) (9%), detecting 73 genes (Supplementary Table S3). We detected genes under the control of Fur box directly involved in Fe acquisition, such as energy transducer TonB (*tonB*), MotA/TolQ/ExbB proton channel family protein, and three operons of transporter permease subunit (*feoA*–*feoB*). Fur boxes were detected upstream of genes related in photosynthesis and respiration [e.g., photosystem II reaction center protein X (*psbX*)], in the pathway of chlorophyll biosynthesis [glutamate-1-semialdehyde-2,1-aminomutase (*gsaI*)], and in bacteriochlorophyll biosynthesis [magnesium-protoporphyrin IX monomethyl ester anaerobic oxidative cyclase/DNA-binding response regulator (*bchE*)].

Finally, NtcA regulon (Figure 8F) was constituted by genes mostly involved in P (17%), amino acid transport and metabolism (E) (14%), and energy production and conversion (C) (12%), detecting 40 genes (Supplementary Table S4). We detected genes involved in N_2_-fixation processes: FeMo cofactor biosynthesis protein (*nifB*) and molybdate ABC transporter substrate-binding protein (*modA*); in nitrogen assimilation: ferredoxin–nitrite reductase (*nirA*); in glutamine/glutamate assimilation: glutamate synthase [NADPH] large chain (*gltB*), type I glutamate-ammonia ligase (*glnN*) and sodium:glutamate symporter (*gltS*); and in ammonium assimilation: ammonium transporter (*amt*). In addition, Fur regulator itself potentially has a NtcA box.

## Discussion

### Effect of Varying N Concentrations on *Halothece* sp. Compared With *Fischerella muscicola*

In this study, the dependence on the concentration of combined N sources such as NO_3_^–^ for growth of diazotrophic cyanobacterial cultures differed between the unicellular *Halothece* sp. and the filamentous heterocyst-forming *F. muscicola* (Table 1 and Figures 1A–C, 2A, 3A, 4A). Theoretically, N_2_-fixers are independent of inorganic N source (e.g., *F. muscicola*) because of their capability of fixing N_2_ (Knapp, 2012). However, at extremely limiting NO_3_^–^ conditions (i.e., 6.66 nM), almost having N_2_ as sole N source, *Halothece* sp. were barely growing (Table 1 and Figures 1C, 4A), and their morphology (increased size) and physiology (i.e., exhibiting extreme chlorosis) changed. Chlorosis can result from the deprivation of NO_3_^–^, which promotes the degradation of phycobilisomes through the protein NblA (Klotz et al., 2015), which was detected here under the PhoB control (Supplementary Table S2). The observed increases in cell size, possibly, is a result of cell division cessation (Klotz et al., 2016). Moreover, vacuole formation was observed possibly due to the storage of compounds in response to severe N limitation (Figure 7E). Our results indicate that in some N_2_-fixing species, e.g., *Halothece* sp., a minimum amount of combined N sources is necessary for the proper functioning of the cells (*Q*_min_ ranging from 1.19 to 1.51 mM of NO_3_^–^, Figure 4A). However, it remains to be investigated whether these cells in the natural environment, indeed, require a combined source of inorganic nitrogen and if this requirement can be supplied by other diazotrophs in N-limited waters.

Contrary with *Halothece* sp., *F. muscicola* cells were totally independent of combined N source and were able to grow with N_2_ as the sole N source. The viability of unicellular N_2_-fixers to grow under N_2_ as sole N source may be dependent on the presence and type of additional carbon (C) source to support their growth as shown with *Synechococcus* sp. strain SF1 (isolated from macroalgae, *Sargassum fluitans*), which was not able to grow with HCO_3_^–^ as sole C source (Spiller and Shanmugam, 1987). Here, *Halothece* sp. were cultured with glucose and citrate, and these may not be the optimal C source for their growth when grown at almost having N_2_ as sole N source. Unicellular N_2_-fixers also have been shown to grow more when NO_3_^–^ is added compared when grown with only N_2_ as sole N source (Agawin et al., 2007), since a larger energetic cost is associated with assimilating N_2_ versus NO_3_^–^ assimilation. Since N_2_-fixation is sensitive to oxygen (O_2_), cyanobacteria must develop strategies to avoid N_2_-fixation inhibition by the O_2_ liberated from photosynthesis. Unicellular cyanobacterial cells are able to separate temporarily two incompatible processes: N_2_-fixation (at night) and photosynthesis (at day) in the same cell (De Bruijn, 2015). These two processes have to be tightly controlled to avoid inhibition of N_2_-fixation through well-regulated circadian clocks (Toepel et al., 2008), and these strategies can be energy consuming. Aside therefore of the energy cost of assimilating N_2_, *Halothece* sp. may have an add-on cost of temporally separating N_2_-fixation and photosynthetic process, and thus they cannot be totally independent of combined sources of inorganic N. On the contrary, filamentous heterocyst-forming cells have specialized cells (heterocyst) in which N_2_-fixation takes place in anoxic conditions and may not have an add-on cost of temporally separating N_2_-fixation and photosynthetic processes and thus are better adapted to growing with N_2_ as sole N source (De Bruijn, 2015; Figure 5B).

### Effect of Varying Concentrations of P and Fe on *Halothece* sp. and *Fischerella muscicola*

#### Concentration of P and Fe in *Halothece* sp. and *Fischerella muscicola*

Phosphorus (P) is needed for many cellular components, such as cellular membranes, nucleic acids, and ATP-dependent reactions such as N_2_-fixation, which is energetically costly, requiring 16 ATPs (N_2_ + 8e^–^ + 16ATP + 8H^+^ → 2NH_3_ + H_2_ + 16ADP + 16PO_4_^3–^) (Hoffman et al., 2014). Under optimum conditions for N_2_-fixation (at low [Low NO_3_^–^] for *Halothece* sp. and without any combined N source in the case of *F. muscicola*), increasing concentrations of PO_4_^3–^ generally increased the growth of both species, suggesting that PO_4_^3–^ was an important limiting factor for both species and affected their N_2_-fixation rates (Figures 1–5). These results are consistent with the previous study of Fernández-Juárez et al. (2019) reporting the increased rates of P-acquisition mechanisms (PO_4_^3–^-uptake rates and APAs) at [Low NO_3_^–^] for *Halothece* sp. At high concentrations of NO_3_^–^ where N_2_-fixation rates were lower (and thus this P-requirement can be lower), growth of *Halothece* sp. was independent of varying concentrations of PO_4_^3–^. It is well described that in some cyanobacteria, e.g., *Trichodesmium* sp., one of the major N_2_-fixers in the Atlantic, P is the main element regulating N_2_-fixation (Sañudo-Wilhelmy et al., 2001). Under P-limiting environments (e.g., the Mediterranean Sea), N_2_-fixers must therefore depend on external P sources (such as Saharan dust deposition, Tanhua et al., 2013), and cells have to be well adapted to P limitation with mechanisms for phosphorus scavenging (Dyhrman and Haley, 2006; Fernández-Juárez et al., 2019).

Iron (Fe) had differing effects for *Halothece* sp. (i.e., toxic effect under high Fe levels under [Low NO_3_^–^]) and for *F. muscicola* (i.e., limiting growth under low Fe levels) (Table 1 and Figures 1B, 2A, 3B,C, 4, 5). The results for *F. muscicola* were consistent with studies on *Crocosphaera watsonii* in which severe Fe limitation (3 nM) showed strong negative changes in growth and N_2_-fixation, limiting both processes, while higher Fe levels (up to 400 nM) increased these two parameters (Jacq et al., 2014). N_2_-fixation is an Fe-dependent process, since nitrogenase complex contains 38 Fe atoms per holoenzyme (Hoffman et al., 2014). This Fe dependence was observed for *F. muscicola* at high PO_4_^3–^ levels (Figure 5B). However, in *Halothece* sp., high amounts of Fe (7.5 μM) inhibited N_2_-fixation but only under high PO_4_^3–^ levels, suggesting toxicity at these levels of PO_4_^3–^ and Fe for this species (Figures 3C, 4B, 5A). Nonetheless, Fe can control N_2_-fixation in *Halothece* sp. cells, considering the Fe dependence of their alkaline phosphatase D (PhoD), which releases PO_4_^3–^ from organic sources to fuel N_2_-fixation (Fernández-Juárez et al., 2019). Further studies must be conducted especially in *Halothece* sp. to determine which threshold of Fe concentrations can Fe inhibit/enhance growth and N_2_-fixation rates.

#### Interaction Between P and Fe Under Different Nitrogen Concentrations

##### Cell Recovery Under P, Fe, and N Limitation in *Halothece* sp.

It is well documented how unicellular cyanobacteria cells are able to recover their phenotype by a genetically determined program (Klotz et al., 2016). Re-inoculum of PO_4_^3–^ and Fe in [Low PO_4_^3–^-Low Fe] under NO_3_^–^ optimal conditions and even in [High PO_4_^3–^–High Fe] under extremely limiting NO_3_^–^ conditions with the addition of NO_3_^–^ had as a consequence the partial or complete recovery of the green natural color and growth of the cells (Figure 1C). However, here, we observed that for *Halothece* sp., this program is “canceled” under extreme NO_3_^–^ limitation and co-limitation of PO_4_^3–^ and Fe. Under extremely limiting NO_3_^–^ and [Low PO_4_^3–^–Low Fe] conditions, addition of PO_4_^3–^ and Fe did not result in growth recovery of the cells, suggesting that nutrient co-limitation (PO_4_^3–^, Fe, and NO_3_^–^) could kill cells irreversibly by breakage of cells (Figures 1C, 7E).

##### Nature of Nutrient Co-Limitation (P, Fe, and N) in *Halothece* sp. and *Fischerella muscicola*

In oligotrophic areas, e.g., the tropical North Atlantic, bacterial productivity and biomass are usually co-limited with N, P, and/or Fe (Mills et al., 2004; Arrigo, 2005; Moore et al., 2008). The interactions between these limiting nutrients can trigger different types of limitation: simultaneous co-limitation (nutrient limitation of, e.g., N, P, and/or Fe have collective responses), independent co-limitation (nutrient limitation of, e.g., N, P, and/or Fe have different responses), and serial limitation wherein the response of a second limiting nutrient is appreciable after the previous addition of the primary limiting nutrient (Harpole et al., 2011). To our knowledge, this study is the first to show evidences of serial limitation of N (NO_3_^–^) and P (PO_4_^3–^) in diazotrophs with combined N as the primary limiting nutrient as suggested by the results for *Halothece* sp. (Figure 3A). This suggests that in extremely N-limited waters, diazotrophs like *Halothece* sp. are not competitive enough to compensate for N-deficits of the system through their N_2_-fixation activities and cannot deplete the P concentration enough for the other co-occurring diazotrophic or non-diazotrophic species in the community to become P-limited. The reverse would follow for *F. muscicola*, which was independent of combined N source. The simultaneous co-limitation of PO_4_^3–^ and Fe observed in Figure 3C for *Halothece* sp. and *F. muscicola* may be due to the nature of the process of N_2_-fixation, which is ATP dependent, and Fe is a structural component of the nitrogenase enzyme (Hoffman et al., 2014). Iron (Fe) is also a co-factor of alkaline phosphatases, which is one of the P-acquisition mechanisms of diazotrophs when P is in short supply (Santos-Beneit, 2015; Fernández-Juárez et al., 2019). However, the mechanisms behind the simultaneous limitation of PO_4_^3–^ and Fe have to be further investigated as we also observed deleterious effects depending on the concentrations of these nutrients.

##### Differences in Nutrient Kinetics in Diazotrophs at Different P and Fe Concentrations

Of the two species of cyanobacteria tested, the one that can most probably adapt to low levels of PO_4_^3–^ is the unicellular cyanobacterium *Halothece* sp. based on their nutrient kinetics (Figure 4B). Based on the Monod curves, the unicellular strain had the lowest *K*_μ_ and *Q*_min_, indicating a better adaptation to PO_4_^3–^ than the filamentous diazotroph (Figure 4B), albeit the *K*_μ_ measured in *Halothece* sp. under [High Fe] (i.e., 10 nM) shows lower affinity for PO_4_^3–^ than that reported in the marine picophytoplankton *Synechococcus* (1 nM) (Kretz et al., 2015). In addition, its *Q*_min_ (2.27 μM) was much higher than that of the marine *Synechococcus* (3 nM) (Kretz et al., 2015), indicating that *Halothece* sp. is well adapted to P limitation but not at the level of *Synechococcus*. Unicellular cyanobacteria with smaller sizes are more efficient in acquiring nutrients in nutrient-limited waters because of their high surface: area ratios, while heterocyst-forming bacteria, which are generally bigger in size, need higher nutrient requirements (Haramaty et al., 2007). This is clearly shown in the Monod plot (Figure 4B), in which *F. muscicola* needs high amounts of Fe to growth, on the contrary of *Halothece* sp. in which high Fe levels inhibited cell abundance (Figures 3C, 4B).

#### Reactive Oxygen Species Production and Apoptotic Changes Derived From P, Fe, and N Limitation

Nutrient limitation (i.e., PO_4_^3–^, Fe, and/or NO_3_^–^) increased ROS in both species, *Halothece* sp. and *F. muscicola* (Figures 6A–C, 7A–D). Iron (Fe) had a key role in regulating ROS production. Increasing Fe levels can have different responses as shown in Figures 6A–C: (1) beneficial through increase activity of enzymatic antioxidant defenses or (2) extremely toxic through increase in Fenton and Haber–Weiss reaction (Latifi et al., 2009; Diaz and Plummer, 2018). The second case is suggested in Figure 6B, in which under [Low NO_3_^–^] and [High PO_4_^3–^], Fe increased ROS production and in turn can inhibit N_2_-fixation (Alquéres et al., 2010). This could explain why *Halothece* sp. under [High PO_4_^3–^–High Fe] had lower growth and N_2_-fixation rates than the other treatments with high levels of PO_4_^3–^ (Figure 5A). On the contrary, in *F. muscicola*, Fe generally reduced ROS production (Figure 6C). The reduction of ROS production with Fe addition is consistent with a study on *Anabaena* PCC 7120, in which ROS increased up to 10-fold when the cells were starved with Fe (Latifi et al., 2005). The results of our apoptosis tests (normally applied to eukaryotic cells) for *F. muscicola* when cells were limited by PO_4_^3–^ and/or Fe are first evidences of programmed cell death in cyanobacteria by nutrient limitation (Figures 7F–I). Further investigations have to be performed to figure out the molecular mechanisms behind this.

### Prediction of Genes Involved in P, Fe, and N Limitation in *Halothece* sp.

**FIGURE 9:**
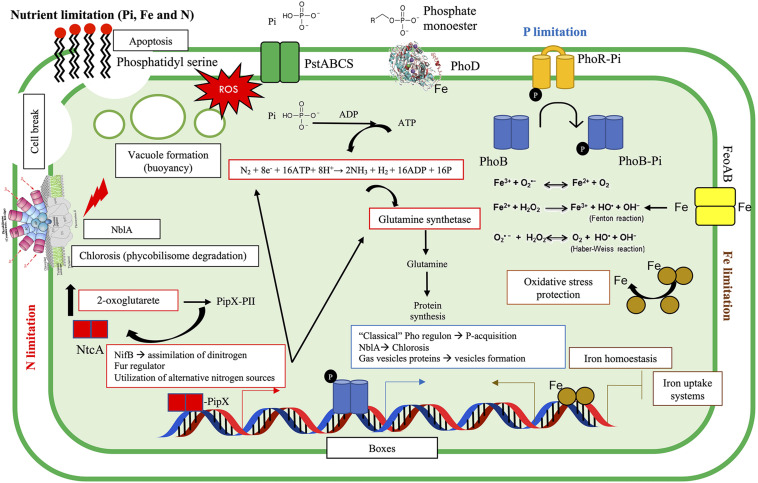
Summary of PO_4_^3–^, Fe, and NO_3_^–^ interactions and the physiological and molecular responses of the cyanobacteria tested. Nutrient limitation can result in chlorosis, apoptosis, vacuole formation, reactive oxygen species (ROS) production, and growth inhibition. In such situations, cells may activate different molecular mechanisms. Under PO_4_^3–^ limitation (blue), the autophosphorylation of PhoR allows the phosphorylation of PhoB, regulating the P-response (i.e., expression of the “classical” Pho regulon, e.g., alkaline phosphatase, PhoD). Fur is a repressor (although it can play as activator) that responds to Fe limitation (brown) and inhibits/allows the transcription of the Fur regulon, through the association with divalent metal (i.e., Fe). Nitrogen limitation (red) triggers different responses through NtcA regulator. When N abundance is low, 2-oxoglutarate (2-OG) may increase, and this could bind to a complex called PII–PipX and breaks this association, allowing the disassociation of PipX, and the formation of PipX–NtcA. PipX is a coactivator of NtcA, and it is suggested that it can stabilize NtcA and would help to recruit RNA polymerase (Tiwari et al., 2015; Kaushik et al., 2016; Giner-Lamia et al., 2017). PO_4_^3–^ is represented as Pi.

We predicted the Pho, Fur, and NtcA boxes in the unicellular cyanobacterium *Halothece* sp. (PCC 7418), controlled by the master regulators in P, Fe, and N metabolism (PhoB, Fur, and NtcA, respectively), showing well-conserved sequences in this bacteria (Figures 8A–C), consistent with several studies (Tiwari et al., 2015; Kaushik et al., 2016; Giner-Lamia et al., 2017). A large majority of these genes associated to these boxes were involved in inorganic ion transport and metabolism (P) (Figures 8D–F and Supplementary Tables S2–S4), providing key information of how cyanobacteria respond to P, Fe, and N limitation (Figure 9). We detected genes involved in typical transporters in P, Fe, and N metabolism, in Fe storage, cell wall biosynthesis, amino acid metabolism, photosynthesis, chlorosis, chlorophylls and PC biosynthesis, anti-phage systems (e.g., *cas* genes, implicated in the CRISPR-Cas systems), or even virulence (Supplementary Tables S2–S4). Through the created algorithms, we were able to detect elements from the “classical” Pho regulon (e.g., transporters, *pstABCS*, enzymes through which PO_4_^3–^ is obtained from dissolved organic phosphorus, APases or stored phosphate, *ppK*), Fe transporters (e.g., *feoA* and *feoB*), and a whole group of genes involved in N metabolism (e.g., *gltBNS*, *amt*, and *nifB*) and thus being the most probable genes controlled by these TFs as is already described in *Cyanothece*, *Gloeobacter*, *Microcystis*, *Nostoc*, *Prochlorococcus*, *Synechococcus*, *Synechocystis*, *Thermosynechococcus*, and *Trichodesmium* (Supplementary Material [Multifasta] and Supplementary Tables S2–S4). A N_2_-fixation regulator, *nifB*, was predicted under the control of NtcA, whose product is crucial in iron–molybdenum biosynthesis (Supplementary Table S4). Downstream of this gene, we localized an entire cluster of genes related to N_2_-fixation: Fd III 4Fe-4S, *nifS*, *nifU*, *nifH*, *nifD*, *nifK*, *nifZ*, *nifE*, *nifN*, *nifX*, DUF683, and *nifW*. In addition, some reports affirm that NtcA binds and controls Fur protein (López-Gomollón et al., 2007), as we predicted in this study, showing that N limitation can increase Fur protein levels and suggesting the narrow connection between N and Fe metabolism.

In summary, we show that two species of N_2_-fixing cyanobacteria potentially associated with seagrasses have differing sensibilities to PO_4_^3–^, Fe, and NO_3_^–^ concentrations, showing that these nutrients interact with each other based on our experimental and bio-informatic analysis. Despite the low number replications (being conscious that some results cannot be generalized), the growth responses reported here at varying concentrations of PO_4_^3–^ and Fe at different NO_3_^–^ conditions must be taken with precaution since the internal storage of these nutrients could affect these results considering that the cells were inoculated from optimal nutrients conditions. This is a pilot study in which insights on nutrient limitation in N_2_-fixers are reported. Nonetheless, our study of a multifactorial design provides useful data and important findings of nutrient limitation in marine diazotrophs (at physiological, biochemical, and genetic levels). Deeper molecular assays (i.e., transcriptomic or proteomic assays) are recommended to investigate all the predicted genes involved in P, Fe, and N metabolism.

## Data Availability Statement

The raw data supporting the article of this manuscript will be made available by the authors, without undue reservation, to any qualified researcher.

## Author Contributions

VF-J and NA designed the experiments. VF-J conducted all experiments. All authors led the writing of the manuscript, reviewed, and supervised by NA.

## Conflict of Interest

The authors declare that the research was conducted in the absence of any commercial or financial relationships that could be construed as a potential conflict of interest.

## Footnotes

1 http://genome2d.molgenrug.nl/g2d_pepper_motifs.php

## Supplementary Material

Supplementary Table 1 List of all experimental treatments conducted in this study.

Supplementary Table 2 Pho regulon prediction. PHO boxes sequences, their position in the genome, orientation, the genes that are controlling (the Pho regulon), locus tag from these genes, the distance of the box from the gene and the gen function from single-letter of functional (COGs) are presented.

Supplementary Table 3 Fur regulon prediction. Fur boxes sequences, their position in the genome, orientation, the genes that are controlling (the Fur regulon), locus tag from these genes, the distance of the box from the gene and the gen function from single-letter of functional (COGs) are presented.

Supplementary Table 4 NtcA regulon prediction. NtcA boxes sequences, their position in the genome, orientation, the genes that are controlling (the NtcA regulon), locus tag from these genes, the distance of the box from the gene and the gen function from single-letter of functional (COGs) are presented.
