# Supplementary material for: Differential Effects of Varying Concentrations of Phosphorus, Iron, and Nitrogen in N2-Fixing Cyanobacteria
Source: Front Microbiol. 2020 Sep 25;11:541558. doi: 10.3389/fmicb.2020.541558 (PMC7546424; doi:10.3389/fmicb.2020.541558)
Supplement: Supplementary Table 1 — List of all experimental treatments conducted in this study. [file Data_Sheet_1.PDF]

Supplementary Table 1. List of all experimental treatments conducted in this study.

**Experiments on *Halothece* sp. PCC 7418 and *Fischerella muscicola* PCC 73103**			
**Treatments**			**Description**
[Low PO_4^{3-}- Low Fe]	[Medium PO_4^{3-}- Low Fe]	[High PO_4^{3-}- Low Fe]	1st experiment<br>Optimal NO_3^- (4.4 mM) for *Halothece* sp.
[Low PO_4^{3-}- Medium Fe]	[Medium PO_4^{3-}- Medium Fe]	[High PO_4^{3-}- Medium Fe]	2nd experiment<br>Low NO_3^- (0.15 mM) for *Halothece* sp. and N_2 as sole N source for *F. muscicola*
[Low PO_4^{3-}- High Fe]	[Medium PO_4^{3-}- High Fe]	[High PO_4^{3-}- High Fe]	3rd experiment<br>Extremely limiting NO_3^- conditions (6.66 nM), comparing with optimal NO_3^- in selected treatments ([Low PO_4^{3-}- Low Fe], [High PO_4^{3-}- Low Fe], [Low PO_4^{3-}- High Fe] and [High PO_4^{3-}- High Fe]) for *Halothece* sp.
**Recovery experiments on *Halothece* sp. PCC 7418**			
**Initial Treatment**	**Condition of NO_3^-**	**Nutrient added (at day 12)**	**Resulting treatment (maintained for 4 days)**
[Low PO_4^{3-}- Low Fe]	Optimal NO_3^- (4.4 mM)	PO_4^{3-} and Fe	[High PO_4^{3-}- High Fe] in optimal NO_3^- treatment
[Low PO_4^{3-}- Low Fe]	Extremely limiting NO_3^- conditions (6.66 nM)	PO_4^{3-}, Fe and NO_3^-	[High PO_4^{3-}- High Fe] in optimal NO_3^- treatment
[High PO_4^{3-}- High Fe]	Extremely limiting NO_3^- conditions (6.66 nM)	NO_3^-	[High PO_4^{3-}- High Fe] in optimal NO_3^- treatment

In the recovery experiments, PO_4^{3-}, Fe, and/or NO_3^- were added to the different initial treatments to achieve optimal conditions (45 µM, 7.5 µM and 4.4 mM, respectively). Initial treatment for PO_4^{3-}: [Low PO_4^{3-}] (0.1 µM), [Medium PO_4^{3-}] (1 µM) and [High PO_4^{3-}] (45 µM). Initial treatments for Fe: [Low Fe] (2 nM), [Medium Fe] (20 nM) and [High Fe] (7.5 µM).

Supplementary multifasta 1, for PHO boxes in cyanobacteria:

>SYNPCC7002_A1357(SYNPCC7002_A1357) Score=7.8 Pos=-125 [Synechococcus sp. PCC 7002]
GCTAACCTNNNCTATATTTNNNTTTTATTT
>SYNPCC7002_A2352(phoA1) Score=7.5 Pos=-182 [Synechococcus sp. PCC 7002]
ATTTAATTNNNTTTAGTTGNNNCTTAGCTA
>SYNPCC7002_A2352(phoA1) Score=9.5 Pos=-91 [Synechococcus sp. PCC 7002]
ATTAAACTNNNCTTAGTTTNNNTTTAACTC
>SYNPCC7002_A1232(cysR) Score=8.7 Pos=-126 [Synechococcus sp. PCC 7002]
CTTAAACTNNNGATTACATNNNATTTAACT
>SYNPCC7002_A2120(ndbB) Score=7.6 Pos=-309 [Synechococcus sp. PCC 7002]
CTAAAATTNNNTTTTACTANNNTTTGATCA
>sll0679(sphX) Score=10.9 Pos=-135 [Synechocystis sp. PCC 6803]
TTTAACCANNNCTTTACTANNNCTTAACCT
>sll0654(phoA) Score=7.3 Pos=-210 [Synechocystis sp. PCC 6803]
TTTTACTTNNNCTTTCCCTNNNGTTAGCAA
>sll0654(phoA) Score=8.8 Pos=-175 [Synechocystis sp. PCC 6803]
CTTAACCTNNNCATAGTCTNNNCATAAGTT
>slr1247(pstS) Score=10.5 Pos=-342 [Synechocystis sp. PCC 6803]
CTTAATCTNNNCTTAATTCNNNCTTAATTT
>slr0115(rpaA) Score=7 Pos=-174 [Synechocystis sp. PCC 6803]
ATTACCCANNNTTTAGATGNNNTTTTTCTT
>cce_1163(pstS) Score=9.7 Pos=-109 [Cyanothece sp. ATCC 51142]
CTTAATCTNNNTTTTACTGNNNTTTAACCC
>cce_1859(sphX) Score=8.8 Pos=-141 [Cyanothece sp. ATCC 51142]
CTTAATAANNNGTTTAACTNNNCTTCATAT
>cce_0886(pstS) Score=7.5 Pos=-53 [Cyanothece sp. ATCC 51142]
GTTAGATTNNNCTTTAAGANNNGTTTAGTT
>cce_0886(pstS) Score=6.9 Pos=-358 [Cyanothece sp. ATCC 51142]
CTTGAAAANNNTATAAATANNNGTTATCAT
>cce_3317(SYNPCC7002_A1357) Score=8.4 Pos=-44 [Cyanothece sp. ATCC 51142]
CTTATCTANNNCTTTATTTNNNTTTATACT
>cce_1211(purF) Score=7.7 Pos=-143 [Cyanothece sp. ATCC 51142]
TATAATCTNNNGTTTTATTNNNGTCTATTT
>cce_4621(phoA) Score=8.7 Pos=-88 [Cyanothece sp. ATCC 51142]
TTTAAAGTNNNTTTAACATNNNCTTTAAAT
>cce_4758(fbp) Score=8.3 Pos=-125 [Cyanothece sp. ATCC 51142]
ATTAACCTNNNCTGAAATTNNNCTAAAGTT
>cce_5183(ackA) Score=9.6 Pos=-74 [Cyanothece sp. ATCC 51142]
GTTAATTTNNNTTTAATCANNNCTTAAAAT
>cce_4392(cce_4392) Score=8.8 Pos=20 [Cyanothece sp. ATCC 51142]
CTTTAACCNNNCTCAATCTNNNCTTAAACT
>cce_5174(ppk) Score=8.4 Pos=-70 [Cyanothece sp. ATCC 51142]
GATAAAAANNNCATAAAGTNNNCTTAAACT
>cce_0154(ndbB) Score=7.6 Pos=-83 [Cyanothece sp. ATCC 51142]
TTTTATCTNNNGATAATTANNNATTAATGA
>PCC8801_1024(pstS) Score=9.8 Pos=-108 [Cyanothece sp. PCC 8801]
TTTTATCANNNGTTTACCTNNNCTTAACCC
>PCC8801_4067(sphX) Score=8.1 Pos=-157 [Cyanothece sp. PCC 8801]
CTTAGCATNNNCTTTTTCTNNNGCTAAACT
>PCC8801_1433(spoT) Score=8.5 Pos=-224 [Cyanothece sp. PCC 8801]
TTTAACAGNNNCTAAACCTNNNTTTAAACT
>PCC8801_1847(SYNPCC7002_A1357) Score=7.5 Pos=-43 [Cyanothece sp. PCC 8801]
AATAAATTNNNTCTTATTTNNNATTATCTT

>PCC8801_4178(purF) Score=7.5 Pos=-104 [Cyanothece sp. PCC 8801]
GATAAGATNNNGTTAAAGANNNGTTAAAAT
>PCC8801_1683(nucH) Score=9.8 Pos=-246 [Cyanothece sp. PCC 8801]
ATTAAACTNNNCTTAAATTNNNGTTAAATA
>PCC8801_0430(fbp) Score=7.9 Pos=-278 [Cyanothece sp. PCC 8801]
TTAAAATTNNNTTTTATTTNNNCTTTTATT
>PCC8801_0346(cysR) Score=9.7 Pos=-91 [Cyanothece sp. PCC 8801]
TTTAATCTNNNGATTACCTNNNGTTTACCT
>PCC8801_3887(ackA) Score=9.1 Pos=-183 [Cyanothece sp. PCC 8801]
CTTTGACCNNNGTTTACCANNNCTTAACCT
>PCC8801_1625(ppk) Score=7.2 Pos=-95 [Cyanothece sp. PCC 8801]
TATTACCTNNNTATAGCCTNNNGATAGCCT
>PCC8801_3662(rsbU) Score=7.4 Pos=-162 [Cyanothece sp. PCC 8801]
ATTAACGCNNNGATTAACCNNNCCTAATTA
>PCC8801_2302(SYNPCC7002_A0979) Score=7.1 Pos=-232 [Cyanothece sp. PCC 8801]
CATAATGANNNTTTTAAGANNNGTTAAAAA
>Cyan7425_0207 Score=7.8 Pos=-81 [Cyanothece sp. PCC 7425]
TTTAACGANNNCTTTAGCANNNGATTACCT
>Cyan7425_4261(phoA1) Score=9 Pos=-146 [Cyanothece sp. PCC 7425]
ATTAATCANNNGTTTACCTNNNCATAAATC
>Cyan7425_4387(SYNPCC7002_A2263) Score=7.5 Pos=-102 [Cyanothece sp. PCC 7425]
ATTATCATNNNTATTACCTNNNCCTTACCC
>Cyan7425_1977(ppk) Score=8.4 Pos=-238 [Cyanothece sp. PCC 7425]
GTTATCCCNNNGTTAATTTNNNATTAAATC
>Cyan7425_0664(pstS) Score=10.1 Pos=-80 [Cyanothece sp. PCC 7425]
ATTAATCTNNNCTTAATCTNNNCTTTATCC
>MAE_18380(pstS) Score=9.5 Pos=-87 [Microcystis aeruginosa NIES-843]
TTTATTCANNNCTTAACCTNNNCTTTACCA
>MAE_23860(fbp) Score=7.6 Pos=-129 [Microcystis aeruginosa NIES-843]
ATTAGCCANNNCTAAAACTNNNCTAAAAAT
>MAE_18440(cysR) Score=9.6 Pos=-86 [Microcystis aeruginosa NIES-843]
GTTAATCTNNNGATTACCTNNNGTTTACCT
>MAE_02800(ackA) Score=8.8 Pos=-66 [Microcystis aeruginosa NIES-843]
TTTTATCTNNNCTTAACCTNNNTTTGATTA
>MAE_18310(pstS) Score=9.6 Pos=-139 [Microcystis aeruginosa NIES-843]
ATTAACCGNNNTTTTACCGNNNCTTAACCA
>all3651(purF) Score=7.7 Pos=-119 [Nostoc sp. PCC 7120]
TTTAATATNNNGTTAAAGANNNGTTAAGAC
>alr5291(phoA1) Score=10.4 Pos=-229 [Nostoc sp. PCC 7120]
GTTAACCTNNNTTTATATTNNNCTTAACTT
>all4021(fbp) Score=7 Pos=-136 [Nostoc sp. PCC 7120]
CGTAAACTNNNGATTTCTTNNNTTTATCTA
>alr5259(SYNPCC7002_A2263) Score=8.7 Pos=-329 [Nostoc sp. PCC 7120]
GTAAAACTNNNTTTATTTANNNCTTAATTT
>all1758(rsbU) Score=7.5 Pos=-49 [Nostoc sp. PCC 7120]
ATTTATTANNNGTCAATCTNNNATTAACCC
>all0129(rpaA) Score=7.3 Pos=-101 [Nostoc sp. PCC 7120]
TTTATGTTNNNTTTAAAATNNNTTTTATAA
>all3822(SYNPCC7002_A0979) Score=8.6 Pos=-149 [Nostoc sp. PCC 7120]
CTTAATTTNNNCTTAAGTANNNCTCAAATT
>alr4975(alr4975) Score=7.6 Pos=-10 [Nostoc sp. PCC 7120]
ATTAATTANNNTGTTACCTNNNATTTATAT
>alr2234(phoD) Score=9.6 Pos=-275 [Nostoc sp. PCC 7120]
ATTAACCTNNNCTTAGTCANNNATTAATTT
>all0207(phoD) Score=9.3 Pos=-98 [Nostoc sp. PCC 7120]

ATTAACCCNNNGATAACTCNNNCTTTACTT
>all0911(pstS) Score=10.8 Pos=-105 [Nostoc sp. PCC 7120]
CTTAACTTNNNGTTTACCTNNNCTTAACTT
>all4575(pstS) Score=8 Pos=-324 [Nostoc sp. PCC 7120]
TTTTATCTNNNCTTTTATTNNNCTTTTTTT
>Tery_3534(pstS) Score=9.7 Pos=-192 [Trichodesmium erythraeum IMS101]
TTTGATATNNNTTTAACCTNNNCTTAATCT
>Tery_2653(spoT) Score=7.8 Pos=-43 [Trichodesmium erythraeum IMS101]
AATAAACTNNNATTTGATANNNATTAACTC
>Tery_4322(SYNPCC7002_A1357) Score=7 Pos=-395 [Trichodesmium erythraeum IMS101]
CTTATTATNNNAATTATTTNNNATTTAGCT
>Tery_3699(purF) Score=8.3 Pos=-97 [Trichodesmium erythraeum IMS101]
GTTAAGGANNNCTTAAAGTNNNTTTAAGCT
>Tery_0682(fbp) Score=6.5 Pos=-336 [Trichodesmium erythraeum IMS101]
TTTTAGATNNNGTTTTTGTNNNCATTATCA
>Tery_2568(SYNPCC7002_A2263) Score=7.5 Pos=-208 [Trichodesmium erythraeum IMS101]
GATAAGATNNNATTAACCTNNNGTTTGCTA
>Tery_0739(rsbU) Score=8.3 Pos=-250 [Trichodesmium erythraeum IMS101]
TTAAAACTNNNCTTAATCTNNNACTAACTC
>Tery_4937(rpaA) Score=7 Pos=-158 [Trichodesmium erythraeum IMS101]
CTTGACAANNNAATAAATTNNNGTTAAAGA
>Tery_0954(SYNPCC7002_A0979) Score=8.3 Pos=-309 [Trichodesmium erythraeum IMS101]
ATTAACCANNNATTGAATTNNNATTGACTT
>Tery_2902(sphR) Score=10.4 Pos=-365 [Trichodesmium erythraeum IMS101]
GATAACCCNNNGTTAACCTNNNCTTAACCT
>Tery_3661(ndbB) Score=7.8 Pos=-342 [Trichodesmium erythraeum IMS101]
TTTTAATANNNAATTACCCNNNCTTAGCCT
>Synpcc7942_0004(purF) Score=7.8 Pos=-76 [Synechococcus elongatus PCC 7942]
GTTAAGTCNNNGTTAAATTNNNATTAGCCG
>Synpcc7942_1392(phoA) Score=9.4 Pos=-233 [Synechococcus elongatus PCC 7942]
TTTAACTANNNCATAATCTNNNCTCAATCT
>CYA_1552(pstS) Score=10.2 Pos=-203 [Synechococcus sp. JA-3-3Ab]
AATAACCTNNNTTTAACCTNNNGTTAACCA
>CYA_1732(pstS) Score=9.6 Pos=-75 [Synechococcus sp. JA-3-3Ab]
GTTAACCTNNNGATATCCTNNNGTTAACTT
>CYA_1541(cysR) Score=10.5 Pos=-172 [Synechococcus sp. JA-3-3Ab]
CTTAACCTNNNCATAACCTNNNCTTTACTT
>CYA_2506(phoD) Score=7.9 Pos=-129 [Synechococcus sp. JA-3-3Ab]
CTTAAACANNNGCTAACCCNNNTTTCACCG
>SYNW2391(phoA1) Score=9.2 Pos=-78 [Synechococcus sp. WH 8102]
TTTGATCANNNCTTAAACTNNNCCTAACTT
>tlr2164(pstS) Score=10.9 Pos=-51 [Thermosynechococcus elongatus BP-1]
TTTAAACANNNTTTTACCTNNNCTTAACTT
>tll1671(cysR) Score=8.1 Pos=-171 [Thermosynechococcus elongatus BP-1]
CTTAACCCNNNCTTGAACCNNNGATTATCT
>null(SYNPCC7002_A1357) Score=7.1 Pos=-268 [Thermosynechococcus elongatus BP-1]
ATTAAAAANNNATTAAAAANNNTTTCTCTT

Supplementary multifasta 2, for Fur boxes in cyanobacteria:

>SYNPCC7002_A2347(chlL) Score=3.6 Pos=-126 [Synechococcus sp. PCC 7002]
TTTTATAAAAACTCTAAGT
>SYNPCC7002_G0137(exbB) Score=4.2 Pos=-279 [Synechococcus sp. PCC 7002]
ATTAAGAGATTATCTCAAT
>SYNPCC7002_G0099(pchR) Score=3.7 Pos=-94 [Synechococcus sp. PCC 7002]
ATTTCTTATTAATATAAAT
>SYNPCC7002_G0006(iutA) Score=4.6 Pos=-38 [Synechococcus sp. PCC 7002]
TTTGAGAATTATTTTTAGT
>SYNPCC7002_A1631(apcF) Score=3.3 Pos=-257 [Synechococcus sp. PCC 7002]
TTTTATTATTTTTTTTAAA
>SYNPCC7002_A0913(SYNPCC7002_A0913) Score=2.2 Pos=-146 [Synechococcus sp. PCC 7002]
TTTTTACAACATTTTTAAA
>SYNPCC7002_A0913(SYNPCC7002_A0913) Score=3.8 Pos=-68 [Synechococcus sp. PCC 7002]
AATGAGAAAATTTTGATAT
>SYNPCC7002_A1018(chlH) Score=3.8 Pos=-57 [Synechococcus sp. PCC 7002]
ATAGTTATAAATTTTTAAT
>SYNPCC7002_A1443(nifJ) Score=2.2 Pos=-165 [Synechococcus sp. PCC 7002]
TTTTCTTATAACTTTTAGC
>SYNPCC7002_A0871(afuC) Score=5.1 Pos=-83 [Synechococcus sp. PCC 7002]
GTTGAGAATAGTTCTTAAT
>SYNPCC7002_A2351(SYNPCC7002_A2351) Score=2.9 Pos=-216 [Synechococcus sp. PCC 7002]
TTTATTATTGTTTTTTAGT
>SYNPCC7002_A1961(psaA) Score=3.4 Pos=-206 [Synechococcus sp. PCC 7002]
AGTTTTAAATATTGTTAAT
>SYNPCC7002_G0061(SYNPCC7002_G0061) Score=4.1 Pos=-62 [Synechococcus sp. PCC 7002]
ATTGATAAATAATTTAAGT
>SYNPCC7002_A1649(fur) Score=3.5 Pos=-88 [Synechococcus sp. PCC 7002]
GTTAGTATTTATTTGCAAT
>SYNPCC7002_G0138(iutA) Score=4.6 Pos=-67 [Synechococcus sp. PCC 7002]
ATTGAGATAATCTCTTAAT
>SYNPCC7002_G0090(SYNPCC7002_G0090) Score=5.2 Pos=-83 [Synechococcus sp. PCC 7002]
ATTGAAATAAATTCTTATT
>SYNPCC7002_G0104(pchR) Score=5.4 Pos=-68 [Synechococcus sp. PCC 7002]
ATTGAGAATAATTAGTAAT
>SYNPCC7002_G0103(fhuA) Score=3.2 Pos=-46 [Synechococcus sp. PCC 7002]
ATTAAGAACTTTTTGAAGT
>slr0749(chlL) Score=3.3 Pos=-173 [Synechocystis sp. PCC 6803]
ATTTTATTTTTGTCTCAAT
>ssr2333(feoA) Score=4.5 Pos=-31 [Synechocystis sp. PCC 6803]
GTTGAGAATTATTTGCAGT
>sll1406(fhuA) Score=3.4 Pos=-38 [Synechocystis sp. PCC 6803]
ATTAATAAAACTTTTTAAC
>sll1404(exbB) Score=6 Pos=-110 [Synechocystis sp. PCC 6803]
ATTGAAAATAGTTATCAAT
>slr1490(fhuA) Score=4.1 Pos=-86 [Synechocystis sp. PCC 6803]
TTTGAGAATTAGTTGCAGT
>slr1484(SYNPCC7002_G0090) Score=5.6 Pos=-255 [Synechocystis sp. PCC 6803]
ATTGATAACTATTTTCAAT

>slr1318(fecE) Score=4.1 Pos=-176 [Synechocystis sp. PCC 6803]
ATTCCTAATTATTCTTAAC
>slr1485(SYNPCC7002_G0089) Score=2.2 Pos=-9 [Synechocystis sp. PCC 6803]
GTTTTAATAATGTTTAAAT
>slr1316(fecC) Score=4.2 Pos=-78 [Synechocystis sp. PCC 6803]
ATTGATAATCTTTCCTAGT
>slr1485(SYNPCC7002_G0089) Score=2.7 Pos=-42 [Synechocystis sp. PCC 6803]
ACTGTGAAATTTATTTAAC
>sll1404(exbB) Score=4.6 Pos=-64 [Synechocystis sp. PCC 6803]
ATTGAGAATTACTCTTAAC
>sll1404(exbB) Score=4.6 Pos=-286 [Synechocystis sp. PCC 6803]
TATGTGAAATATTATTATT
>slr1295(sufA) Score=4.9 Pos=-44 [Synechocystis sp. PCC 6803]
ATTGAGAATTACTTTTATT
>sll1911(SYNPCC7002_A0913) Score=4.2 Pos=-59 [Synechocystis sp. PCC 6803]
GTTGTTAAAATTTAACAAT
>slr0513(sufA) Score=3.9 Pos=-240 [Synechocystis sp. PCC 6803]
AATAATAATCTCTTGCAAT
>slr0513(sufA) Score=4.1 Pos=-187 [Synechocystis sp. PCC 6803]
ATTGCACTTTATTTGCAAT
>sll0849(psbD) Score=3.3 Pos=-298 [Synechocystis sp. PCC 6803]
AATGTAAAATATTTGCTAA
>slr1181(psbA) Score=2.8 Pos=-242 [Synechocystis sp. PCC 6803]
ATTAAAATCTTTTTTTTAC
>slr1181(psbA) Score=4 Pos=-182 [Synechocystis sp. PCC 6803]
TTAAAGAAATATTATTAAT
>sll1867(psbA) Score=3.4 Pos=-124 [Synechocystis sp. PCC 6803]
ATTTACAAATTGTTACAAT
>slr1738(perR) Score=3 Pos=-194 [Synechocystis sp. PCC 6803]
ATTAATATTTTTTTTATAA
>sll0247(isiA) Score=2.8 Pos=-269 [Synechocystis sp. PCC 6803]
ATTTCTTAATAATTTTAGT
>ssr2333(feoA) Score=4.4 Pos=-69 [Synechocystis sp. PCC 6803]
TTTGATATTTATTCTGAAC
>slr1392(feoB) Score=4.5 Pos=-278 [Synechocystis sp. PCC 6803]
GTTGAGAATTATTTGCAGT
>cce_4533(cce_4533) Score=3.7 Pos=-118 [Cyanothece sp. ATCC 51142]
TTTGAGTTTTATTTAAAAT
>cce_0033(feoA) Score=4.5 Pos=-47 [Cyanothece sp. ATCC 51142]
GATAAGAATTATTCTTAAT
>cce_0033(feoA) Score=3.3 Pos=-298 [Cyanothece sp. ATCC 51142]
ATTAAAAAATACTGTTAAC
>cce_0660(psbD) Score=3.4 Pos=-298 [Cyanothece sp. ATCC 51142]
ATTGTAAATTTTTTGCTAA
>cce_0479(dpsA) Score=4.7 Pos=-144 [Cyanothece sp. ATCC 51142]
GATGCAATATATTCTCAAT
>cce_2632(sufA) Score=4.8 Pos=-20 [Cyanothece sp. ATCC 51142]
ATCAATAATAATTTTCAAT
>cce_4358(chlH) Score=2.7 Pos=-183 [Cyanothece sp. ATCC 51142]
AATAAATAATTTTTGTAAA
>cce_4358(chlH) Score=2.3 Pos=-138 [Cyanothece sp. ATCC 51142]
ATTTGTCAATATTTGTAAC
>cce_3801(afuC) Score=4.2 Pos=-163 [Cyanothece sp. ATCC 51142]
ATTGAAAATTAATGCTAAT
>cce_0989(psaA) Score=3.4 Pos=-205 [Cyanothece sp. ATCC 51142]

AATTTTAATTATTGTTAAG
>cce_3809(SYNPCC7002_A2351) Score=3.2 Pos=-132 [Cyanothece sp. ATCC 51142]
GTTTTTATTTTCTGTCAAT
>cce_1977(coxB) Score=3.7 Pos=-136 [Cyanothece sp. ATCC 51142]
AATTTCAATCAATTTCAAT
>cce_0019(SYNPCC7002_A0913) Score=3.1 Pos=-75 [Cyanothece sp. ATCC 51142]
TTTTTTAATATTTTTTGAT
>cce_1944(apcF) Score=4.4 Pos=-151 [Cyanothece sp. ATCC 51142]
ATTGCTAAAATCTATAATT
>cce_3895(SYNPCC7002_G0062) Score=2.8 Pos=16 [Cyanothece sp. ATCC 51142]
GTTTAAAAATTCTTTTAGT
>cce_0892(fdx) Score=3 Pos=-29 [Cyanothece sp. ATCC 51142]
TTTCTTATAATTTCTTAAC
>cce_1785(isiA) Score=4.4 Pos=-18 [Cyanothece sp. ATCC 51142]
ATTGCAAATCTTTTTAAAT
>cce_3031(isiB) Score=4.9 Pos=-66 [Cyanothece sp. ATCC 51142]
ATTGAGAATTATTCTAAAC
>cce_2330(fur) Score=5.1 Pos=-96 [Cyanothece sp. ATCC 51142]
GTTGCTAATTATTTTCAAT
>PCC8801_0791(chlL) Score=2.7 Pos=-242 [Cyanothece sp. PCC 8801]
ATTTAGTAAATTTTAAAGT
>PCC8801_2575(SYNPCC7002_A2351) Score=3.1 Pos=-247 [Cyanothece sp. PCC 8801]
ATTTCTCCTTATTTTTAAT
>PCC8801_0349(sll1407) Score=3.3 Pos=-107 [Cyanothece sp. PCC 8801]
AATAATTAATTTTTTTTAT
>PCC8801_0256(ctaA) Score=2.2 Pos=-185 [Cyanothece sp. PCC 8801]
TTTTATTAACTTATGTAAA
>PCC8801_2812(SYNPCC7002_A0913) Score=4.4 Pos=-67 [Cyanothece sp. PCC 8801]
TATGATAAAATTTATTAAA
>PCC8801_4320(chlH) Score=2.3 Pos=-186 [Cyanothece sp. PCC 8801]
ATTTGTCAATATTTGTAAC
>PCC8801_0567(coxB) Score=2.7 Pos=-152 [Cyanothece sp. PCC 8801]
TTTGTTTATTATTTGTTAC
>PCC8801_3918(nifJ) Score=3.4 Pos=-67 [Cyanothece sp. PCC 8801]
TTTGTCAATAATTTGTAAC
>PCC8801_1443(sufA) Score=4 Pos=-90 [Cyanothece sp. PCC 8801]
ATTAGGAATAATTAGCAAC
>PCC8801_1928(SYNPCC7002_G0061) Score=2 Pos=-184 [Cyanothece sp. PCC 8801]
AATATTTTATATTTATAGT
>PCC8801_2860(fdx) Score=3.8 Pos=-49 [Cyanothece sp. PCC 8801]
ATTGTAAAAAAATATTAAC
>PCC8801_0870(fur) Score=4.6 Pos=-97 [Cyanothece sp. PCC 8801]
GTTGCAAATTATTTGCAAT
>PCC8801_3039(SYNPCC7002_G0098) Score=5.5 Pos=-89 [Cyanothece sp. PCC 8801]
GTTGACAAAAATTCTCATT
>PCC8801_3017(exbB) Score=5.7 Pos=-38 [Cyanothece sp. PCC 8801]
ATTGATAACTTTTCTCAAT
>Cyan7425_2411(pchR) Score=4.9 Pos=-71 [Cyanothece sp. PCC 7425]
ATTGTTAATCTTTTTCATT
>Cyan7425_2410(fhuA) Score=5.8 Pos=-56 [Cyanothece sp. PCC 7425]
ATTGAGAATAATTCTTATT
>Cyan7425_2418(exbB) Score=4.6 Pos=-264 [Cyanothece sp. PCC 7425]
ATTGATGATTATTTTCAAC
>Cyan7425_2438(pchR) Score=4.5 Pos=-37 [Cyanothece sp. PCC 7425]
ATTAAGAATAAGTCTCAGT

>Cyan7425_2439(fhuA) Score=5.1 Pos=-66 [Cyanothece sp. PCC 7425]
ATTGAGAGTATTTCTTATT
>Cyan7425_1597(perR) Score=4.9 Pos=-88 [Cyanothece sp. PCC 7425]
TTTGAGAATTATACTAAAT
>Cyan7425_5031(psbD) Score=3.1 Pos=-91 [Cyanothece sp. PCC 7425]
GTTTATAAACTTTCGTAAA
>Cyan7425_4671(psaA) Score=3.4 Pos=-292 [Cyanothece sp. PCC 7425]
TCTATGAATTTTTATTAAT
>Cyan7425_3510(afuC) Score=4 Pos=-60 [Cyanothece sp. PCC 7425]
GTTGAATAATAGTCTTAAT
>Cyan7425_3510(afuC) Score=5.3 Pos=-105 [Cyanothece sp. PCC 7425]
ATTGAGAATAATTTTCAAA
>Cyan7425_1838(apcF) Score=3 Pos=-245 [Cyanothece sp. PCC 7425]
TTTTATATTATCTTTTAAC
>Cyan7425_1059(psbA) Score=3.4 Pos=-182 [Cyanothece sp. PCC 7425]
ATTATTAATAAGTTTCAAA
>Cyan7425_2984(cce_4396) Score=2.7 Pos=-78 [Cyanothece sp. PCC 7425]
TATATGAATATCTTGTAAT
>Cyan7425_2984(cce_4396) Score=3.9 Pos=-48 [Cyanothece sp. PCC 7425]
ATTGCAATTATATTACAAT
>Cyan7425_2434(isiA) Score=4.7 Pos=-61 [Cyanothece sp. PCC 7425]
ATTGAGTTTATTTCTCAAC
>Cyan7425_3508(sufA) Score=3.5 Pos=-165 [Cyanothece sp. PCC 7425]
ATTAGGAATGAATCGTAAT
>MAE_16250(chlN) Score=2.8 Pos=-107 [Microcystis aeruginosa NIES-843]
ATTAAGTAATTCTTTATAT
>MAE_16230(chlL) Score=2.8 Pos=-150 [Microcystis aeruginosa NIES-843]
ATTTATATAATTTTTAAGC
>MAE_12190(chlH) Score=2.8 Pos=-247 [Microcystis aeruginosa NIES-843]
ATTAAATATTAATTTTTGT
>MAE_41160(psbD) Score=2.9 Pos=-274 [Microcystis aeruginosa NIES-843]
ATTTGTAATTTTTTGCTAA
>MAE_47560(psaA) Score=3.2 Pos=-297 [Microcystis aeruginosa NIES-843]
ATTAATAAACTTTTGTAAA
>MAE_22930(coxB) Score=2.5 Pos=-216 [Microcystis aeruginosa NIES-843]
TTTTAGATATTCTTTAGAT
>MAE_38140(nifJ) Score=3.9 Pos=-292 [Microcystis aeruginosa NIES-843]
ATTCTGAATATTAATTAAT
>MAE_12460(afuC) Score=4.4 Pos=-117 [Microcystis aeruginosa NIES-843]
TTTGAGAAAGTTTCCTAAT
>MAE_56680(sufA) Score=4.9 Pos=-61 [Microcystis aeruginosa NIES-843]
GATAAGAATTATTCTCAAT
>MAE_22920(ctaA) Score=2.5 Pos=-282 [Microcystis aeruginosa NIES-843]
TCTTTTCAATATTTTTAAT
>all4365(chlH) Score=2.9 Pos=-272 [Nostoc sp. PCC 7120]
GTTTTTTATTTTTTTCATC
>all4365(chlH) Score=3 Pos=-232 [Nostoc sp. PCC 7120]
AATATTAATTTTTCTTTAC
>alr5154(psaA) Score=3.4 Pos=-177 [Nostoc sp. PCC 7120]
ATTTTGAATTATTGTTAAA
>alr0950(coxB) Score=3.7 Pos=-134 [Nostoc sp. PCC 7120]
TTTACTCAAGATTATTAAT
>alr2803(nifJ) Score=3.8 Pos=-92 [Nostoc sp. PCC 7120]
ATTTTTAATCAGTATTAAT
>alr3808(dpsA) Score=3.5 Pos=-204 [Nostoc sp. PCC 7120]

GTTTAGAAATTATTGCAAT
>alr2514(coxB) Score=3 Pos=-190 [Nostoc sp. PCC 7120]
AGTTAAAAAGTTAATAAGT
>all4001(isiA) Score=4.8 Pos=-161 [Nostoc sp. PCC 7120]
ATTAAGAATCTTTTTCAAT
>alr3727(psbA) Score=3.7 Pos=-105 [Nostoc sp. PCC 7120]
TTTAGTAATATTAATTAAT
>alr4592(psbA) Score=4.1 Pos=-28 [Nostoc sp. PCC 7120]
ATAAATAATTAATCGCAAT
>asl0884(fdx) Score=3.5 Pos=-90 [Nostoc sp. PCC 7120]
ATTCTTAATTTTTTGTAAT
>alr3155(SYNPCC7002_G0061) Score=4.1 Pos=-98 [Nostoc sp. PCC 7120]
ATTACGAATTAATATAAAT
>all3903(cce_4396) Score=4.6 Pos=-47 [Nostoc sp. PCC 7120]
TTTAATATAAATTGTCAAT
>all2367(all2367) Score=3.2 Pos=-181 [Nostoc sp. PCC 7120]
TATTGTAATATCTTTTAAT
>alr2405(isiB) Score=5 Pos=-235 [Nostoc sp. PCC 7120]
ATTGAAATAAATATTCAAT
>all1691(fur) Score=5.2 Pos=-94 [Nostoc sp. PCC 7120]
TTTAATAAATATTCTCAAT
>all1692(sigC) Score=3.5 Pos=-320 [Nostoc sp. PCC 7120]
AATAAACATAATTTTTACT
>all2158(fhuA) Score=4.6 Pos=-62 [Nostoc sp. PCC 7120]
ATTAAGAATTATTAGCAGT
>alr2175(fhuE) Score=4.4 Pos=-120 [Nostoc sp. PCC 7120]
GTTAATAATCTTTCGCAAT
>alr2626(fhuE) Score=4.7 Pos=-106 [Nostoc sp. PCC 7120]
TTTGATATTAATTACTAAT
>all2586(fecC) Score=4.6 Pos=-100 [Nostoc sp. PCC 7120]
TCTTATATTAATTCTCAAT
>all2586(fecC) Score=5 Pos=-32 [Nostoc sp. PCC 7120]
AATGACTATATTTTTCAAT
>all2237(pchR) Score=5.1 Pos=-40 [Nostoc sp. PCC 7120]
TTTGAGAAAGTTTATCTAT
>all2235(fecB) Score=3.5 Pos=12 [Nostoc sp. PCC 7120]
TTTTATTATCATATTCAAC
>all1101(fhuA) Score=3.8 Pos=-238 [Nostoc sp. PCC 7120]
ATTAATATTAAATCCTAAT
>all4924(fhuE) Score=5.5 Pos=-32 [Nostoc sp. PCC 7120]
ATTGAGAATTTTTATCTAT
>all5047(exbB) Score=4.7 Pos=-102 [Nostoc sp. PCC 7120]
TTTGATAATAATTTGCATC
>alr2184(pchR) Score=5.1 Pos=-39 [Nostoc sp. PCC 7120]
ATTGAGAAATAATATTATT
>alr2185(fhuE) Score=4.3 Pos=-69 [Nostoc sp. PCC 7120]
TTTGCAAAAAATTATTAAC
>alr2211(fhuA) Score=2.9 Pos=-272 [Nostoc sp. PCC 7120]
ATTTATTTAATTATTTAGT
>all2580(pchR) Score=5.5 Pos=-48 [Nostoc sp. PCC 7120]
ATTGAGAAATAATATCATT
>alr2174(pchR) Score=5.6 Pos=-96 [Nostoc sp. PCC 7120]
ATTGATAAATACTATCATT
>Tery_2879(feoA) Score=3.5 Pos=-270 [Trichodesmium erythraeum IMS101]
TTTTCCAAATTTTTTCAGT

>Tery_2644(SYNPCC7002_A2351) Score=4.2 Pos=-122 [Trichodesmium erythraeum IMS101]
AATTAGATTAATTTTGAAT
>Tery_4282(dpsA) Score=2.4 Pos=-285 [Trichodesmium erythraeum IMS101]
ATTTTTTAACAATTGAAAT
>Tery_0513(psbC) Score=3.5 Pos=-195 [Trichodesmium erythraeum IMS101]
TTTACAAAAAATTTGTAAT
>Tery_1780(ctaA) Score=3.3 Pos=-167 [Trichodesmium erythraeum IMS101]
ATTAAAAAAAAATTTAAAA
>Tery_4773(SYNPCC7002_A0913) Score=2.4 Pos=-64 [Trichodesmium erythraeum IMS101]
TTTATGAAATATTTATGAT
>Tery_3224(chlH) Score=3 Pos=-65 [Trichodesmium erythraeum IMS101]
GTTTAAAATTTTTTGATAT
>Tery_1781(ctaB) Score=4.1 Pos=-41 [Trichodesmium erythraeum IMS101]
AATTATAACCAGTTTCAAT
>Tery_4669(psaA) Score=4 Pos=-80 [Trichodesmium erythraeum IMS101]
ATTGATAATTCTTTACAAC
>Tery_3222(afuC) Score=5.1 Pos=-94 [Trichodesmium erythraeum IMS101]
AATGAAAAAAATATTCAAT
>Tery_2878(feoB) Score=3 Pos=-43 [Trichodesmium erythraeum IMS101]
TATTACATAATTTTTATAT
>Tery_3377(sufA) Score=4.4 Pos=-97 [Trichodesmium erythraeum IMS101]
ATTAAGAATAAATTGCAAT
>Tery_1667(isiA) Score=3.2 Pos=-156 [Trichodesmium erythraeum IMS101]
TTTTATAATTTCTTTTAAA
>Tery_4504(fdx) Score=2.6 Pos=-35 [Trichodesmium erythraeum IMS101]
GTTAATATTTTTTTATAAC
>Tery_1958(fur) Score=4.7 Pos=-44 [Trichodesmium erythraeum IMS101]
ATTGTGTATCATTGTCAAT
>Tery_1780(ctaA) Score=3.5 Pos=-278 [Trichodesmium erythraeum IMS101]
ATTGTTAAAATTATGAAAT
>Synpcc7942_2601(ctaA) Score=4.3 Pos=-158 [Synechococcus elongatus PCC 7942]
GATGACAATATTTTTCGAT
>Synpcc7942_1406(afuC) Score=4.2 Pos=-81 [Synechococcus elongatus PCC 7942]
ATTGAGAATAAATCGCAGA
>Synpcc7942_0938(cce_4396) Score=4 Pos=-30 [Synechococcus elongatus PCC 7942]
AATAAAAAAGTTTCGTAAT
>Synpcc7942_1542(isiA) Score=5.2 Pos=-45 [Synechococcus elongatus PCC 7942]
ATTGAGAATTATTGTAAAT
>Synpcc7942_2049(psaA) Score=3.3 Pos=-296 [Synechococcus elongatus PCC 7942]
TCTTTGAATTATTGTTAAT
>PMT1340(ctaA) Score=2.6 Pos=-30 [Prochlorococcus marinus str. MIT 9313]
ATTTAAATTATTTTATTGT
>CYA_2647(psbD) Score=3 Pos=-106 [Synechococcus sp. JA-3-3Ab]
TTTATTAATTTTTCGTAAG
>CYA_2794(afuC) Score=3.8 Pos=-26 [Synechococcus sp. JA-3-3Ab]
AGTGAAAATCTTTTGCAAC
>CYA_0643(fdx) Score=2.8 Pos=-92 [Synechococcus sp. JA-3-3Ab]
ATTAAAAATATGATTTAAA
>gvip470(psaA) Score=2.2 Pos=-195 [Gloeobacter violaceus PCC 7421]
TTTTTAAAAATTGTTAAGT
>glr3733(perR) Score=4.2 Pos=-58 [Gloeobacter violaceus PCC 7421]
AATTAGATCTATTCTAAAT
>gll1014(afuA) Score=3.7 Pos=-64 [Gloeobacter violaceus PCC 7421]
ATTGACAATTAATTGCAGC
>null(chlL) Score=2.8 Pos=-211 [Thermosynechococcus elongatus BP-1]

TTTTGTATTTTTGTTTAAT
>null(psbA) Score=3.8 Pos=-135 [Thermosynechococcus elongatus BP-1]
ATTTAGTTTTACTAACAAT
>null(apcF) Score=3.1 Pos=-157 [Thermosynechococcus elongatus BP-1]
AATTATGAACTTTTGTAAT
>null(cce_4396) Score=4 Pos=-45 [Thermosynechococcus elongatus BP-1]
AATTAAAATTATATTCATC
>null(isiA) Score=5.5 Pos=-121 [Thermosynechococcus elongatus BP-1]
AATGCTATTAATTCTCAAT
>null(fur) Score=4.4 Pos=-59 [Thermosynechococcus elongatus BP-1]
CATTGTAATTATTCTCAAT

Supplementary multifasta 3, for NtcA boxes in cyanobacteria:

>SYNPCC7002_A2208(amt1) Score=3.7 Pos=-52 [Synechococcus sp. PCC 7002]
GGTTACTTCTGCTACC
>SYNPCC7002_A0076(SYNPCC7002_A0076) Score=4.2 Pos=-97 [Synechococcus sp. PCC 7002]
AGTATTGAAATATACA
>SYNPCC7002_A2395(SYNPCC7002_A2395) Score=4.5 Pos=-200 [Synechococcus sp. PCC 7002]
TGTTACTTAAATTACA
>SYNPCC7002_A2395(SYNPCC7002_A2395) Score=4.5 Pos=-92 [Synechococcus sp. PCC 7002]
TGTAGTAGACGTTACA
>SYNPCC7002_A1954(SYNPCC7002_A1954) Score=4.7 Pos=-243 [Synechococcus sp. PCC 7002]
TGTAACAGGAGTTACA
>SYNPCC7002_A0643(cynS) Score=4.5 Pos=-237 [Synechococcus sp. PCC 7002]
CGTATCTTTAATTACA
>SYNPCC7002_A0860(cynA) Score=4.1 Pos=-221 [Synechococcus sp. PCC 7002]
TGTAATTGATTTAACA
>SYNPCC7002_A0582(gifA) Score=5 Pos=-96 [Synechococcus sp. PCC 7002]
TGTATCAAATTTTACA
>SYNPCC7002_A1630(glnA) Score=3.5 Pos=-125 [Synechococcus sp. PCC 7002]
TGTGACCCAGACTACA
>SYNPCC7002_A1827(nirA) Score=4 Pos=-111 [Synechococcus sp. PCC 7002]
CGTAGTTAACACTACA
>SYNPCC7002_A0496(nrrA) Score=4.1 Pos=-75 [Synechococcus sp. PCC 7002]
CGTAACCACGGTTACA
>SYNPCC7002_A1675(ntcA) Score=3.9 Pos=-177 [Synechococcus sp. PCC 7002]
TGAAGCAAAAAGTACA
>SYNPCC7002_A1632(ntcB) Score=3.9 Pos=-87 [Synechococcus sp. PCC 7002]
TGTAACATCCGGAACA
>SYNPCC7002_A2101(SYNPCC7002_A2101) Score=5 Pos=67 [Synechococcus sp. PCC 7002]
TGTATCATTAAATACA
>SYNPCC7002_A0264(SYNPCC7002_A0264) Score=5 Pos=-29 [Synechococcus sp. PCC 7002]
TGTATCAAATACTACA
>SYNPCC7002_A0398(urtA) Score=4.1 Pos=-127 [Synechococcus sp. PCC 7002]
TGTATCAAGTGTCACA
>SYNPCC7002_A1443(nifJ) Score=4.1 Pos=-272 [Synechococcus sp. PCC 7002]
TTTATCATTTTCTACA
>sll0108(amt1) Score=4 Pos=-189 [Synechocystis sp. PCC 6803]
AGTAGTAAATCATACA
>sll0108(amt1) Score=4.4 Pos=-18 [Synechocystis sp. PCC 6803]
TGTAGATTAAAGTACA
>ssr1562(SYNPCC7002_A0076) Score=4.5 Pos=-78 [Synechocystis sp. PCC 6803]
TGTAATCGATAATACA
>slr1147(SYNPCC7002_A1954) Score=4.7 Pos=-214 [Synechocystis sp. PCC 6803]
TGTAATGATAGTTACA
>ssl1911(gifA) Score=5 Pos=-89 [Synechocystis sp. PCC 6803]
TGTATAAAATGTTACA
>slr1756(glnA) Score=4.3 Pos=-98 [Synechocystis sp. PCC 6803]
GGTAGCGAAAAATACA
>ssl0707(glnB) Score=3.7 Pos=-184 [Synechocystis sp. PCC 6803]

GGTACTGATTTTTACA
>slr0851(ndh) Score=4.1 Pos=-72 [Synechocystis sp. PCC 6803]
TGTAACAACCATTACC
>sll1450(nrtA) Score=3.9 Pos=-96 [Synechocystis sp. PCC 6803]
AGTTACAAACTATACA
>slr0898(nirA) Score=4.7 Pos=-74 [Synechocystis sp. PCC 6803]
TGTAATTTACGTTACA
>sll1330(nrrA) Score=4.6 Pos=-75 [Synechocystis sp. PCC 6803]
GGTAACTGTTGTTACA
>slr0653(rpoD) Score=3.9 Pos=-246 [Synechocystis sp. PCC 6803]
TGTTATCGAGGCTACA
>sll1515(gifB) Score=4.2 Pos=-119 [Synechocystis sp. PCC 6803]
CGTAAAAATGGATACA
>slr0447(urtA) Score=4.5 Pos=-211 [Synechocystis sp. PCC 6803]
GGTATCCTATGCTACA
>slr0009(rbcL) Score=4.3 Pos=-228 [Synechocystis sp. PCC 6803]
TGTAATTTAAAAAACA
>sll0741(nifJ) Score=3.3 Pos=-181 [Synechocystis sp. PCC 6803]
AGAATCAACAAAAACA
>sll1733(ndhD) Score=4 Pos=-252 [Synechocystis sp. PCC 6803]
TGTAACAATTTTTAGT
>sll1732(ndhF) Score=4 Pos=-244 [Synechocystis sp. PCC 6803]
TGTAACAAATAACACT
>sll1712(hanA) Score=3.8 Pos=-264 [Synechocystis sp. PCC 6803]
GGTAGTTTTTGAAACT
>cce_3261(amt1) Score=3.5 Pos=-152 [Cyanothece sp. ATCC 51142]
TGTTAAGGTTGTAACA
>cce_0537(SYNPCC7002_A0076) Score=4 Pos=-137 [Cyanothece sp. ATCC 51142]
TGTAAACTATGATATA
>cce_0537(SYNPCC7002_A0076) Score=4.8 Pos=-93 [Cyanothece sp. ATCC 51142]
TGTAGTCTTTGTTACA
>cce_4355(SYNPCC7002_A0077) Score=4.7 Pos=-172 [Cyanothece sp. ATCC 51142]
TGTTACAATAGCTACA
>cce_2729(SYNPCC7002_A2395) Score=4 Pos=-148 [Cyanothece sp. ATCC 51142]
TGTAACATATGATAAC
>cce_2729(SYNPCC7002_A2395) Score=4.6 Pos=-87 [Cyanothece sp. ATCC 51142]
TGTAATATTGATTACA
>cce_3327(SYNPCC7002_A1954) Score=4.7 Pos=-210 [Cyanothece sp. ATCC 51142]
TGTAGTCATAGTTACA
>cce_3327(SYNPCC7002_A1954) Score=4 Pos=-46 [Cyanothece sp. ATCC 51142]
TTTATTTTCTGTTACA
>cce_3797(cce_3797) Score=4.9 Pos=-70 [Cyanothece sp. ATCC 51142]
TGTAGCTTAATATACA
>cce_0259(gifA) Score=5.2 Pos=-95 [Cyanothece sp. ATCC 51142]
TGTAGCAAATGTTACA
>cce_4432(glnA) Score=4.5 Pos=-89 [Cyanothece sp. ATCC 51142]
AGTATCCAATTATACA
>cce_4432(glnA) Score=4 Pos=-30 [Cyanothece sp. ATCC 51142]
TGTATATATTGAAACC
>cce_1063(hupS) Score=4.8 Pos=-204 [Cyanothece sp. ATCC 51142]
TGTAAAATTTAATACA
>cce_1063(hupS) Score=3.8 Pos=-159 [Cyanothece sp. ATCC 51142]
GGTAATAATTAATAGA
>cce_0624(ndh) Score=4.7 Pos=-47 [Cyanothece sp. ATCC 51142]
TGTAACTGTAGTTACT

>cce_1223(nirA) Score=4.7 Pos=-76 [Cyanothece sp. ATCC 51142]
TGTTACATTAGCTACA
>cce_1808(nrrA) Score=4.3 Pos=-76 [Cyanothece sp. ATCC 51142]
CGTAGCCGATGTTACA
>cce_0198(ntcB) Score=4.2 Pos=-73 [Cyanothece sp. ATCC 51142]
AGTATCAATTTCTACG
>cce_3797(SYNPCC7002_A2101) Score=4.9 Pos=-70 [Cyanothece sp. ATCC 51142]
TGTAGCTTAATATACA
>cce_0267(psbA3) Score=4.4 Pos=-58 [Cyanothece sp. ATCC 51142]
GGTAATCATTGATACA
>cce_0875(rpoD) Score=4.3 Pos=-232 [Cyanothece sp. ATCC 51142]
AGTAATCAAGGCTACA
>cce_2638(gifB) Score=5.2 Pos=-144 [Cyanothece sp. ATCC 51142]
TGTAACAATAGATACA
>cce_1944(apcF) Score=3.8 Pos=-177 [Cyanothece sp. ATCC 51142]
TGTGATAAGAAATACA
>cce_2390(trxQ) Score=3.7 Pos=-332 [Cyanothece sp. ATCC 51142]
AATAGCTATAATTACT
>cce_3633(psbZ) Score=4.2 Pos=-20 [Cyanothece sp. ATCC 51142]
TGTAACTGTATTAACA
>cce_0953(nifJ) Score=3.8 Pos=-52 [Cyanothece sp. ATCC 51142]
TGCAACAAAAATTACC
>PCC8801_1229(amt1) Score=5 Pos=-264 [Cyanothece sp. PCC 8801]
TGTATTTTTTGATACA
>PCC8801_1229(amt1) Score=4.1 Pos=-229 [Cyanothece sp. PCC 8801]
AGTAATAAAAAAAACA
>PCC8801_3101(SYNPCC7002_A0076) Score=4.6 Pos=-95 [Cyanothece sp. PCC 8801]
TGTATTGGTTGTTACA
>PCC8801_2066(SYNPCC7002_A2395) Score=4.8 Pos=-53 [Cyanothece sp. PCC 8801]
TGTAATATTAATTACA
>PCC8801_3053(SYNPCC7002_A1954) Score=4.8 Pos=-198 [Cyanothece sp. PCC 8801]
TGTAATCATAGTTACA
>PCC8801_3848(cce_3797) Score=5.1 Pos=-165 [Cyanothece sp. PCC 8801]
TGTAACTTTAGATACA
>PCC8801_2371(gifA) Score=4.9 Pos=-94 [Cyanothece sp. PCC 8801]
TGTATAAATTGCTACA
>PCC8801_0808(glnA) Score=4 Pos=-92 [Cyanothece sp. PCC 8801]
CGTATCATGGTATACA
>PCC8801_3212(hupS) Score=4.3 Pos=-127 [Cyanothece sp. PCC 8801]
TGTAACAACAATAACA
>PCC8801_3485(ndh) Score=4.6 Pos=-117 [Cyanothece sp. PCC 8801]
TGTAACCATAGCTACT
>PCC8801_3485(ndh) Score=3.8 Pos=-92 [Cyanothece sp. PCC 8801]
CGTAATTGGTTTTACA
>PCC8801_4396(nrtA) Score=4.6 Pos=-184 [Cyanothece sp. PCC 8801]
TGTAGCAAATAATACG
>PCC8801_4396(nrtA) Score=3.9 Pos=-106 [Cyanothece sp. PCC 8801]
AGTATTTATAGACACA
>PCC8801_2468(nirA) Score=4.6 Pos=-75 [Cyanothece sp. PCC 8801]
TGTTACAATTAATACA
>PCC8801_2463(narB) Score=4.4 Pos=-165 [Cyanothece sp. PCC 8801]
TGTATTACCAAATACA
>PCC8801_3375(nrrA) Score=4.5 Pos=-76 [Cyanothece sp. PCC 8801]
CGTAGCAGATGTTACA
>PCC8801_2329(ntcB) Score=4.7 Pos=-80 [Cyanothece sp. PCC 8801]

TGTATCAAATTCTACT
>PCC8801_3848(SYNPCC7002_A2101) Score=5.1 Pos=-165 [Cyanothece sp. PCC 8801]
TGTAACTTTAGATACA
>PCC8801_3216(psbA3) Score=4.4 Pos=-59 [Cyanothece sp. PCC 8801]
AGTAGTTATTGCTACT
>PCC8801_0154(rpoD) Score=4.4 Pos=-257 [Cyanothece sp. PCC 8801]
TGTAATCGAGGCTACA
>PCC8801_0807(apcF) Score=4 Pos=-251 [Cyanothece sp. PCC 8801]
TGTATACCATGATACG
>PCC8801_0870(fur) Score=3.4 Pos=-380 [Cyanothece sp. PCC 8801]
TGTTTATGCTAAAACA
>PCC8801_0567(coxB) Score=3.8 Pos=-148 [Cyanothece sp. PCC 8801]
TTTATTATTTGTTACC
>PCC8801_3918(nifJ) Score=3.5 Pos=-265 [Cyanothece sp. PCC 8801]
AGTAACCATAATTATT
>PCC8801_4247(ndhF) Score=3.6 Pos=-238 [Cyanothece sp. PCC 8801]
CGTATTATGAGTTAGA
>Cyan7425_0782(amt1) Score=4.4 Pos=-228 [Cyanothece sp. PCC 7425]
TGTATAACGTGATACA
>Cyan7425_0729(SYNPCC7002_A0076) Score=4.3 Pos=-128 [Cyanothece sp. PCC 7425]
TGTAACATTTGTTATT
>Cyan7425_0729(SYNPCC7002_A0076) Score=4.1 Pos=-71 [Cyanothece sp. PCC 7425]
AGTATTGGTGGATACA
>Cyan7425_0833(SYNPCC7002_A2395) Score=3.7 Pos=-38 [Cyanothece sp. PCC 7425]
AGTAGTTTAATAAACT
>Cyan7425_0732(cce_3797) Score=5.2 Pos=-33 [Cyanothece sp. PCC 7425]
TGTATCATAAGCTACA
>Cyan7425_1839(glnA) Score=4.9 Pos=-221 [Cyanothece sp. PCC 7425]
TGTAACTCATGATACA
>Cyan7425_4042(Cyan7425_4042) Score=4.4 Pos=-151 [Cyanothece sp. PCC 7425]
AGTAGCTCGTGATACA
>Cyan7425_4573(nirA) Score=4.5 Pos=-123 [Cyanothece sp. PCC 7425]
TGTAACTTAATATACC
>Cyan7425_0851(nrrA) Score=4.1 Pos=-82 [Cyanothece sp. PCC 7425]
AGTAGCTAGAGTTACG
>Cyan7425_0755(ntcA) Score=4.8 Pos=-173 [Cyanothece sp. PCC 7425]
TGTATCCGATGATACA
>Cyan7425_1599(ntcB) Score=4.6 Pos=-78 [Cyanothece sp. PCC 7425]
TGTATCACTATCTACA
>Cyan7425_0732(SYNPCC7002_A2101) Score=5.2 Pos=-33 [Cyanothece sp. PCC 7425]
TGTATCATAAGCTACA
>Cyan7425_1838(apcF) Score=4.9 Pos=-281 [Cyanothece sp. PCC 7425]
TGTATCATGAGTTACA
>Cyan7425_4369(nifJ) Score=3.1 Pos=-120 [Cyanothece sp. PCC 7425]
TCTAACGTATTCTCCA
>Cyan7425_2260(urtA) Score=3.7 Pos=-148 [Cyanothece sp. PCC 7425]
TGTATAATAGATCACA
>Cyan7425_1393(gifB) Score=4.7 Pos=-158 [Cyanothece sp. PCC 7425]
GGTATCATATAATACA
>MAE_62530(icd) Score=3.7 Pos=-30 [Microcystis aeruginosa NIES-843]
CGTATAGATAAATACT
>MAE_40010(amt1) Score=4.6 Pos=-213 [Microcystis aeruginosa NIES-843]
TGTATCAACGAATACA
>MAE_39690(SYNPCC7002_A0076) Score=4.3 Pos=-99 [Microcystis aeruginosa NIES-843]
TGTAATCGTCGATACA

>MAE_38700(SYNPCC7002_A2395) Score=4.2 Pos=-87 [Microcystis aeruginosa NIES-843]
TGTAATCATCTTTACA
>MAE_14410(SYNPCC7002_A1954) Score=3.7 Pos=-75 [Microcystis aeruginosa NIES-843]
TGTAAACTGGGAAACA
>MAE_14840(cce_3797) Score=4.9 Pos=-38 [Microcystis aeruginosa NIES-843]
TGTAAATTTAGATACA
>MAE_49490(gifA) Score=4.9 Pos=-42 [Microcystis aeruginosa NIES-843]
TGTAAAAATAGATACA
>MAE_19270(glnA) Score=4.7 Pos=-92 [Microcystis aeruginosa NIES-843]
TGTATCGAAAAATACA
>MAE_09050(glnN) Score=4.7 Pos=-244 [Microcystis aeruginosa NIES-843]
TGTAATGTTTGATACA
>MAE_18410(nirA) Score=4.1 Pos=-190 [Microcystis aeruginosa NIES-843]
TGTTACAGACAATACA
>MAE_14810(MAE_14810) Score=4.2 Pos=-177 [Microcystis aeruginosa NIES-843]
TGTAACAATCTATACG
>MAE_53960(narB) Score=4.8 Pos=-88 [Microcystis aeruginosa NIES-843]
TGTATTAATAACTACA
>MAE_09380(ntcB) Score=4.1 Pos=-80 [Microcystis aeruginosa NIES-843]
GGTAGCAATTTCTACC
>MAE_14840(SYNPCC7002_A2101) Score=4.9 Pos=-38 [Microcystis aeruginosa NIES-843]
TGTAAATTTAGATACA
>MAE_54470(rpoD) Score=4.9 Pos=-126 [Microcystis aeruginosa NIES-843]
TGTAATTAAAGCTACA
>MAE_18580(SYNPCC7002_A0264) Score=4.8 Pos=-68 [Microcystis aeruginosa NIES-843]
TGTATCTTTAGCTACT
>MAE_06220(urtA) Score=4.6 Pos=-140 [Microcystis aeruginosa NIES-843]
AGTAGCGAAAGTTACA
>MAE_06180(urtE) Score=3.8 Pos=-8 [Microcystis aeruginosa NIES-843]
GGTTGACAATGTTACA
>MAE_37080(fur) Score=3.7 Pos=-118 [Microcystis aeruginosa NIES-843]
AGTAATTTTTCATACT
>MAE_22930(coxB) Score=3.8 Pos=-141 [Microcystis aeruginosa NIES-843]
AGTTATTTTAAATACT
>MAE_06220(urtA) Score=4.7 Pos=-170 [Microcystis aeruginosa NIES-843]
AGTAGCAAAAATTACA
>all4968(gor) Score=4.1 Pos=-48 [Nostoc sp. PCC 7120]
AATAACAACTGTTACA
>alr1827(icd) Score=3.4 Pos=-6 [Nostoc sp. PCC 7120]
TGAAATATGTACAACA
>alr0992(amt1) Score=4.7 Pos=-50 [Nostoc sp. PCC 7120]
TGTAACTAAGTATACA
>alr0990(amt1) Score=4.7 Pos=-250 [Nostoc sp. PCC 7120]
TGTATTAACTAATACA
>asr0064(SYNPCC7002_A0076) Score=3.7 Pos=-107 [Nostoc sp. PCC 7120]
CGTATAGCAAAATACA
>alr4308(SYNPCC7002_A2395) Score=3.9 Pos=-64 [Nostoc sp. PCC 7120]
GGTAGTCGGAGTTACA
>alr3982(SYNPCC7002_A1954) Score=4.6 Pos=-250 [Nostoc sp. PCC 7120]
TGTAGTGATAGCTACA
>asl2329(gifA) Score=4.7 Pos=-78 [Nostoc sp. PCC 7120]
CGTAGCATAAGATACA
>alr2328(glnA) Score=4.8 Pos=-142 [Nostoc sp. PCC 7120]
TGTAACAAAGACTACA
>all2319(glnB) Score=3.8 Pos=-214 [Nostoc sp. PCC 7120]

AGTTACACAGACTACA
>all1127(ndh) Score=4.7 Pos=-146 [Nostoc sp. PCC 7120]
TGTAGCTTAAATTACT
>all4312(nrrA) Score=4.5 Pos=-75 [Nostoc sp. PCC 7120]
AGTAACAAAGACTACA
>alr4392(ntcA) Score=3.7 Pos=-151 [Nostoc sp. PCC 7120]
GGTATCATTATGAACA
>alr4392(ntcA) Score=3.7 Pos=-111 [Nostoc sp. PCC 7120]
AGTATAGGAAAGTACA
>all0602(ntcB) Score=4.5 Pos=-80 [Nostoc sp. PCC 7120]
TGTAACAAAATCTACC
>all0602(ntcB) Score=4.7 Pos=-21 [Nostoc sp. PCC 7120]
TGTAATTAAGGCTACA
>all1327(SYNPCC7002_A0264) Score=4.3 Pos=-52 [Nostoc sp. PCC 7120]
CGTAGTGTATGTTACA
>all2327(apcF) Score=4.8 Pos=-233 [Nostoc sp. PCC 7120]
TGTAGTCTTTGTTACA
>all1691(fur) Score=4.1 Pos=-218 [Nostoc sp. PCC 7120]
TGTAGCTTGAGATTCA
>alr1690(alr1690) Score=3.6 Pos=-91 [Nostoc sp. PCC 7120]
TGTGAATATAGAAACA
>alr2514(coxB) Score=3.5 Pos=-291 [Nostoc sp. PCC 7120]
AGAAGTAGAAGCTACT
>all4001(isiA) Score=3.7 Pos=-37 [Nostoc sp. PCC 7120]
AGTATTTCTGGCAACA
>alr1524(rbcL) Score=3.6 Pos=-32 [Nostoc sp. PCC 7120]
AGTAAAAAGAGTGACA
>alr4344(gltS) Score=3.5 Pos=-265 [Nostoc sp. PCC 7120]
TGGAAAAATTAAAACA
>all1258(psbZ) Score=3.4 Pos=-161 [Nostoc sp. PCC 7120]
TGTTACAAAAATTAGG
>alr1911(nifJ) Score=3.2 Pos=-281 [Nostoc sp. PCC 7120]
TGAATTTGATGTAACC
>alr4156(ndhF) Score=4 Pos=-260 [Nostoc sp. PCC 7120]
TGTCTTATAAAATACA
>alr0052(trxA) Score=3.7 Pos=17 [Nostoc sp. PCC 7120]
AGTTACAGATTCTACT
>asr3935(hanA) Score=3.6 Pos=-279 [Nostoc sp. PCC 7120]
GGTATTTTTTCCTACT
>all1951(urtA) Score=4.3 Pos=-116 [Nostoc sp. PCC 7120]
AGTATCAAAAATAACA
>Tery_4477(amt1) Score=4.9 Pos=-83 [Trichodesmium erythraeum IMS101]
AGTAGCATTTGATACA
>Tery_2891(SYNPCC7002_A0076) Score=4.1 Pos=-191 [Trichodesmium erythraeum IMS101]
TGTAATTTAGACAACA
>Tery_2891(SYNPCC7002_A0076) Score=4.6 Pos=-110 [Trichodesmium erythraeum IMS101]
TGTAACTATATATACT
>Tery_0672(SYNPCC7002_A2395) Score=4.3 Pos=-94 [Trichodesmium erythraeum IMS101]
AGTATTCAAAATTACA
>Tery_3912(SYNPCC7002_A1954) Score=4.3 Pos=-156 [Trichodesmium erythraeum IMS101]
TGTAGTTATGGCTACC
>Tery_3834(glnA) Score=4 Pos=-250 [Trichodesmium erythraeum IMS101]
CGTAACACCCGATACA
>Tery_3369(hupS) Score=4.3 Pos=-188 [Trichodesmium erythraeum IMS101]
AGTATCAAAAATAACA

>Tery_3368(hupL) Score=4.1 Pos=-64 [Trichodesmium erythraeum IMS101]
TGTAGCTACTGATAAA
>Tery_1068(nirA) Score=3.8 Pos=-276 [Trichodesmium erythraeum IMS101]
TGTATTTTTATATAGT
>Tery_1068(nirA) Score=4.1 Pos=-199 [Trichodesmium erythraeum IMS101]
TGTTATATAAGCTACG
>Tery_0675(nrrA) Score=4.7 Pos=-89 [Trichodesmium erythraeum IMS101]
AGTAGCTTCTGTTACA
>Tery_4333(ntcB) Score=4 Pos=-27 [Trichodesmium erythraeum IMS101]
TGTATAAAATAACACA
>Tery_5068(rpoD) Score=4 Pos=-199 [Trichodesmium erythraeum IMS101]
TGTAAAATTTTGTACC
>Tery_5068(rpoD) Score=3.7 Pos=-46 [Trichodesmium erythraeum IMS101]
TGTAAAATAATTTATT
>Tery_3029(gifB) Score=3.8 Pos=-349 [Trichodesmium erythraeum IMS101]
TGTATCTGATGTTAGG
>Tery_1958(fur) Score=3.5 Pos=-40 [Trichodesmium erythraeum IMS101]
TGTATCATTGTCAATA
>Tery_4410(rbcL) Score=3.4 Pos=-293 [Trichodesmium erythraeum IMS101]
GATAACTATTACTACG
>Tery_1667(isiA) Score=3.6 Pos=-134 [Trichodesmium erythraeum IMS101]
AGTAATTGATCATACT
>Tery_0466(gltS) Score=4.2 Pos=-228 [Trichodesmium erythraeum IMS101]
TGTATCCTAGGCAACA
>Tery_0130(urtA) Score=3.8 Pos=-390 [Trichodesmium erythraeum IMS101]
AGTTACAAAAAAAACA
>Tery_4747(gor) Score=3.9 Pos=-208 [Trichodesmium erythraeum IMS101]
TGTAGTCGTAGATATA
>Tery_4747(gor) Score=4.7 Pos=-120 [Trichodesmium erythraeum IMS101]
TGTAACTAATTATACT
>Synpcc7942_0442(amt1) Score=4.1 Pos=-151 [Synechococcus elongatus PCC 7942]
TGTTACATCGATTACA
>Synpcc7942_1845(cce_3797) Score=4.3 Pos=-27 [Synechococcus elongatus PCC 7942]
TGTATCCGTTGCTACC
>Synpcc7942_2107(cynA) Score=4.4 Pos=-67 [Synechococcus elongatus PCC 7942]
TGTAACGACGGCTACA
>Synpcc7942_2156(glnA) Score=4.8 Pos=-194 [Synechococcus elongatus PCC 7942]
TGTATCAGCTGTTACA
>Synpcc7942_0321(glnB) Score=4.8 Pos=-103 [Synechococcus elongatus PCC 7942]
TGTAGCAGTAACTACA
>Synpcc7942_1240(nirA) Score=4.9 Pos=-220 [Synechococcus elongatus PCC 7942]
TGTAGCAATTGCTACT
>Synpcc7942_1240(nirA) Score=4.3 Pos=-149 [Synechococcus elongatus PCC 7942]
AGTATCAATGATTACT
>Synpcc7942_1240(nirA) Score=4.4 Pos=-80 [Synechococcus elongatus PCC 7942]
TGTAGTTTCTGTTACC
>Synpcc7942_0127(ntcA) Score=4.7 Pos=-157 [Synechococcus elongatus PCC 7942]
AGTAGCAGTTGCTACA
>Synpcc7942_1241(nirB) Score=4.3 Pos=-153 [Synechococcus elongatus PCC 7942]
AGTAATCATTGATACT
>Synpcc7942_1241(nirB) Score=4.9 Pos=-82 [Synechococcus elongatus PCC 7942]
AGTAGCAATTGCTACA
>Synpcc7942_1845(SYNPCC7002_A2101) Score=4.3 Pos=-27 [Synechococcus elongatus PCC 7942]
TGTATCCGTTGCTACC

>Synpcc7942_2158(apcF) Score=4.8 Pos=-214 [Synechococcus elongatus PCC 7942]
TGTAACAGCTGATACA
>PMT1853(amt1) Score=3.8 Pos=-160 [Prochlorococcus marinus str. MIT 9313]
TGTAACAAAATGAACT
>PMT0601(glnA) Score=4.2 Pos=-79 [Prochlorococcus marinus str. MIT 9313]
GGTACCTGTTGCTACA
>PMT2229(urtA) Score=4 Pos=-146 [Prochlorococcus marinus str. MIT 9313]
TGTTGCAACAGCTACC
>PMT2229(urtA) Score=4.7 Pos=-95 [Prochlorococcus marinus str. MIT 9313]
TGTATCATTCACTACA
>CYA_0030(fur) Score=3.6 Pos=-66 [Synechococcus sp. JA-3-3Ab]
GCTATTATCAATTACA
>CYA_0570(SYNPCC7002_A2395) Score=3.9 Pos=-139 [Synechococcus sp. JA-3-3Ab]
GGTAAGATAAAATACA
>CYA_0570(SYNPCC7002_A2395) Score=4.2 Pos=-81 [Synechococcus sp. JA-3-3Ab]
CGTATCAAAGGCTACT
>CYA_1363(glnA) Score=4.3 Pos=-138 [Synechococcus sp. JA-3-3Ab]
CGTATCAGAAACTACA
>CYA_1508(glnB) Score=4.5 Pos=-74 [Synechococcus sp. JA-3-3Ab]
TGTATCCGTTTTTACA
>CYA_0612(nirA) Score=3.9 Pos=-72 [Synechococcus sp. JA-3-3Ab]
TGTAACTGCCAATACC
>CYA_1799(ntcA) Score=4.7 Pos=-181 [Synechococcus sp. JA-3-3Ab]
TGTAGCTCCTGCTACA
>CYA_0984(ntcB) Score=4.2 Pos=-89 [Synechococcus sp. JA-3-3Ab]
TGTAACGGTGTTTACA
>CYA_1920(urtE) Score=3.9 Pos=-248 [Synechococcus sp. JA-3-3Ab]
AGAAACAGAAGCTACA
>CYA_1924(urtA) Score=3.7 Pos=-272 [Synechococcus sp. JA-3-3Ab]
GGTATCCCGCGATACA
>SYNW0253(amt1) Score=4.2 Pos=-100 [Synechococcus sp. WH 8102]
TGTATCAATTAACACA
>SYNW0246(SYNPCC7002_A1954) Score=4.1 Pos=-143 [Synechococcus sp. WH 8102]
TGTAACTTTGGTTGCA
>SYNW2487(cynA) Score=4.1 Pos=-103 [Synechococcus sp. WH 8102]
TGTAGCAAGTGAGACA
>SYNW2487(cynA) Score=4.5 Pos=-66 [Synechococcus sp. WH 8102]
AGTATCACCTGATACA
>SYNW2442(urtA) Score=4 Pos=-118 [Synechococcus sp. WH 8102]
TGTTGCAACAGCTACC
>gvip146(glnA) Score=4 Pos=-116 [Gloeobacter violaceus PCC 7421]
CGTATCTCAGACTACA
>gvip212(nirA) Score=4 Pos=-146 [Gloeobacter violaceus PCC 7421]
TGTATCTGGGGTTACG
>gvip454(ntcA) Score=3.9 Pos=-77 [Gloeobacter violaceus PCC 7421]
AGTAGTCCCTAATACA
>gvip213(ntcB) Score=4 Pos=-46 [Gloeobacter violaceus PCC 7421]
CGTAACCCCAGATACA
>glr3304(fur) Score=3.9 Pos=-116 [Gloeobacter violaceus PCC 7421]
TGTAACAAATTGTAGA
>gll0880(trxA) Score=3.6 Pos=-379 [Gloeobacter violaceus PCC 7421]
TGTATCTAGAACTAGC
>null(gifB) Score=4.4 Pos=-175 [Thermosynechococcus elongatus BP-1]
TGTTAAATTAGATACA
>amt1(amt1) Score=4.4 Pos=-190 [Thermosynechococcus elongatus BP-1]

TGTATAACGTGATACA
>tsr2448(SYNPCC7002_A0076) Score=4.1 Pos=-63 [Thermosynechococcus elongatus BP-1]
AGTATTGGATATTACA
>tlr1468(SYNPCC7002_A2395) Score=4.3 Pos=-85 [Thermosynechococcus elongatus BP-1]
TGTAGTTGAGTTTACA
>tlr0194(tlr0194) Score=4.2 Pos=-111 [Thermosynechococcus elongatus BP-1]
TGTAACCTATCTTACA
>tll0591(glnB) Score=5.1 Pos=-80 [Thermosynechococcus elongatus BP-1]
TGTAGCTATTGCTACA
>tlr1349(nirA) Score=4.6 Pos=-82 [Thermosynechococcus elongatus BP-1]
AGTAGCAAAATTTACA
>tlr1330(nrrA) Score=4 Pos=-138 [Thermosynechococcus elongatus BP-1]
AGTATTATTCGTTACG
>null(ntcB) Score=3.9 Pos=-122 [Thermosynechococcus elongatus BP-1]
TGTATCAATCAACACA
>tlr1120(urtA) Score=4 Pos=-81 [Thermosynechococcus elongatus BP-1]
TGTAGCCTAGCATACA
>tlr2286(trxQ) Score=4 Pos=-130 [Thermosynechococcus elongatus BP-1]
AGTAGCAATGACAACA
>null(isiA) Score=3.8 Pos=-195 [Thermosynechococcus elongatus BP-1]
AGTTTCTGGAAATACA
>null(ndhF) Score=3.6 Pos=-220 [Thermosynechococcus elongatus BP-1]
GGTAACTTTTGCTATG
>null(gifB) Score=4.4 Pos=-146 [Thermosynechococcus elongatus BP-1]
TGTTAAATTAGATACA

Supplementary position frequency matrix (PFM 1) for PHO boxes in cyanobacteria:

A: 20	10	4	61	66	21	14	23	0	0	0	7
	18	3	43	61	24	4	16	0	0	0	12
	6	2	49	63	17	11	16				
C: 20	1	0	0	1	28	42	8	0	0	0	28
	2	2	0	1	30	38	6	0	0	0	34
	5	3	3	0	36	31	11				
G: 14	1	0	4	4	6	5	2	0	0	0	22
	1	1	2	7	2	7	4	0	0	0	18
	0	0	3	6	6	2	2				
N: 0	0	0	0	0	0	0	0	77	77	77	0
	0	0	0	0	0	0	0	77	77	77	0
	0	0	0	0	0	0	0				
T: 23	65	73	12	6	22	16	44	0	0	0	20
	56	71	32	8	21	28	51	0	0	0	13
	66	72	22	8	18	33	48				

Supplementary position frequency matrix (PFM 2) for Fur boxes in cyanobacteria:

A: 100	29	3	45	101	34	134	129	64	58	92	21
	13	26	11	26	147	127	18				
C: 1	5	1	4	18	9	8	1	6	18	2	18
	0	38	5	63	0	1	30				
G: 22	3	0	67	10	52	2	2	0	7	2	10
	2	10	41	2	4	25	2				
T: 44	130	163	51	38	72	23	35	97	84	71	118
	152	93	110	76	16	14	117				

Supplementary position frequency matrix (PFM 3) for NtcA boxes in cyanobacteria:

A: 47	3	6	201	99	29	108	94	90	88	71	88
	27	225	2	168							
C: 18	2	1	1	2	131	36	18	28	16	6	61
	7	1	211	19							
G: 20	220	1	3	52	1	19	43	21	33	113	8
	2	1	8	12							
T: 143	3	220	23	75	67	65	73	89	91	38	71
	192	1	7	29							

Supplementary Table 2. Pho regulon prediction. PHO boxes sequences, their position in the genome, orientation, the genes that are controlling (the Pho regulon), locus tag from these genes, the distance of the box from the gene and the gen function from single-letter of functional (COGs) are presented.

PHO boxes sequences	Position	Orientation	Pho regulon (genes)	Locus_tag	Distance	COG
**GATATCCTTAACTATAATTTCTAATAACTT**	37692	(+)	AbgT family transporter	PCC7418_RS00155	-342	H
**TTTAATTTCATCCTAGTTTAGGCTTAAAGT**	41629	(+)	serine hydrolase-HP-ABC transporter ATP-binding protein/permease	PCC7418_RS00180/RS00185/RS00190	-145	V, S, S
**AATAAAACTTAATTAAAGCCATCTTAACCT**	48034	(+)	PstS family phosphate ABC transporter substrate-binding protein (*pstS*)	PCC7418_RS00210	-51	P
**AAGAAATAATGTGTTAAAGTTAATATAAAT**	54813	(-)	PAS domain-containing protein response regulator	PCC7418_RS00225/RS00230	-315	T, T
**GAGTAAACCAAAGGCTAAATTAACAATATT**	59558	(-)	thioredoxin	PCC7418_RS00250	-262	O
**TCTTAAAGTTACAGTTAATGAAAGATTATA**	61353	(-)	mechanosensitive ion channel	PCC7418_RS00255	-316	M
**GAGTTATCGGAAGAAAAAGATGAGTATAAT**	63341	(-)	recombination protein RecR-tetratricopeptide repeat protein	PCC7418_RS00265/RS00270	-47	S, L
**ATTTAATTACTTAGAAACTCTGCTTAACAA**	73689	(+)	IS200/IS605 family element transposase accessoryprotein (*tnpB*)	PCC7418_RS00335	-175	L
**TTTAAACTCAGCATTATCTTTAGATTTGAC**	119529	(+)	RMD1 family protein-hypothetical protein (HP)-asparagine synthase	PCC7418_RS00490/RS00495/RS00500	-519	S, S, L, E
**TTTAGCCTCTTTCTTACCCTTTTTTAACAA**	163587	(+)	universal stress protein	PCC7418_RS00715	-112	T
**TTTTGTGTTACTTTAACCTTCCTTTTAGCA**	168098	(+)	ABC transporter substrate binding protein	PCC7418_RS00750	+34	G
**AATTTAAAATGAAATTATGCAATCAAAAAT**	187427	(-)	HP-glycoside hydrolase	PCC7418_RS00830/RS00835	-221	G, S
**AATTAAACCTAAAATAGTTTCAAGGATAAT**	192110	(-)	transposase	PCC7418_RS00850	-615	S
**TCATTAAGTTTTTGTTAAAGGAAGGTTATC**	193598	(-)	phosphoribosylformylglycinamidine cyclo-ligase	PCC7418_RS00865	-47	F
**ATTAAGTTTTTGTTAAAGGAAGGTTATCAT**	193600	(+)	septal ring lytic transglycosylase RlpA family protein	PCC7418_RS00870	-256	M
**TTGTAAGGCAGAATCAAAATTCAGTTTATC**	226059	(-)	HP	PCC7418_RS01010	-253	O
**CTTAATGTGATTTAAAATACCATTTAAGTT**	226508	(+)	HP	PCC7418_RS01025	-223	S
**TTTAATATTAGTTTAATATTAGTTTAAGTA**	227609	(+)	Nif11 like leader peptide family natural productprecursor	PCC7418_RS01030	-115	S

**CTTAGCCCCAGCTTTAGTTAATATTAATAC**	299142	(+)	archease RtcB family protein-phosphoribosyltransferase	PCC7418_0261/RS01330/RS01335	-274	S, L, S
**GTTTACTTAATTTTTATTACAAAATAAGAT**	323385	(+)	B12-binding domain-containing radical SAM protein	PCC7418_RS01445	-99	C
**AATTTAAGACCTAGTAATCAACACGAAATT**	345451	(-)	iron uptake porin	PCC7418_RS01525	-15	M
**TTTAGTAATCGCTTAACCTTTTCTTAACTT**	351477	(+)	thylakoid membrane photosystem I accumulation factor	PCC7418_RS01570	-439	CO
**GTTTATCAACTTTTTACATAAGATTTAGCA**	362063	(+)	NAD(P)H dependent glicerol 3 phosphate dehydrogenase	PCC7418_RS01620	-78	I
**AGTCAAAAAAATATTAAATCAATCATTAAT**	362100	(-)	NUDIX hydrolase	PCC7418_RS01615	-293	F
**ATTAAACTTTTTAATATCAGAGTATAATCA**	397041	(+)	HP-NblA/ycf18 family protein	PCC7418_RS01780/RS01785	-106	S, S
**ATTATTTTAATGTTTTTCAGTCTTTTTATT**	402390	(+)	3-isopropylmalate dehydrogenase (*leuB*)	PCC7418_RS01815	-308	CE
**AGATTAAAGCCAATTTATGAAGAGGTTAAA**	435783	(-)	bifunctional aminoglycoside phosphotransferase ATP binding protein	PCC7418_RS01975	-39	S
**TTTTATTTATATTTACACTGCCTTTAACGA**	440704	(+)	trypsin-like peptidase domain containing protein	PCC7418_RS02005	-238	O
**AATTAAAGTTATGATAAAAGCGATCTTAAA**	449261	(-)	SIMPL domain containing protein	PCC7418_RS02035	-714	S
**AGGTTAAATATGGTTCAAAAACTATTCAAA**	449976	(-)	uroporphyrinogen III C-methyltransferase	PCC7418_RS02045	0	H
**GTTTAAGTTTCGATAAAATTTCGTTATCAT**	456308	(+)	phosphomannose isomerase type II C-terminal cupin domain-lecithin retinol acyltransferase family protein	PCC7418_RS02090/RS02095	-115	G, KT
**AAATGAAGAAGAAATTATGCTGATGCCAAA**	456629	(-)	branched-chain amino acid ABC transporter permease	PCC7418_RS02085	-325	E
**AAGTAAAGTCTTTTTAATAAAAATTTAATA**	524155	(-)	response regulator-HP	PCC7418_RS02385/PCC7418_RS19225	-356	T, S
**AATTCAACAAAATTATAAGGAGGGGTAAAA**	543130	(-)	gas vesicle protein GvpG	PCC7418_RS02455	-185	S
**GAATGAATTAAAGATTAATTTCTTTTAAAC**	547534	(-)	gas vesicle structural protein GvpA (*gvpA*)-HP-gas vesicle protein GvpN/gas vesicle protein (*gvpN*)-HP-HP-gas vesicle protein K (*gvpK*)-GvpL /GvpF family gas vesicle protein (*gvpL/gvpF*)	PCC7418_RS02490/RS02485/RS02480/RS02475/RS02470/RS02465/RS02460	-103	S, S, S, S, S, J, S
**AATGAATTAAAGATTAATTTCTTTTAAACT**	547535	(+)	GvpL/GvpF family gas vesicle protein (*gvpL/gvpF*)	PCC7418_RS02495	-196	S
**TTATGAAAAAATAATTAACAAAAGATAAAT**	550498	(-)	histidine phosphatase family protein-glucokinase	PCC7418_RS02510/RS02505	-11	G, G
**GAGTTAGAGGATGGTTATAAACATATAAAG**	599343	(-)	(2Fe-2S) binding protein	PCC7418_RS02740	-365	C

**ATTAAAATCCGATCAATCTAAAAATAAGTT**	609687	(+)	ferredoxin nitrite reductase	PCC7418_RS02810	+11	C
**TATGATTTTCAATTTAACTGTACCTATCTT**	612817	(+)	UDP-N-acetylmuramoyl tripeptide D-alanyl-D-alanine ligase	PCC7418_RS02820	-1	M
**TTTTCAATTCTTCTTAAAACCCTGATTAAC**	641750	(-)	Ig like domain containing protein	PCC7418_RS02875	-148	S
**TTTTACTGAATTTTAATTTAAACTTAGAAC**	657612	(+)	ABC transporter ATP binding protein	PCC7418_RS02955	-191	V
**TAATTTATATCTTTTTAAGGTTATTTTAAG**	675681	(-)	CAP domain-containing protein	PCC7418_RS19240	-284	Q
**TTTTACCTGAATTTGTTTTAACCTTAAGTC**	677077	(+)	Uma2 family endonuclease-Uma2 family endonuclease	PCC7418_RS03075/RS03080	-73	S, S
**TGGCTATCTTTATGTTATAATGATTTTAAC**	683216	(-)	Response regulator	PCC7418_RS03100	-432	T
**AATTATCTTCAGTTTAGTTTACGCTAATCT**	728680	(+)	heme oxygenase (biliverdin-producing)	PCC7418_RS03280	-101	C
**ATGAAAAACGTTAGATAAAAACAATTTAAA**	739128	(-)	YvcK family protein	PCC7418_RS03330	-60	S
**ATTTAATTAAATTTACAATTGATTTAAATT**	766012	(+)	CRISPR associated endoribonuclease Cas6-CRISPR associated protein Cas4 type ID-CRISPR associated endonuclease Cas1-CRISPR associated endonuclease Cas2	PCC7418_RS03470/RS03475/RS03480/RS03485	-264	S, L, L, L
**CGTAAACTAAGCATAGTTTGATCTTAATCC**	787591	(+)	WYL domain containing protein	PCC7418_RS03530	-80	K
**AGGCAAGGAGGAGATAAAAGCATGATCAAA**	801527	(-)	LD carboxypeptidase	PCC7418_RS03590	-7	V
**TTTAATGAGTGGTTTAAATTCAGTTGATTT**	805778	(+)	UDP-N-acetylmuramate L-alanine ligase-UDP-N-acetylmuramate dehydrogenase	PCC7418_RS03620/RS03625	-3	M, M
**TTTAATACTTGGTTAGTTCAAGATTATCTT**	814733	(+)	glutathione S-transferase family protein	PCC7418_RS03660	-664	O
**TTTAATAAACTTAAAAATTGTTATTTACAT**	826909	(+)	tRNA (uridine(34)/cytosine(34)/5-carboxymethylaminomethyluridine(34)-2'-O) methyltransferase TrmL (*trmL*)	PCC7418_RS03720	-52	J
**TATTAAATAATAGATCAAAATGAATTTTAT**	832833	(-)	carotenoid biosynthesis protein	PCC7418_RS03740	-19	S
**TGGTTAGTGAGTGATTAAACAATGGTTAGT**	890284	(-)	precorrin 8X methylmutase	PCC7418_RS04085	-190	HK
**GAATTAAGGAACATTTAACTTTTTGCAAAA**	898154	(-)	FeoA domain-containing protein (*feoA*)-Fe(2+) transporter permease subunit FeoB (*feoB*)	PCC7418_RS04115/RS04120	-240	P, P
**AAATAATGCTAAGATTATTATAACATAAAA**	938858	(-)	IS200/IS605 family element transposase accessoryprotein TnpB (*tnpB*)	PCC7418_RS04310	-16	L

**GTTAATCAAGTTTTTACGAATGATTTAGAT**	939282	(+)	MFS transporter	PCC7418_RS04315	-19	G
**TTTAATCCCCTGTTTATGGTAACTTTAGAA**	953364	(+)	fused MFS/spermidine synthase	PCC7418_RS04390	-35	H
**ATTAACTATCAATTAAAATAGCATTTGCTC**	955972	(+)	methyltransferase domain-containing protein	PCC7418_RS04410	-27	Q
**AGGTTAAGGCGAAAATAAACTCATTCCAAG**	957937	(-)	ABC transporter ATP-binding protein	PCC7418_RS04415	-268	P
**GAGTAAACGTTAAGCAAAAATCACATTAAT**	961001	(-)	ABC transporter substrate binding protein-sugar ABC transporter permease arbohydrate ABC transporter permease	PCC7418_RS04430/RS04425/RS04420	-131	G, P, P
**AGCAAAACCATAAGCAATGGGCAAGTTAAA**	964557	(-)	ATP-grasp domain containing protein	PCC7418_RS04445	-352	F
**GATTACCACTCGTTAAATTGGTAATAACAC**	970920	(+)	membrane protein insertase YidC-nucleic acid binding protein	PCC7418_RS04500/RS04505	-105	S, U
**TTTTTAATATAAAATTAATCAATAGTTAAC**	1018379	(-)	DNA starvation/stationary phase protection protein	PCC7418_RS04685	-122	P
**GAAAGTTAAAAGTTAACCATTTCTTAACTT**	1047914	(+)	glycosyltransferase family 4 protein-amylo alpha 1,6-glucosidase	PCC7418_RS04835/RS04840	-439	M, G
**ATCTTAAATTAAGGTTTAAATGAATTTTAA**	1071732	(-)	solanesyl diphosphate synthase	PCC7418_RS04940	-313	H
**CTTAAGATAAGATTAGTCAGTAATTAATCC**	1072305	(+)	uracil-DNA glycosylase	PCC7418_RS04945	-506	L
**CTTATACTAATTTTTTCAAAAGTTTATCCT**	1132472	(+)	Uma2 family endonuclease	PCC7418_RS05220	-136	S
**AGATTATGGGCTGGTTGACAACATATCAAG**	1132816	(-)	phycobilisome rod core linker polypeptide	PCC7418_RS05215	-356	G
**ATAAATATTATTATAAATTAACTTCAAAAT**	1170761	(+)	circadian clock protein KaiC-polyphosphate kinase 1	PCC7418_RS05365/PCC7418_RS05370	-10	F, P
**TTTACCATGGCTATGAATTAACCTTAACCA**	1175011	(+)	MogA/MoaB family molybdenum cofactor biosynthesis protein	PCC7418_RS05380	-145	H
**AAGTTAAGAGTGACTTGATCGGGGTTTAAA**	1196246	(-)	HlyD family type I secretion periplasmic adaptorsubunit	PCC7418_RS05475	-191	M
**TTTAAATATCTTTTTTATTTCTATTTTTAT**	1234207	(+)	L-aspartate oxidase (*nadB*)	PCC7418_RS05635	-202	H
**ATTAATCTCCTAATTATCAACACATCACTA**	1249518	(+)	tRNA (adenosine(37)-N6)-threonylcarbamoyltransferase complex transferase subunit (*tsaD*)	PCC7418_RS05715	-681	J
**TGATTGAGGAGAATTTAAATCAATGTCAAT**	1261043	(-)	arsenate reductase, glutathione/glutaredoxin type (*arsC*)-arsenical resistance protein (*arsH*)	PCC7418_RS05760/RS05755	-346	T, S
**ATTAAACTGCTCATAAGGAAACGTAAATTT**	1360953	(+)	acyl-CoA desaturase	PCC7418_RS06160	-88	I
**AAATAATGCTAAGATTATTATAACATAAA**	1366037	(-)	IS200/IS605 family element transposase accessoryprotein TnpB (*tnpB*)	-	-15	S

**AATTAAAAATAGGAATAAAACTAAGCTAAT**	1371373	(-)	tetratricopeptide repeat protein	PCC7418_RS06205	-12	S
**TTAGAAAAAGAGTTAAATTCTCGTTAAAAT**	1375855	(+)	TIGR04282 family arsenosugar biosynthesis glycosyltransferase	PCC7418_RS06240	-215	S
**GTTATTTTGTTCTTTGTTGGCTGTTAATCA**	1425334	(+)	transposase	PCC7418_RS06495	-115	S
**GTTAATCAACTTTTTACATAAGATTTAGAA**	1439381	(+)	CRISPR associated endonuclease Cas1-CRISPR associated endonuclease Cas2	PCC7418_RS06595/RS06600	-501	L, L
**TGATAAACAATAGGTAAAGTAAGGACTTAC**	1558203	(-)	type I restriction endonuclease subunit R	PCC7418_RS07055	-251	S
**GTTATTTTGTTCTTTGTTGGCTGTTAATCA**	1594235	(+)	transposase	PCC7418_RS07215	-108	S
**AGCTGATGACTAGGTTAAGGCTTTCTTAAT**	1613769	(-)	ABC transporter substrate binding protein	PCC7418_RS07290	-265	E
**CTTTTTCCCACCTTTGACCTGTGTTAAATT**	1623103	(+)	glutathione S-transferase family protein	PCC7418_RS07335	-249	S
**AGATTAAGGAAAATTAAACCTCAACTTAAA**	1664840	(-)	sodium:proton antiporter	PCC7418_RS07540	-114	P
**ATTAAAACAATATTAAAACAAATTTTACTA**	1665092	(+)	ATP-dependent zinc protease	PCC7418_RS07545	-115	O
**TATAATCTTCGCTATAGCTCATTTTTAGTT**	1679832	(+)	response regulator	PCC7418_RS07610	-364	T
**TGTTAAAGACGCAGAAAATCTTATTTTAAA**	1708354	(-)	site-specific DNA-methyltransferase	PCC7418_RS07755	-130	L
**AACTTAATTTCTGAAAAATTCAGGGTTAAT**	1744927	(-)	sedoheptulose 7-phosphate cyclase	PCC7418_RS07910	-329	E
**AAATAAATCCCTGTTAAAGCTCATAAAAAT**	1754941	(-)	response regulator-EAL domain containing response regulator-response regulator	PCC7418_RS07930/RS07935/RS07940	-60	T, T, T
**AAATTAATGTTTCATAATGAGGTGGATTAA**	1766429	(-)	N(2) fixation sustaining protein CowN	PCC7418_RS07995	-601	S
**AGATCATGAGTAATAAATAAATAGGTTAAA**	1767932	(-)	D-alanyl-D-alanine carboxypeptidase/D-alanyl-D-alanine endopeptidase	PCC7418_RS08000	-120	M
**TTCATAATTAATCGTTAATAAATGATTAAG**	1794333	(-)	ABC transporter substrate-binding protein	PCC7418_RS08120	-6	E
**ATTTTTTTGTTTTTAATTATCCCTTGACTT**	1810041	(+)	ATP phosphoribosyltransferase regulatory subunit	PCC7418_RS08205	-314	E
**TTTTCCCTCACCTTAGATTTAAGTTTATTA**	1811102	(+)	undecaprenyldiphospho-muramoylpentapeptide beta-N-acetylglucosaminyltransferase	PCC7418_RS08210	-449	M
**TTCTAAATCATTCGTAAAAAGTTGATTAAC**	1871218	(-)	copper-translocating P-type ATPase	PCC7418_RS08475	-151	P
**TTTAAGCATGACCTTACCCCTAATTATCCT**	1889348	(+)	NAD(P)H-quinone oxidoreductase subunit 4	PCC7418_RS08560	-648	C
**TTTAATTGTAAATTTTCTTATTTTTTAGTT**	1900730	(+)	glutathione synthase (*gshB*)	PCC7418_RS08590	-93	H

**TGCTTGTGGTAAAGAAAAGGTTATGTTAAA**	1919258	(-)	phosphate ABC transporter substrate binding protein PstS (*pstS*)-phosphate ABC transporter permease subunit PstC (*pstC*)-phosphate ABC transporter permease PstA (*pstA*)-phosphate ABC transporter ATP binding protein PstB (*pstB*)	PCC7418_RS08670/RS08665/RS08660/RS08655	-256	P, P, P, P
**GTTAATCACAGAGAAAATTAATCTTAAATT**	1932831	(+)	endonuclease MutS2	PCC7418_RS08735	-136	L
**GATTAAAGTTTTGATAAAGTCTTGATCTAG**	1942813	(-)	cysteine synthase A (*cysK*)	PCC7418_RS08775	-162	E
**ATTTATGTTATAATAATTTTACCATTATTT**	1970080	(+)	IS200/IS605 family element transposase accessoryprotein TnpB (*tnpB*)	PCC7418_RS08950	-40	S
**ATTGAACCTTTATTAAACTCGGTTTATCAA**	1996480	(+)	response regulator-chemotaxis protein CheW (*cheW*)	PCC7418_RS09080/RS09085	-696	KT, NT
**TTGTAATGGGCGGGTTTAGGAATGGTTAAA**	2056920	(-)	glycosyltransferase family 2 protein	PCC7418_RS09320	-565	M
**GAGCTAAGAAATGATAATGCTGTTCTAAAC**	2134623	(-)	cobalt precorrin 8X methylmutase/bifunctional cobalt-precorrin-7 (C(5)) -methyltransferase cobalt precorrin-6B (C(15)) methyltransferase (*cbiE*)	PCC7418_RS09630/RS09625	-343	H, T
**TTTAAAAAAGTAATTGTCTCCCGTTTTCCA**	2186011	(+)	late competence development ComFB family protein	PCC7418_RS09865	-39	S
**AGGTAATGACGATATTAATCCCATTATAAA**	2244285	(-)	RNA methyltransferase	PCC7418_RS10105	-172	L
**CGGTAAACGTAAAATTAGCCCCATGTAAAT**	2251376	(-)	5-(carboxyamino)imidazole ribonucleotide synthase	PCC7418_RS10145	-205	F
**AATAGTCTCATTTTTATTTTTAATTTTTAT**	2285162	(+)	alanine:cation symporter family protein	PCC7418_RS10255	-415	U
**ATTATTATAGATATTATCTCCGGCTAAAAT**	2323958	(+)	serine O-acetyltransferase	PCC7418_RS10410	-391	E
**TTAAATTTAATCATTACTTTAGGTTTAAGT**	2388578	(+)	class 1 fructose-bisphosphatase	PCC7418_RS10725	-182	G
**GATAAAAAGTTAAATCAACTAAAGATAAAT**	2403390	(-)	citramalate synthase	PCC7418_RS10785	-453	E
**TTTTGCCTAAATTTTATTTTCTATTAACCT**	2442208	(+)	PAS domain S-box protein	PCC7418_RS19415	-102	T
**AGTAAAAACAATCTTTAAATTGAATTAAAA**	2467390	(-)	AAA family ATPase	PCC7418_RS11110	-140	O
**AATAAAATATTACGTAAACCATAGGCTAAA**	2476471	(-)	lipoprotein signal peptidase	PCC7418_RS11185	0	MU
**TTTAACTGCAATTTTAATTTTAATTGGCTT**	2487840	(+)	glucosylglycerol 3-phosphatase	PCC7418_RS11220	-321	S

**GATTTAAAATAAAGATTTGCTAAGGTTAAA**	2519448	(-)	saccharopine dehydrogenase NADP-binding domain containing protein	PCC7418_RS11355	-19	E
**AAATTAAAAGAAGTTTTAAGATTTAATATA**	2523201	(-)	cytochrome ubiquinol oxidase subunit I-cytochrome d ubiquinol oxidase subunit II (*cydB*)	PCC7418_RS11365/PCC7418_RS11370	-66	C, C
**TTTTACGAAGAATTAAACTCATTTTTACCG**	2523301	(+)	hemerythrin family protein	PCC7418_RS11375	-80	P
**TTTAAATTTTTTATAACAATTGATTGATCA**	2657664	(+)	EAL domain containing protein	PCC7418_RS19435	-99	T
**GTATGAACGTTAATTAAAGGCATAATTAAT**	2669847	(-)	macrophage migration inhibitory factor family protein	PCC7418_RS12020	-7	S
**ATTAATCATTTGCTTACTCAGGATTAAACT**	2686183	(+)	Crp/Fnr family transcriptional regulator	PCC7418_RS12095	-165	K
**AATAATGAAACTTTTTCCTCTTCTTTTACT**	2708076	(+)	NADAR family protein	PCC7418_RS12195	-219	O
**TTTATTTTTATGTTATAATAATCTTAGCAT**	2735781	(+)	IS200/IS605 family element transposase accessoryprotein TnpB (*tnpB*)	PCC7418_RS12340	-45	S
**ATTAAGAAGAACTTAACGAATCTTTAATTT**	2791405	(+)	Crp/Fnr family transcriptional regulator	PCC7418_RS12605	-41	K
**TTTAACTTATATTTTTATTTCCCATGACTT**	2871984	(+)	ureidoglycolate lyase	PCC7418_RS13060	-255	F
**TTTAACTTTTTGTTTATTGCCACCTTTCCT**	2922699	(+)	llutamate cysteine ligase (*gshA*)	PCC7418_RS13320	-19	S
**CACTGAAAAACAAAAAAAGGCTAGTTTAAT**	2930864	(-)	lysine tRNA ligase	PCC7418_RS13445	-191	J
**TGTTCAAAACTACGCAATGTGAAAATTAAT**	2954730	(-)	ribosome maturation factor RimM (*rimM*)	PCC7418_RS13700	-97	J
**ATTTATATAATGATTAATTGACTTTGTTTT**	3006109	(+)	polysaccharide pyruvyl transferase family protein	PCC7418_RS13700	-127	S
**AGATTATTTCTGATTAAAACGAATACTAAA**	3128024	(-)	translocation/assembly module TamB domain-containing protein	PCC7418_RS14210	-32	U
**TACTTAAGTATAGTATAAGATTATAATAT**	3138188	(-)	YqhA family protein- DUF202 domain containing protein	PCC7418_RS14255/RS14260	-89	S, S
**AGTAAGAGGTGAATTAAAGGGGGGATCAAA**	3146602	(-)	formylglycine generating enzyme family protein	PCC7418_RS14295	-69	S
**TTTAAAAAATAATTAATTTTCTTCCAACAT**	3179396	(+)	DMT family transporter	PCC7418_RS14440	-219	EG
**ATTTATCTAAATATAAAACTGAATTAAATA**	3191656	(+)	2Fe-2S iron sulfur cluster binding domain containing protein	PCC7418_RS14510	-279	C
**ATATAAAACTGAATTAAATATAATTATAAC**	3191666	(-)	M28 family peptidase	PCC7418_RS14505	-8	S
**ATTGATGACTTATTAATTATCAATTAAGTT**	3224851	(+)	o-succinylbenzoate synthase	PCC7418_RS14660	-11	M
**GATAATATCATGATTATTTTTTTCTTATTA**	3244402	(+)	glycosyltransferase	PCC7418_RS14775	-254	V

**AAGCTAAACGTAGATAGAGTTGAGTAAAAA**	3247808	(-)	glycosyltransferase-sulfotransferase	PCC7418_RS14780/RS14785	-94	M, S
**CTTTATCTAATTTTGATCACTAGTTTTATA**	3275429	(+)	DNA phosphorothioation system sulfurtransferase DndC (*dndC*)	PCC7418_RS14930	-56	EH
**CTTAGAATTTAGTAAATCTTAACTTAATT**	3309133	(+)	BCCT family transporter	PCC7418_RS15085	-305	M
**ATTAGAATCAGTTTAAACTAAGCTCAATGT**	3332712	(+)	nickel-type superoxide dismutase maturation protease	PCC7418_RS15220	0	U
**GTTAATTGTTGCTTAAAATTACGTTTGCAT**	3404962	(+)	peptide chain release factor 1 (*prfA)*	PCC7418_RS15625	-65	J
**ATAATAAAGGGTGGTTATATGAAAGTAAAG**	3410009	(-)	DNA (cytosine-5-) methyltransferase	PCC7418_RS15640	-359	H
**CTCTAAAAAGTAATCTAAAAAAAATTTAAT**	3427651	(-)	TdeIII family type II restriction endonuclease	PCC7418_RS15720	-49	L
**AGGTTAGAAGCAACTAAAGTTTTCATAAAT**	3454152	(-)	phycobilisome linker polypeptide	PCC7418_RS15845	-4	H
**AAACAAAAAGAAAGTTAAATTTTTATAAAC**	3455723	(-)	phycocyanin subunit beta	PCC7418_RS15855	-246	C
**TTTAATTTCTCCTTAATAGGATATTAGTTA**	3467427	(+)	translation initiation factor IF-2	PCC7418_RS15920	-123	S
**ATTGACTTTTTGTTAGACTGAATTTAGTTT**	3484159	(+)	IS200/IS605 family transposase	PCC7418_RS15965	-48	S
**TTTTTTGTCAACTTAACCTTCCCTTTACTT**	3516392	(+)	PstS family phosphate ABC transporter substrate binding protein (*pstS*)-phosphate ABC transporter permease subunit PstC (*pstC*)-phosphate ABC transporter permease PstA (*pstA*)-phosphate ABC transporter ATP binding protein PstB (*pstB*)	PCC7418_RS16135/RS16140/ RS16145/RS16150	-147	P, P, P, P
**TCATTAAACATCGAATAAACTTTGATTAAA**	3579899	(-)	LmeA family phospholipid binding protein	PCC7418_RS16405	0	S
**ATTAAACATCGAATAAACTTTGATTAAAAT**	3579901	(+)	rRNA pseudouridine synthase	PCC7418_RS16410	-53	-
**ATTTTTTTGAGCATTATTTGAGCATTATTT**	3596661	(+)	response regulator-chemotaxis protein CheW (*cheW*)	PCC7418_RS16505/RS16510	-120	KT, NT
**ATTAATTTTTATTAAGATTATGTTTTTTTT**	3799356	(+)	class I SAM-dependent methyltransferase	PCC7418_RS17370	-383	H
**ATTTATTGATGTATTTTTTTGTCTTAACCC**	3774649	(+)	alkaline phosphatase	PCC7418_RS17240	-76	P
**ATTAATCATTCTCTATCCTCCAAATAACCA**	3842069	(+)	pentapeptide repeat-containing protein	PCC7418_RS17560	-59	S
**ATTATTATTTTTGTTATTCACGCTTAACCT**	3866970	(+)	transposase	PCC7418_RS17665	-417	Q
**ATTAACTTGCAATTATTTATCCCTTGATC**	3887039	(+)	Hemolysin type calcium binding protein	PCC7418_RS17775	-48	S

**TTTAGGCTGTAATTAAACTGATGTCAACAA**	3896679	(+)	IS200/IS605 family element transposase accessoryprotein TnpB (*tnpB*)	PCC7418_RS17810	-155	S
**ATTATAAAAATAATTTAAAATGAATATAAA**	3944455	(-)	HindVP family restriction endonuclease	PCC7418_RS18040	0	L
**TTTTTAAGCGAAATTTATCAAGGAGAAAA**	3960433	(+)	glycogen debranching protein GlgX (*glgX*)	PCC7418_RS18135	-167	G
**GTTAAATCAGAGTTAACCCATTATTAAATT**	3992761	(+)	methyl-accepting chemotaxis sensory transducer	PCC7418_RS18275	-107	NT
**TGGATAATAGCATATAAAGACTTAATAAAT**	4015538	(-)	NAD(P)-dependent oxidoreductase	PCC7418_RS18405	-406	GM
**ATAAAAAAGATTAATAATAGTAAGGTAAAA**	4030328	(+)	glycosyltransferase	PCC7418_RS18495	-129	J
**GATAAATTCTTCTTTTACTCTTCTTTTACA**	4066330	(+)	D-alanyl-D-alanine carboxypeptidase family protein	PCC7418_RS18675	-233	M
**TATAACCTATCATTTATTGTCAAATAACCA**	4074906	(+)	ABC transporter ATP binding protein-ABC transporter permease	PCC7418_RS18715/RS18720	-61	V
**TAGTTAATTTTAATTAATTTATTAATAATT**	4087245	(+)	Na-Ca exchanger/integrin-beta4	PCC7418_RS18775	-319	S
**AAATCATCAATTCGTTAAATTCAATTTAAT**	4115030	(-)	SDR family oxidoreductase	PCC7418_RS18900	-148	IQ
**AGTTTAAATTTATAATCAGTTCAGTTTAAT**	4163771	(-)	SHOCT domain-containing protein	PCC7418_RS19070	-26	L
**GCTAACCTTTTTATTATCTTAACTTAGATT**	4174392	(+)	GNAT family N-acetyltransferase	PCC7418_RS19130	-255	K

Supplementary Table 3. Fur regulon prediction. Fur boxes sequences, their position in the genome, orientation, the genes that are controlling (the Fur regulon), locus tag from these genes, the distance of the box from the gene and the gen function from single-letter of functional (COGs) are presented.

Fur boxes sequences	Position	Orientation	Fur regulon (genes)	Locus_tag	Distance	COG
**GTTAATAAATATTTTTTAC**	1663	(+)	chromosomal replication initiator protein DnaA (*dnaA*)	PCC7418_RS00025	-472	L
**GTTACAAAATATTTAGAAT**	50660	(-)	alpha amylase	PCC7418_RS00215	-58	S
**ATCTAAAGTTTTTATAAAA**	54620	(-)	PAS domain containing protein	PCC7418_RS00230	-123	T
**AATTCAAGAAATTTTAAAT**	63574	(-)	recombination protein RecR	PCC7418_RS00270	-277	L
**ATTAAAAACCATTTTTTAT**	89952	(+)	esterase like activity of phytase family protein	PCC7418_RS20370	-355	F
**TTTGTAAATTTTTATCATC**	104461	(-)	SDR family oxidoreductase	PCC7418_RS00415	-85	G
**ATCTAAAATTAATCATAAT**	114408	(-)	ketol-acid reductoisomerase	PCC7418_RS00460	-250	E
**AATTTGGATATTTTTTAAT**	116549	(+)	glycosyltransferase family 2 protein	PCC7418_RS00475	-38	M
**ATTTTGATTATATATAAAT**	148165	(+)	prepilin-type N-terminal cleavage/methylation domain-containing protein	PCC7418_RS00635	-172	U
**ATTTATAAATATTATCAAT**	199345	(+)	energy transducer TonB (*tonB*)	PCC7418_RS00895	-13	M
**ATTTATAAATATTATCAAT**	199345	(-)	MotA/TolQ/ExbB proton channel family protein	PCC7418_RS00890	-192	U
**ATTGCAAATATTTTTTGAT**	200598	(+)	AraC family transcriptional regulator	PCC7418_RS00900	-38	K
**ATTTTTCTTTTATTTTAAT**	247069	(+)	DUF3298/DUF4163 domain containing protein	PCC7418_RS01105	-454	S
**ATTAAGTAATATTACAAAA**	379625	(-)	heavy metal translocating P-type ATPase	PCC7418_RS01685	-283	P
**AATATTTATATTTCTTAAT**	510372	(+)	4-alpha-glucanotransferase	PCC7418_RS02325	-227	G
**ATAAAAATTTAATAATAAT**	524171	(-)	response regulator	PCC7418_RS02385	-374	T
**ATTGATAATATTTATTAAC**	559257	(-)	coenzyme F420 hydrogenase	PCC7418_RS02560	-101	C
**TTTTATGATTATTATCAAT**	690115	(+)	ShlB/FhaC/HecB family hemolysin secretion/activation protein	PCC7418_RS03120	-17	M
**ATTAATAAACTTTTTTAAC**	746975	(+)	photosystem I reaction center protein subunit XI-photosystem I reaction center subunit VIII	PCC7418_RS03390	-246	S
**ATTTAATTAAATTTACAAT**	766012	(+)	CRISPR associated endoribonuclease Cas6	PCC7418_RS03470	-242	S
**ATTGACAAAATTTCCTAAT**	789753	(-)	photosystem II q(b) protein	PCC7418_RS03535	-74	C

**ATTGAAAGTCATTCTTATT**	898088	(-)	ferrous iron transport protein A-Fe (2+) (*feoA*)-transporter permease subunit FeoB (*feoB*)	PCC7418_RS04120/ RS04115	-93	P, P
**ATTAGAAATTTTTCTAAAA**	1004388	(-)	ISAs1 family transposase	PCC7418_RS20420	0	S
**AATGTTAAATTTTTATAAA**	1169323	(+)	KaiA family protein	PCC7418_RS05355	-185	S
**ATTTATAACTTTTCTCTAT**	1179409	(+)	MFS transporter	PCC7418_RS05400	-207	G
**ATTTTTGATAATTATTAAT**	1210254	(+)	HP	PCC7418_RS05535	-184	P
**GTTGAAATAGATTCTTATT**	1244480	(-)	adenylyl-sulfate kinase	PCC7418_RS05670	-147	P
**ATTAGAAATTTTTCTAAAA**	1277716	(-)	ISAs1 family transposase	PCC7418_RS05850	0	S
**ATTTTAATTTTTTTGTTAT**	1541282	(+)	HEAT repeat domain-containing protein	PCC7418_RS07010	-88	C
**ACTAATATTTTTTCTAAAT**	1613963	(-)	solute-ABC transporter substrate-binding protein	PCC7418_RS07285	-458	E
**ATTAAAATAAAATCTAATC**	1626439	(-)	CDP-diacylglycerol--glycerol-3-phosphate 3-phosphatidyltransferase	PCC7418_RS07355	-120	I
**ATTAACAAATTATAAAATT**	1638172	(-)	sensor domain-containing diguanylate cyclase	PCC7418_RS07405	-11	P
**TTATAAAATTATTTACAAT**	1773346	(-)	2-carboxy-1,4-naphthoquinone phytyltransferase (*menA*)	PCC7418_RS08020	-216	H
**ATTACAAAAGATTCTCAAC**	1846361	(-)	ferrous iron transport protein A-Fe (2+) (*feoA*)-transporter permease subunit FeoB (*feoB*)	PCC7418_RS08340/ RS08335	-31	P, P
**AATTATTAAAATTATTAAT**	1978529	(+)	photosystem II protein PsbX (*psbX*)	PCC7418_RS08985	-145	C
**GTTCATAATATTTTATAAT**	2010967	(+)	DUF2207 domain-containing protein	PCC7418_RS09130	-396	S
**TTTTAGTATATTTCTCATT**	2201702	(+)	30S ribosome-binding factor RbfA (*rbfA*)	PCC7418_RS09935	-165	H
**ATTGACAACTATTTTTAGT**	2226486	(+)	methyl-accepting chemotaxis protein	PCC7418_RS10065	-669	L
**ATTGCAATTAAATTTAAAA**	2226728	(-)	ferrous iron transport protein A-Fe (2+) (*feoA*)-transporter permease subunit FeoB (*feoB*)	PCC7418_RS10060/ RS10055	-316	P
**TTTTGTAAATTTTTGTAAA**	2234872	(+)	glutamate-1-semialdehyde-2,1-aminomutase (*gsaI*)	PCC7418_RS10080	-177	H
**TTTGTAAATTTTTGTAAAT**	2234873	(+)	magnesium chelatase subunit H (*chlH*)	PCC7418_RS10075	-214	H
**TTTTAAAATAAAAAATAAT**	2376830	(-)	hydrogenase maturation protease	PCC7418_RS10660	-207	C
**ATTGCTATTTATTCATAAT**	2425245	(-)	DUF1643 domain-containing protein	PCC7418_RS19410	-256	S

**TTTTAGAAAAATTTCTAAT**	2459685	(+)	ISAs1 family transposase	PCC7418_RS11060	0	S
**TTTGTTATTTATTCTTAGT**	2584479	(+)	HP	PCC7418_RS11685	-270	S
**AATACAAAAATTTGTTAAA**	2609963	(-)	ABC transporter ATP-binding protein	PCC7418_RS11810	-546	V
**ATTTTTAATTTTTTGTTAA**	2785156	(+)	cobalamin biosynthesis protein (*cobW)*	-	-64	S
**ATTGCAAATTTTTTTTATT**	2791453	(+)	Crp/Fnr family transcriptional regulator	PCC7418_RS12605	0	K
**ATTAAGAAAAATTAATATT**	2858373	(+)	argininosuccinate synthase	PCC7418_RS13005	0	E
**ATTGTTATTTATTATTTGT**	2864252	(+)	6-pyruvoyltetrahydropterin synthase	PCC7418_RS13025	-312	H
**ACTGGAAAAAATTATCAA A**	2874763	(-)	methyltransferase domain-containing protein	PCC7418_RS1306	-194	L
**ATTGAGATATTTTATCGAT**	2883764	(+)	transcription-repair coupling factor	PCC7418_RS13105	-64	L
**ATTTTGAACAAATTTCAAT**	2933424	(+)	SpoIID/LytB domain-containing protein	PCC7418_RS13340	-50	D
**GTTTTAAAAAATAATTAAT**	3179394	(-)	photosystem II reaction center protein Ycf12	PCC7418_RS14435	-25	S
**ATTAAATATAATTATAACT**	3191678	(-)	M28 family peptidase	PCC7418_RS14505	-21	S
**GTTGAGATTAATTTTCATT**	3379840	(-)	PAS domain-containing protein	PCC7418_RS15425	-46	T
**ATTAATAAAGTTTTGCAAA**	3441389	(+)	glutamate-5-semialdehyde dehydrogenase	PCC7418_RS15780	-102	E
**GTTGCTAATTTTTAGCAAA**	3460255	(-)	bifunctional phosphoribosylaminoimidazolecarboxamide formyltransferase/IMP cyclohydrolase (*purH*)	PCC7418_RS15885	-45	F
**AATTGGAAATTTTTTCAGT**	3692765	(+)	peptidase S13	PCC7418_RS16905	-39	S
**ATTGCAATTTAATTTCAAT**	3725995	(-)	alpha/beta hydrolase	PCC7418_RS17030	-21	S
**ATTGTCAATTTTTTGTAAT**	3727770	(+)	HU family DNA binding protein	PCC7418_RS17045	-245	L
**ATTATAAAAAACTATTAAC**	3774691	(+)	alkaline phosphatase	PCC7418_RS17240	-45	S
**GTTAATTTTAATTTTTAAT**	3840660	(+)	sensor protein	PCC7418_RS17555	-1	S
**ATTGCAAAAGATATTCAAT**	3885410	(-)	chlorophyll a/b binding light-harvesting protein-chlorophyll a/b binding light-harvesting protein	PCC7418_RS17760	-16	S
**TTTGAAATTAACTTGCAAT**	3887033	(+)	hemolysin type calcium binding protein	PCC7418_RS17775	-54	V

**TATAAAAATAATTTAAAAT**	3944457	(-)	HindVP family restriction endonuclease	PCC7418_RS18040	0	V
**GATAAAAAAGATTAATAAT**	4030327	(-)	glycosyltransferase family 2 protein	PCC7418_RS18475	-127	M
**ATTGAAATAAATTGTCAAC**	4047761	(+)	uracil phosphoribosyltransferase	PCC7418_RS18570	-188	F
**ATTGAAATAAATTGTCAAC**	4047761	(-)	carotene isomerase	PCC7418_RS18565	-141	F
**ATTTAGCAAAATTATTAAA**	4072511	(+)	magnesium-protoporphyrin IX monomethyl ester anaerobic oxidative cyclase	PCC7418_RS18705	-77	H
**TTTGAGAATATTTCTTTTT**	4095529	(+)	PadR family transcriptional regulator (*padR*)	PCC7418_RS18830	-139	K

Supplementary Table 4. NtcA regulon prediction. NtcA boxes sequences, their position in the genome, orientation, the genes that are controlling (the NtcA regulon), locus tag from these genes, the distance of the box from the gene and the gen function from single-letter of functional (COGs) are presented.

**NtcA boxes sequences**	**Position**	**Orientation**	**NtcA regulon (genes)**	**Locus_tag**	**Distance**	**COG**
**TGTAATATTTGTTAAA**	193565	(-)	phosphoribosylformylglycinamidine cyclo-ligase (*purM*)	PCC7418_RS00865	-14	F
**TGTAACATTCGATACA**	433503	(+)	DUF4278 domain-containing protein	PCC7418_RS01970	-53	P
**TGTAAATTTTGATACC**	435747	(+)	PstS phosphate ABC transporter substrate-binding protein (*pstS*)	PCC7418_RS01980	-166	P
**TGTAAATTTTGATACC**	435748	(-)	adenylyl-sulfate kinase (*cysC*)	PCC7418_RS01975	-3	S
**CGTATATTATGATACA**	609571	(+)	ferredoxin--nitrite reductase (*nirA*)	PCC7418_RS02810	-89	C
**TGTAATAGTAAATAGA**	676478	(+)	Uma2 family endonuclease	PCC7418_RS03075	-18	S
**TGTATCTATTGTTACA**	857460	(-)	DUF4278 domain-containing protein	PCC7418_RS03870	-116	S
**TGTATCAAATATTACT**	1222068	(-)	2-oxo acid dehydrogenase subunit E2	PCC7418_RS05590	-159	C
**TGTATCAAATATTACT**	1222069	(+)	response regulator	PCC7418_RS05595	-100	T
**AGTAAAAACTGCTACA**	1299552	(+)	hybrid sensor histidine kinase/response regulator	PCC7418_RS05925	-195	T
**TGTATTGTTTTCTACA**	1331526	(-)	helix turn helix domain-containing protein	PCC7418_RS06050	-183	C
**AGTAGTTTTTGTTACA**	1381373	(+)	glutamate synthase large subunit *(gltB*)	PCC7418_RS06265	-342	E
**TGTATTTTTCGATACA**	1487910	(+)	ammonium transporter (*amt*)	PCC7418_RS06755	-145	P
**TGTATCTTTTTATACT**	1496086	(+)	Crp/Fnr family transcriptional regulator	PCC7418_RS06790	-54	K
**TATAACTTTTTTTACA**	1502278	(-)	elongation factor G (*fusA*)-elongation factor Tu (*tuf*)	PCC7418_RS06815/ RS06810	-82	J, J
**AGTAACAATTGATACA**	1504147	(-)	cupin domain-containing protein-iron-sulfur cluster assembly accessory protein	PCC7418_RS06835	-14	G
**TGTTTTTTCTGATACA**	1613632	(-)	solute-ABC transporter substrate-binding protein	PCC7418_RS07290	-120	E

**TGTATCATTTGTTACA**	1907277	(-)	pyridoxal phosphate-dependent aminotransferase	PCC7418_RS08620	-77	E
**TGTAGCGTTTGTTACT**	1946907	(+)	nitrogenase cofactor biosynthesis protein (*nifB*)	PCC7418_RS08815	-629	C
**AGTATTACTAAATACA**	1963729	(+)	molybdate ABC transporter substrate-binding protein (*modA*)	PCC7418_RS08910	-352	P
**TGTATAAATCGCTACA**	1981610	(-)	ammonium transporter (*amt*)	PCC7418_RS08960	-56	P
**AGTAACATTAAATACA**	2022577	(-)	SulP family inorganic anion transporter	PCC7418_RS09170	-90	P
**AGTAACATTAAATACA**	2022578	(+)	TIGR03279 family radical SAM protein	PCC7418_RS09175	-78	C
**TGTAGTTGCAATTACT**	2077841	(-)	sugar transferase	PCC7418_RS09405	-75	M
**TGTAGCTGATAATACC**	2201041	(-)	orange carotenoid-binding protein	PCC7418_RS09925	-53	S
**TCTAACAAAAGTTACA**	2260357	(+)	photosystem II chlorophyll-binding protein CP47 (*psbB*)	PCC7418_RS10180	-161	S
**TGTAACTTTAGATACG**	2301793	(-)	alanine:cation symporter family protein	PCC7418_RS10335	-68	E
**TGTAACTTGTATTACA**	2496434	(+)	phosphodiesterase	PCC7418_RS11260	-145	E
**TGTATTTAATGTTACA**	2725914	(-)	type I glutamate--ammonia ligase *(glnN*)	PCC7418_RS12280	-72	E
**TGTAGTTGAGGTTACA**	2977247	(+)	NINE protein	PCC7418_RS13560	-195	E
**AGTAGCAATTATTACA**	3197191	(-)	sodium:glutamate symporter (*gltS*)	PCC7418_RS14540	-106	E
**TGTATTTTTTGATACA**	3288086	(-)	MinD/ParA family protein	PCC7418_RS14970	-204	D
**AGTAAAATAAAATACA**	3502170	(-)	rhodopsin	PCC7418_RS16035	-294	S
**TGTACAAAATATTACA**	3542559	(-)	tetratricopeptide repeat protein	PCC7418_RS16280	-70	S
**TGTTATTTACGATACA**	3690483	(-)	[acyl-carrier-protein] S-malonyltransferase (*fabD*)	PCC7418_RS16890	-7	I

**TGTATTCACTAATACA**	3817904	(+)	response regulator	PCC7418_RS17455	-61	T
**AGTAATTATTGCTACA**	3962735	(+)	transcriptional repressor (*fur*)	PCC7418_RS16465	-7	T
**AGTATCTTTCGTTACA**	4051198	(-)	L-histidine N(alpha)-methyltransferase (*egtD*)	PCC7418_RS18585	-41	S
**CGTATCAAGAGATACA**	4130890	(+)	ATP-grasp domain-containing protein	PCC7418_RS18945	-144	S
